# Distinct Developmental Origins Manifest in the Specialized Encoding of Movement by Adult Neurons of the External Globus Pallidus

**DOI:** 10.1016/j.neuron.2015.03.007

**Published:** 2015-04-22

**Authors:** Paul D. Dodson, Joseph T. Larvin, James M. Duffell, Farid N. Garas, Natalie M. Doig, Nicoletta Kessaris, Ian C. Duguid, Rafal Bogacz, Simon J.B. Butt, Peter J. Magill

**Affiliations:** 1 Medical Research Council Brain Network Dynamics Unit, Department of Pharmacology, University of Oxford, Oxford OX1 3QT, UK; 2 Oxford Parkinson’s Disease Centre, University of Oxford, Oxford OX1 3QX, UK; 3 Wolfson Institute for Biomedical Research and Department of Cell and Developmental Biology, University College London, London WC1E 6BT, UK; 4 Centre for Integrative Physiology, School of Biomedical Sciences, University of Edinburgh, Edinburgh EH8 9XD, UK; 5 Nuffield Department of Clinical Neurosciences, University of Oxford, Oxford OX3 9DU, UK; 6 Department of Physiology, Anatomy and Genetics, University of Oxford, Oxford OX1 3QX, UK

## Abstract

Transcriptional codes initiated during brain development are ultimately realized in adulthood as distinct cell types performing specialized roles in behavior. Focusing on the mouse external globus pallidus (GPe), we demonstrate that the potential contributions of two GABAergic GPe cell types to voluntary action are fated from early life to be distinct. Prototypic GPe neurons derive from the medial ganglionic eminence of the embryonic subpallium and express the transcription factor Nkx2-1. These neurons fire at high rates during alert rest, and encode movements through heterogeneous firing rate changes, with many neurons decreasing their activity. In contrast, arkypallidal GPe neurons originate from lateral/caudal ganglionic eminences, express the transcription factor FoxP2, fire at low rates during rest, and encode movements with robust increases in firing. We conclude that developmental diversity positions prototypic and arkypallidal neurons to fulfil distinct roles in behavior via their disparate regulation of GABA release onto different basal ganglia targets.

## Highlights

• Arkypallidal and prototypic GPe cells have distinct origins and transcriptional codes
• Arkypallidal neurons rapidly and robustly increase firing rate at movement onset
• Movement is accurately encoded by single arkypallidal or prototypic neurons
• Two GPe cell types are fated to affect different targets in distinct ways in behavior

## Teaser

Cell type specification in the developing brain informs on neuronal function in adult behavior. Dodson et al. demonstrate that distinct embryonic origins and transcriptional codes of two types of GABAergic pallidal projection neuron are reflected in their divergent encoding of movement.

## Introduction

Neuronal cell type is programmed during brain development according to a tailored, combinatorial transcription factor code, with some code elements also being required to maintain the identities of postmitotic neurons throughout life (Deneris and Hobert, 2014). Past work, and particularly that on laminated structures like the spinal cord, retina, and cortex, has begun to unravel how transcriptional codes dictate the generation, specification, migration, and synaptic maturation of distinct neuronal populations (Arber, 2012; Jessell, 2000; Kepecs and Fishell, 2014; Molyneaux et al., 2007; Siegert et al., 2012). The extent to which these developmental processes map onto the neural coding of adult behavior in “real time” is unclear, but will likely vary in a cell-type-specific manner. The exploitation of transcriptional codes to resolve the contributions of different cell types to behavior holds particular promise in non-laminated brain structures comprising different projection cell types that intermingle and share the same neurotransmitter phenotype, as is the case with the external globus pallidus (GPe).

The GPe is a key component of basal ganglia circuits that govern movement and cognition, and is considered to have uniform cellular constituents in classical schemes of basal ganglia organization (DeLong, 1990; Mink, 1996; Smith et al., 1998; Wichmann and DeLong, 1996). Despite the great utility of such conceptual schemes, the GPe is more realistically viewed as being comprised of different populations of GABAergic neuron (Kita, 2007). This is especially evident in the Parkinsonian GPe wherein two major GABAergic cell types, “prototypic” and “arkypallidal” neurons, exhibit distinct firing under anesthesia (Mallet et al., 2012). Importantly, prototypic and arkypallidal neurons also project to distinct sets of basal ganglia targets; the former cell type innervates the subthalamic nucleus (STN) and basal ganglia output nuclei, whereas the latter only innervates the striatum (Mallet et al., 2012). Cellular heterogeneity in the adult GPe might well stem from early stages of brain development. For example, most (but not all) GPe neurons derive from the medial ganglionic eminence (MGE) of the embryonic subpallium (Flandin et al., 2010; Nóbrega-Pereira et al., 2010). However, diverse ontogeny has yet to be framed in the context of functionally defined cell types in the adult, dopamine-intact GPe.

To link the early-life development of GPe neurons to their dynamic encoding of behavior in adults, we traced the embryonic origins of prototypic and arkypallidal neurons and also recorded the firing of individual, molecularly identified neurons in awake mice. We show that prototypic and arkypallidal neurons are derived from distinct pools of subpallial progenitors and are endowed with cell-type-specific transcriptional codes. This diversity translates to the adult GPe wherein the two cell types exhibit distinct firing rates/patterns at rest and during the execution of voluntary movements, suggesting they perform dedicated, complementary roles according to their lineages.

## Results

### Two Major Types of GPe Neuron Are Delineated by Transcription Factor Expression

**Figure 1:**
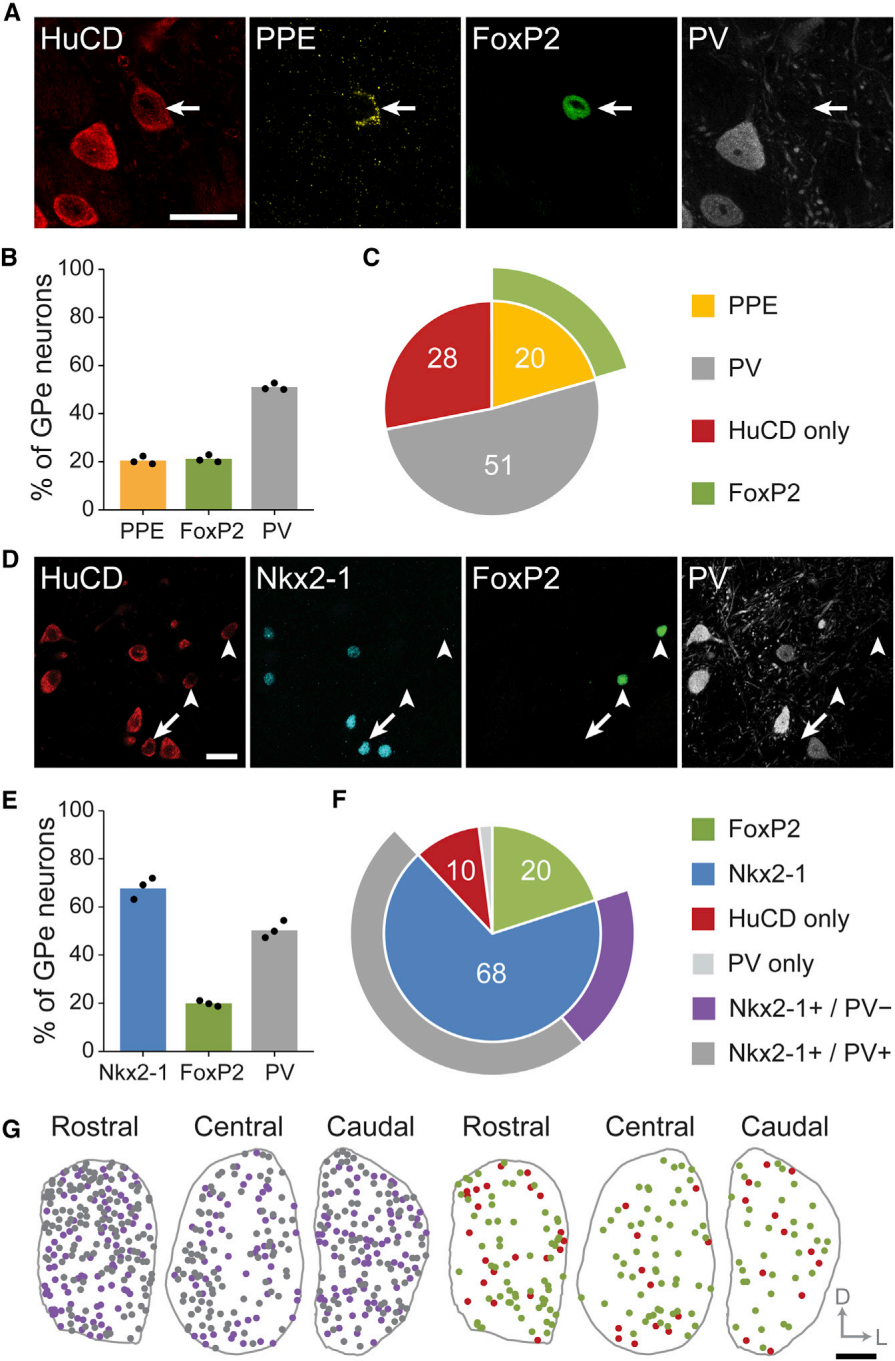
Molecular Heterogeneity of Neurons in the Adult GPe Immunofluorescence identification of arkypallidal neurons (A)–(C) and prototypic neurons (D)–(F). (A) The pan-neuronal marker HuCD was used to label all GPe neurons. Preproenkephalin (PPE)-expressing GPe neurons (arrow) also express FoxP2, but not parvalbumin (PV). (B) Mean expression profiles for PPE, FoxP2, and PV. Filled circles in this and subsequent profiles represent counts from individual animals. (C) Proportions of GPe neurons (i.e., all HuCD+ neurons) expressing different molecular markers. In this and subsequent pie charts, outer segments represent expression overlap of inner populations with another marker (e.g., all PPE+ neurons also expressed FoxP2, and vice versa), and only populations comprising ≥ 1% of GPe neurons are included. One-fifth of GPe neurons co-express PPE and FoxP2, while another quarter of GPe neurons (“HuCD only”) do not express PPE, FoxP2, or PV. (D) Nkx2-1+ GPe neurons are numerous; some lack PV (arrows). FoxP2+ neurons (arrowheads) do not express Nkx2-1. (E) Expression profiles for Nkx2-1, FoxP2, and PV. (F) Most Nkx2-1+ neurons co-express PV. A minority of HuCD+ neurons express PV, but not any other tested marker (“PV only”). (G) Representative example (single animal) of locations of neurons expressing different molecular markers at three rostro-caudal levels of GPe. Color code as in (F). For clarity, prototypic neurons (Nkx2-1+/PV+ and Nkx2-1+/PV−, left) are separated from arkypallidal (FoxP2+) and other GPe neurons (right). D, dorsal; L, lateral. Scale bars in (A) and (D), 20 μm; (G), 200 μm.

Arkypallidal neurons express the neuropeptide precursor preproenkephalin (PPE), whereas prototypic neurons do not; however, many prototypic neurons express the calcium-binding protein parvalbumin (PV) (Mallet et al., 2012). As a first step toward characterizing the development and functions of arkypallidal and prototypic neurons in the dopamine-intact brain, we sought to define more comprehensive sets of molecular markers with which to discriminate the two cell types unambiguously. Using unbiased, stereological cell counting in adult wild-type mice (see Figure S1), we determined that PPE-expressing (PPE+) GPe neurons also expressed the transcription factor forkhead box protein P2 (FoxP2), and vice versa, and that PPE+/FoxP2+ arkypallidal neurons constitute 20% of all GPe neurons (Figures 1A–1C). The majority of GPe neurons in the rodent and human brain express a different transcription factor, NK2 homeobox 1 (Nkx2-1), and most Nkx2-1+ GPe neurons also express PV (Flandin et al., 2010; Magno et al., 2009, 2011; Nóbrega-Pereira et al., 2010). As such, we reasoned that Nkx2-1 expression in the GPe might be selective for prototypic neurons, irrespective of PV expression. We determined that Nkx2-1+ neurons constitute 68% of all GPe neurons, and that two-thirds of Nkx2-1+ neurons co-expressed PV (Figures 1D–1F). Importantly, however, co-expression of Nkx2-1 and FoxP2 was negligible (Figures 1D–1F). These data suggest that Nkx2-1 is a highly selective marker of prototypic neurons, the most numerous GABAergic cell type in the mouse GPe. Consistent with the mapping of a small number of identified arkypallidal and prototypic neurons in the rat (Mallet et al., 2012), FoxP2+ neurons and Nkx2-1+ neurons were not confined to a particular region of GPe, but were instead intermingled throughout this nucleus (Figure 1G). Previous work in anesthetized, Parkinsonian rats has shown that the spontaneous firing rates of arkypallidal neurons tend to be much lower than those of prototypic GPe neurons during cortical slow-wave activity (Mallet et al., 2012). To test whether the same physiological distinction applies in the dopamine-intact mouse GPe, we extracellularly recorded and juxtacellularly labeled single GPe neurons in anesthetized adult mice (see Figure S2). We determined that, during cortical slow-wave activity, Nkx2-1+ prototypic GPe neurons tended to fire at high rates (∼22 spikes/s; Figure S2A). In contrast, FoxP2+ arkypallidal neurons fired at significantly lower rates (∼4 spikes/s; p < 0.01, t test; Figures S2B and S2C). On the basis of these molecular profiling experiments and electrophysiological recordings, we conclude that arkypallidal and prototypic neurons can be defined by their mutually exclusive expression of FoxP2 and Nkx2-1, respectively.

**Figure 2:**
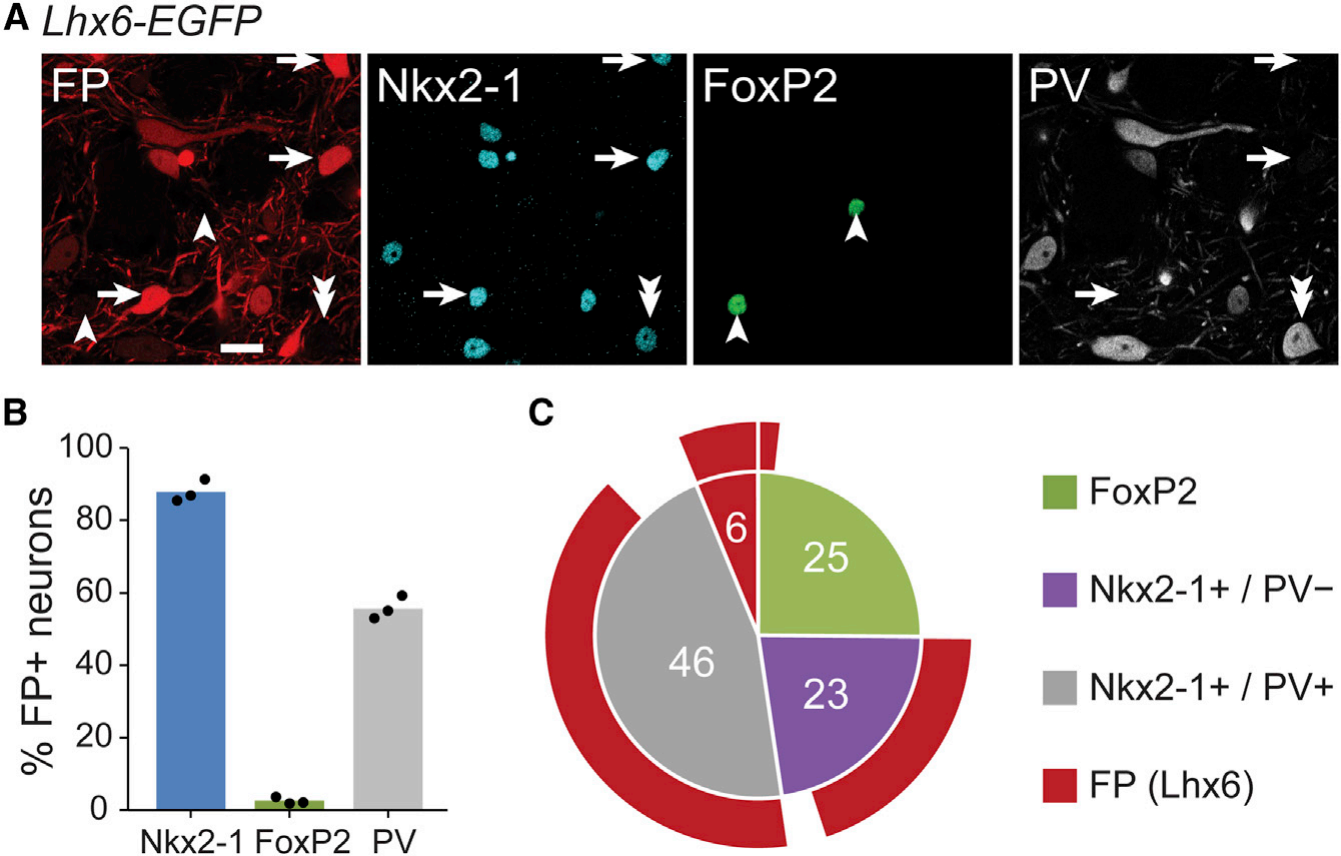
The Majority of Prototypic GPe Neurons Co-Express Lhx6 (A) Immunofluorescence labeling of GPe neurons in an *Lhx6-EGFP* mouse (FP, fluorescent protein; pseudocolored red). Most FP+ neurons co-expressed Nkx2-1 (arrows), but not FoxP2 (arrowheads). Some Nkx2-1+ neurons did not express FP (double arrowhead). (B) Expression profiles of FP+ neurons. (C) Proportions of GPe neurons expressing FP alone, Nkx2-1 (with or without PV), or FoxP2. Note that many PV+ and PV− prototypic neurons were also FP+. Scale bar, 20 μm.

To further explore the molecular diversity of GPe neurons, we extended our analyses to account for LIM homeobox 6 (Lhx6), which acts downstream of *Nkx2-1* to direct the migration and specification of several telencephalic cell types, including some GPe neurons (Du et al., 2008; Flandin et al., 2010; Liodis et al., 2007; Sussel et al., 1999; Zhao et al., 2008), although its roles in GPe are relatively subtle and can be partly fulfilled by other transcription factors (Flandin et al., 2011; Zhao et al., 2008). We examined EGFP immunoreactivity in *Lhx6-EGFP* mice as a proxy for expression of this transcription factor (Flandin et al., 2010). We determined that the vast majority (88%) of EGFP+ GPe neurons were also Nkx2-1+, and vice versa (Figure 2). The proportions of PV+ or PV− prototypic (Nkx2-1+) neurons that co-expressed EGFP were similar (87% and 88%, respectively). However, immunofluorescence signals for EGFP tended to be highest in PV− neurons (Figure 2A, white arrows), suggesting that Lhx6 expression might be lower in PV+ neurons. These data imply that Lhx6 is an additional marker of prototypic neurons in the adult GPe. In agreement with this, very few (< 3%) EGFP+ neurons also expressed FoxP2 (Figures 2A–2C).

We also analyzed the expression of neuronal PAS domain protein 1 (Npas1), a transcription factor which marks some Nkx2-1+ neurons and a distinct set of Nkx2-1− neurons in GPe (Flandin et al., 2010; Nóbrega-Pereira et al., 2010). We observed that one-third of adult GPe neurons express Npas1 (Figure S3). Co-expression of FoxP2 and Npas1 was common; most (87%) FoxP2+ arkypallidal neurons co-expressed Npas1, and many (56%) Npas1+ neurons co-expressed FoxP2 (Figure S3). A minority (12%) of Nkx2-1+ neurons co-expressed Npas1 (most of these prototypic neurons were PV−; Figure S3). These data show that, although the majority (85%) of Nkx2-1−/Npas1+ GPe cells are arkypallidal neurons, Npas1 expression alone does not neatly discriminate arkypallidal from prototypic neurons.

### Arkypallidal and Prototypic GPe Neurons Arise from Distinct Developmental Lineages

**Figure 3:**
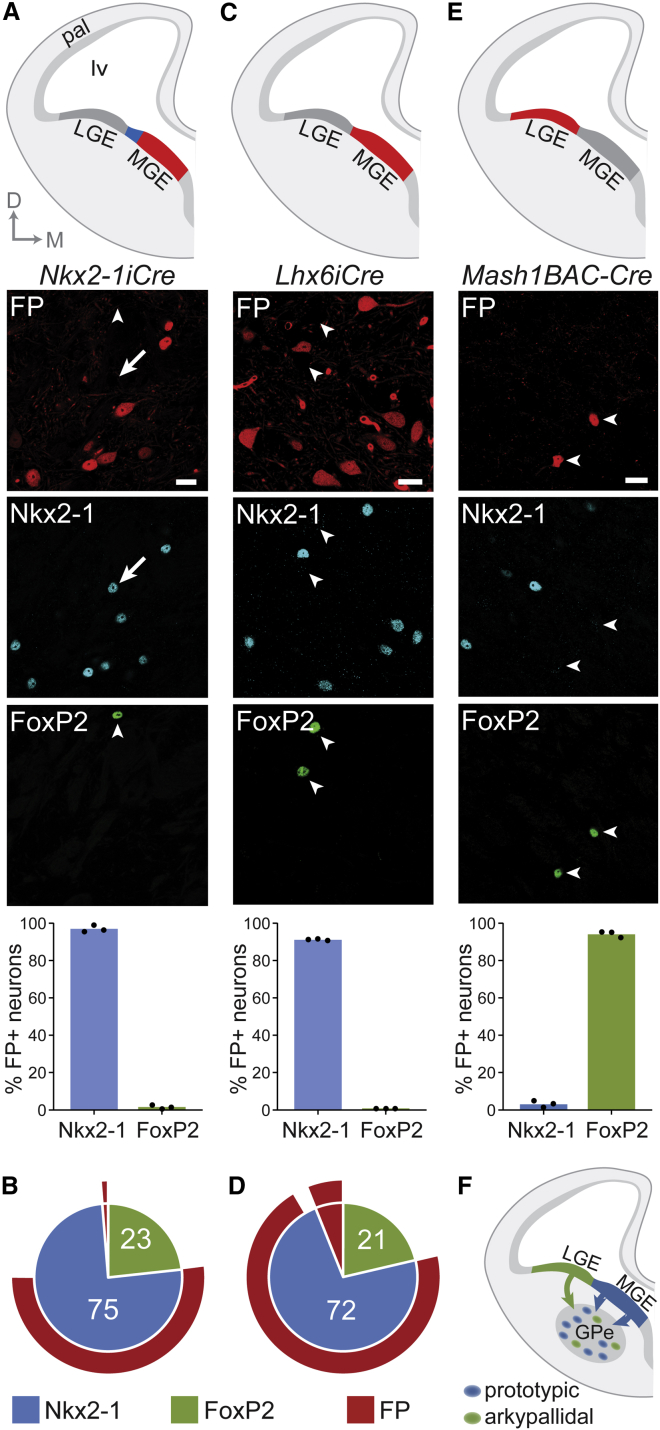
Arkypallidal and Prototypic GPe Neurons Have Distinct Embryonic Lineages (A, C, and E) Immunofluorescence labeling and marker expression profiles in three lines of mice used for fate mapping of GPe neurons (FP, fluorescent protein; pseudocolored red). These mice report neurons derived from different embryonic progenitor domains; the regions of the medial ganglionic eminence (MGE) or lateral/caudal ganglionic eminences (LGE/CGE) reported by each mouse line are highlighted in red in the top schematics of embryonic brain. D, dorsal; M, medial; pal, pallium; lv, lateral ventricle. (A and B) In *Nkx2-1iCre;Z/EG* mice, which report most MGE-derived neurons, 97% FP+ neurons co-express Nkx2-1. However, only 68% of Nkx2-1+ neurons were also FP+ (B). Thus, a substantial proportion of Nkx2-1+ neurons are not captured by this mouse line (also see arrow in A), likely those emanating from the dorsal-most region of MGE (blue region in top schematic). FP+ neurons in these mice did not co-express FoxP2 (arrowhead). (C and D) In *Lhx6iCre;RCE* mice, which report nearly all MGE-derived neurons, the vast majority of FP+ neurons expressed Nkx2-1, and vice versa. FP+ neurons in these mice did not co-express FoxP2 (arrowheads). (E) In *Mash1BAC-CreER;RCE* mice, which report neurons derived from LGE/CGE, the vast majority of FP+ neurons co-expressed FoxP2 (arrowheads), but not Nkx2-1. (F) Schematic summary of fate-mapping experiments. Arkypallidal (FoxP2+) neurons derive from the LGE/CGE of the embryonic subpallium, whereas prototypic (Nkx2-1+) neurons derive from the MGE. Scale bars, 20 μm.

The use of transcription factor expression to classify arkypallidal and prototypic neurons has relevance for understanding the developmental origins of these GPe cell types. Indeed, *Nkx2-1* is expressed throughout the proliferative ventricular zone of the MGE in the embryonic subpallium/ventral telencephalon (Butt et al., 2005; Flames et al., 2007; Long et al., 2009; Nóbrega-Pereira et al., 2010; Sussel et al., 1999), suggesting that the MGE is the progenitor domain of origin of most GPe neurons (Flandin et al., 2010; Nóbrega-Pereira et al., 2010; Sussel et al., 1999). However, as much as one quarter of all GPe cells are generated outside the Nkx2-1+ MGE (Nóbrega-Pereira et al., 2010). The origin(s) of this second population of GPe neurons is not well defined, but the lateral ganglionic eminence (LGE) is a reasonable candidate (Nóbrega-Pereira et al., 2010). Given that mature arkypallidal neurons make up almost one quarter of all GPe cells and do not express Nkx2-1, we hypothesized that their developmental lineage is distinct from that of prototypic GPe neurons. To test this, while also accounting for the possibility that arkypallidal neurons might originate from the MGE, but then downregulate Nkx2-1 expression during their development, we used a recombinase-based genetic fate-mapping approach (Jensen and Dymecki, 2014). We first examined the GPe of adult *Nkx2-1iCre;Z/EG* mice (Anastasiades and Butt, 2011; Kessaris et al., 2006) that are designed to indelibly label (with fluorescent protein) all neurons that expressed *Nkx2-1* at any time during their development. We determined that only a tiny fraction (1.5%) of EGFP+ neurons also expressed FoxP2 (Figures 3A and 3B), consistent with our finding in wild-type mice that adult arkypallidal neurons do not express Nkx2-1. Almost all EGFP+ neurons co-expressed Nkx2-1 (Figure 3A), suggesting that few GPe neurons downregulate Nkx2-1 expression during development. However, about one-third of Nkx2-1+ GPe neurons did not express EGFP (Figures 3A and 3B). Although we cannot rule out the possibility that the *Z/EG* reporter gene was not active in this subset of prototypic neurons, this discrepancy likely arose because these *Nkx2-1iCre;Z/EG* mice do not capture Nkx2-1+ progenitors in the most dorsal, sulcal region of MGE (Fogarty et al., 2007), leaving open the possibility that arkypallidal neurons might still be generated from progenitors in this restricted region. To test whether this was the case, we carried out a second fate-mapping study using *Lhx6iCre;RCE* mice that capture and label with fluorescent protein virtually all MGE-derived neurons (Fogarty et al., 2007; Miyoshi et al., 2010). Consistent with our observations in *Lhx6-EGFP* mice (Figure 2), there was a high overlap of EGFP and Nkx2-1 expression in GPe neurons in the *Lhx6iCre;RCE* mice (91% of EGFP+ neurons were Nkx2-1+, and 96% of Nkx2-1+ neurons were EGFP+; Figures 3C and 3D). Thus, prototypic GPe neurons likely derive from the MGE. In agreement with this, and as could be expected (Figure 2), two-thirds of the EGFP+ GPe neurons in *Lhx6iCre;RCE* mice also expressed PV. Importantly though, the proportion of EGFP+ neurons co-expressing the arkypallidal neuron marker FoxP2 was negligible (< 1%) (Figures 3C and 3D). In summary, these two fate-mapping experiments concurrently indicate that arkypallidal neurons are not derived from the MGE (nor from any other cells expressing *Nkx2-1* or *Lhx6*), and thus, that their developmental origin is distinct from that of prototypic neurons.

Within the embryonic subpallium, FoxP2 expression is prominent in the mantle and subventricular zones of the LGE, less abundant in the caudal ganglionic eminence (CGE), and relatively scant in MGE (Ferland et al., 2003; Long et al., 2009; Takahashi et al., 2003). It is thus plausible that arkypallidal neurons are derived from the LGE and/or CGE. To test this hypothesis, we performed a third fate-mapping experiment using *Mash1BAC-CreER;RCE* mice (Miyoshi et al., 2010). In this particular *Mash1BAC-CreER* driver mouse line, a tamoxifen-inducible form of Cre recombinase is expressed under the control of the *cis*-regulatory elements of the transcription factor *Mash1* (*Ascl1*), but, by chance, is almost exclusively restricted to Mash1-expressing cells in the LGE and CGE (Miyoshi et al., 2010). Following embryonic day (E)12.5 tamoxifen administration, we observed that only a small proportion (3%) of EGFP+ neurons in the adult GPe expressed Nkx2-1 (Figure 3E), thus confirming that this genetics-based strategy does not label neurons derived from the MGE at the peak point of neurogenesis from this eminence (Miyoshi et al., 2010). In contrast, the vast majority (94%) of EGFP+ neurons co-expressed FoxP2 (Figure 3E). This key observation also held true following tamoxifen administration at E10.5 or E13.5 (Figure S4A). The proportion of FoxP2+ neurons co-expressing EGFP after tamoxifen administration at E12.5 or E13.5 was greater than that after administration at E10.5 (Figure S4B), consistent with most GPe neurons being generated after E10.5 (Nóbrega-Pereira et al., 2010). To further test the hypothesis that arkypallidal neurons are derived from LGE/CGE, we performed a fourth fate-mapping experiment using neonatal *Lhx6iCre;Ai9;Dlx1-Venus*^fl^ mice (Rubin et al., 2010) that can simultaneously report on MGE-derived neurons (through Cre-mediated activation of tdTomato fluorescent protein) and LGE/CGE-derived neurons (by continued expression of a “floxed” Venus fluorescent protein). We observed that < 1% of FoxP2+ GPe neurons co-expressed tdTomato, and vice versa (Figure S5), which supports our analyses in other mouse lines showing that arkypallidal neurons are not derived from the MGE (Figure 3). We also observed that almost all (96%) Venus+ GPe neurons co-expressed FoxP2, and that most (89%) FoxP2+ GPe neurons co-expressed Venus (Figure S5). Taking the results of our four complementary fate-mapping experiments together, we conclude that prototypic GPe neurons are derived from the MGE, whereas arkypallidal neurons are derived from the LGE/CGE (Figure 3F).

### Arkypallidal and Prototypic GPe Neurons Have Distinct Firing Properties in Awake Mice at Rest

**Figure 4:**
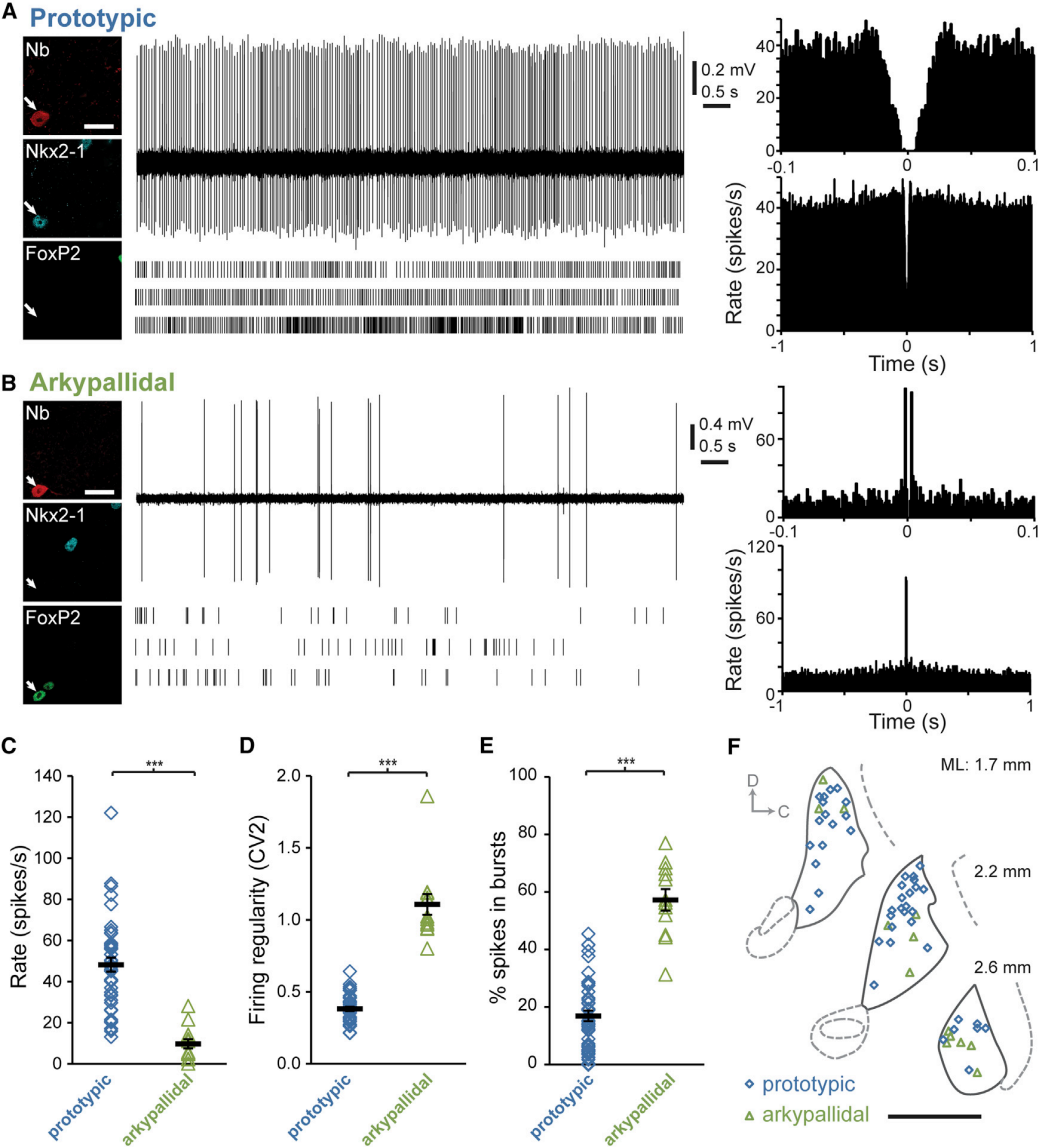
Firing Properties of Identified GPe Neurons in Awake Mice at Rest (A and B) Typical single-unit activity (top), extended raster plots (bottom), autocorrelograms (right), and expression profiles (far left; scale bars, 20 μm) of a prototypic neuron (A) and an arkypallidal neuron (B). Individual neurons were juxtacellularly labeled with Neurobiotin (Nb) after recording; prototypic neurons expressed Nkx2-1 (but not FoxP2), whereas arkypallidal neurons expressed FoxP2 (but not Nkx2-1). (C) Mean firing rate of prototypic neurons was significantly higher than that of arkypallidal neurons (48.3 ± 3.4 versus 9.8 ± 2.3 spikes/s; n = 44 and 13 neurons, respectively). (D) Firing of prototypic neurons was more regular than that of arkypallidal neurons (CV2 of 0.38 ± 0.02 versus 1.11 ± 0.07). (E) Prototypic neurons fired fewer spikes within bursts as compared to arkypallidal neurons (16.9% ± 1.8% versus 57.3% ± 3.7% of spikes; n = 44 and 12 neurons, respectively). (F) Schematic parasagittal sections (D, dorsal; C, caudal) denoting the locations within the GPe of all recorded and identified neurons. Mediolateral (ML) distance from Bregma is shown on right. Scale bar, 1 mm. Data are represented as mean ± SEM; ^∗∗∗^p < 0.001.

Having established that arkypallidal and prototypic GPe neurons have distinct embryonic origins and express different sets of transcription factors, we sought to define whether and to what extent such diversity translates to the functional roles played by these two cell types in the adult brain. To achieve this, we extracellularly recorded the unperturbed spike firing of individual GPe neurons in awake, head-fixed wild-type mice. After recording, each GPe neuron included in this study was juxtacellularly labeled with Neurobiotin (Mallet et al., 2012), and then tested post hoc for FoxP2 and Nkx2-1 immunoreactivity; FoxP2+/Nkx2-1− GPe neurons were designated as arkypallidal neurons, whereas Nkx2-1+/FoxP2− neurons were identified as prototypic neurons. The mice were habituated to head fixation, but were not trained in any task. They were supported by a foam wheel during recordings; they typically rested on the wheel, but occasionally ran or made brief movements and postural adjustments (see below). In comparing the firing properties of identified arkypallidal and prototypic neurons, we first isolated electrophysiological data epochs recorded when the mice were at rest (Figure 4). During this behavioral state of alert immobility, identified prototypic neurons fired at high rates (∼50 spikes/s), and in a tonic manner with occasional brief bursts and pauses (Figures 4A and 4C). This activity profile is similar to that of the majority of GPe neurons (of unknown cell type) recorded in dopamine-intact awake rats, cats, and primates (Anderson and Turner, 1991; Benhamou et al., 2012; Boraud et al., 1998; DeLong, 1971; Elias et al., 2007; Jaeger et al., 1995; Sachdev et al., 1989). In stark contrast, identified arkypallidal neurons fired sporadically and at relatively low rates (∼10 spikes/s; Figures 4B and 4C). Compared to prototypic neurons, arkypallidal neurons had a significantly lower firing rate (p < 0.001, Mann-Whitney; Figure 4C), and fired less regularly (quantified with CV2; p < 0.001, Mann-Whitney; Figure 4D) with more bursts (p < 0.001, t test; Figure 4E). Because we recorded prototypic and arkypallidal neurons throughout the GPe (Figure 4F), these differences were not likely a consequence of biased sampling in a specialized neural subcircuit. These data suggest that, during alert rest, the collective activities (and thus, outputs) of arkypallidal and prototypic GPe neurons are profoundly different.

### Firing of Arkypallidal and Prototypic GPe Neurons Is Different during Spontaneous Movement

**Figure 5:**
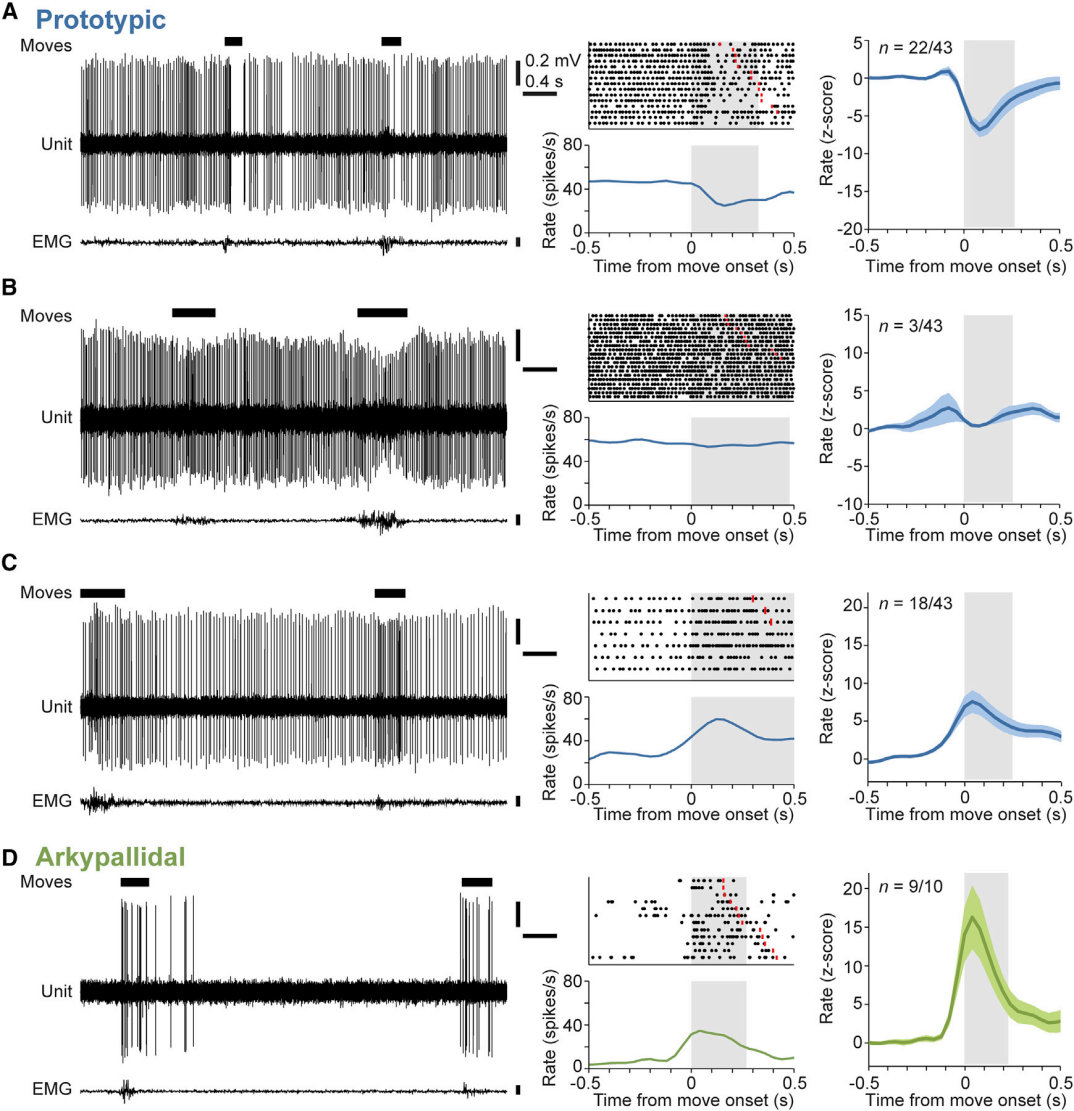
Activity Changes of Arkypallidal and Prototypic GPe Neurons Are Different during Spontaneous Movement (A–D) Example single-unit activity (left), electromyograms (EMG, lower traces), and individual peri-event time histograms (PETH) and raster plots for three different prototypic (Nkx2-1+/PV+) GPe neurons (A)–(C) and an arkypallidal (FoxP2+) neuron (D) during brief spontaneous movements (denoted by black bars). Prototypic neurons (n = 43) could be subdivided into three groups based on the polarity of their firing rate changes during movement (mean, normalized PETH ± SEM shown for each group, right). (A) During movement, 22 of 43 prototypic neurons significantly decreased their firing rate. (B) During movement, three prototypic neurons showed no significant change. (C) During movement, 18 prototypic neurons significantly increased their rate. (D) In contrast, arkypallidal neurons (n = 10) uniformly increased their firing rates during movement (nine showed significant increases). Mean movement duration is denoted by gray shading. In raster plots, the end of individual movement epochs is indicated by red lines.

**Figure 6:**
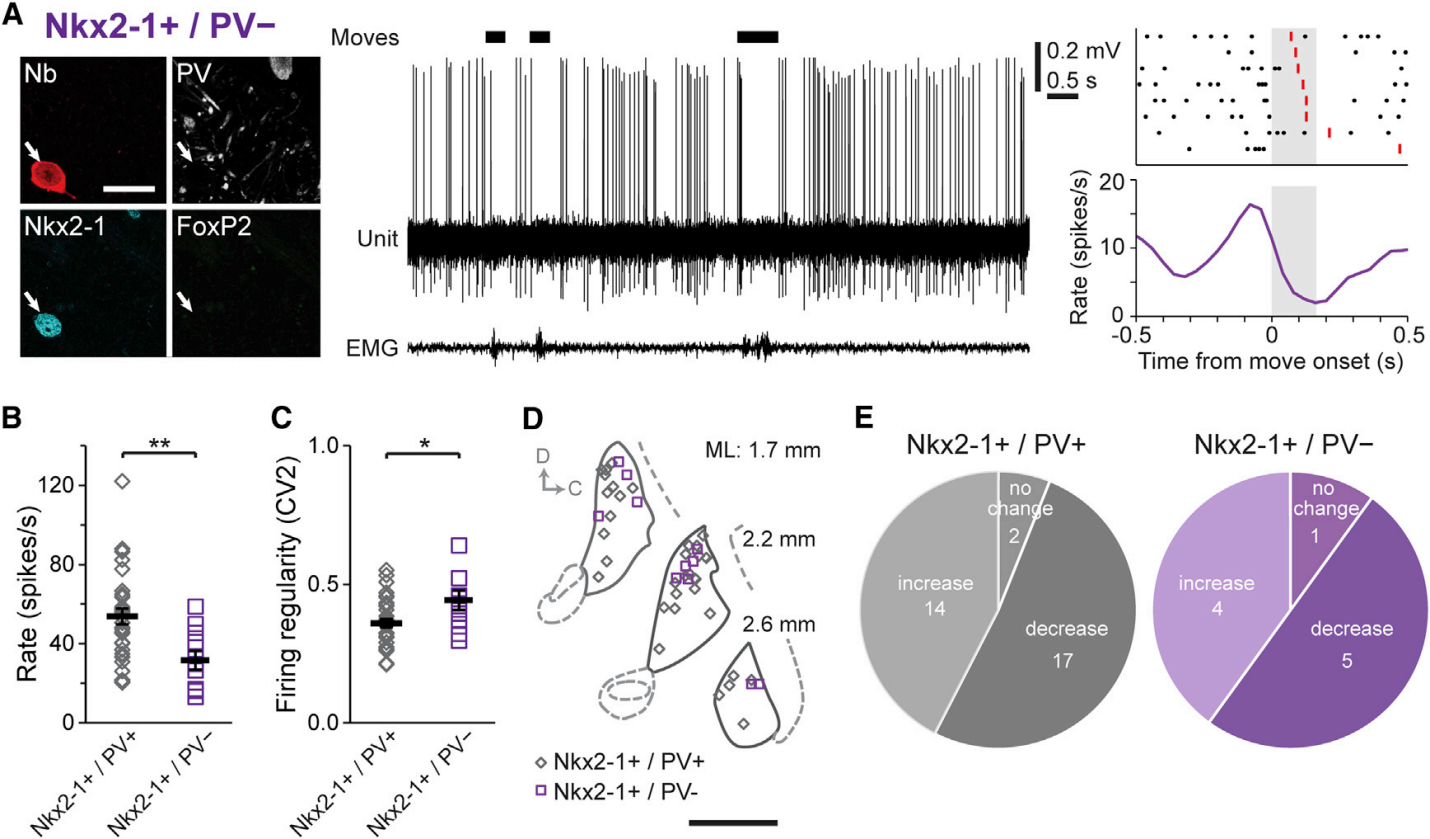
Differential Expression of Parvalbumin by Prototypic Neurons Does Not Account for Their Heterogeneous Firing during Movement (A) Single-unit activity (with corresponding EMG and movement epochs) and PETH (with corresponding raster plot) of a prototypic (Nkx2-1+) neuron that did not express parvalbumin (PV). (B and C) Nkx2-1+/PV+ prototypic neurons fired significantly faster than Nkx2-1+/PV− neurons (53.83 ± 3.9 versus 31.7 ± 4.7 spikes/s, respectively), and more regularly than Nkx2-1+/PV− neurons (CV2 of 0.36 ± 0.02 versus 0.44 ± 0.03). (D) Schematic parasagittal sections (D, dorsal; C, caudal) denoting the locations within the GPe of all recorded PV+ and PV− prototypic neurons (n = 33 and 11 neurons, respectively). (E) Proportions of response type (firing decrease, increase, or no significant change) during movement were similar for Nkx2-1+/PV+ neurons (left) and Nkx2-1+/PV− neurons (right). Data are represented as mean ± SEM. ^∗∗^p < 0.01, ^∗^p < 0.05.

We next tested whether the differences between arkypallidal and prototypic GPe neurons extended to their representation of spontaneous voluntary movements. The movement-related firing of GPe neurons has been most extensively studied in primates, and has been shown to be dependent upon several factors including the body region involved, the kinematic features of the movement, the potential outcome of moving (e.g., related to “reward prediction”), and whether a movement is passive, active, or externally cued (Anderson and Turner, 1991; Arkadir et al., 2004; DeLong et al., 1985; Georgopoulos et al., 1983; Hamada et al., 1990; Mitchell et al., 1987; Turner and Anderson, 2005). This complexity in neural dynamics, coupled with the challenge of post hoc identification of the recorded cell types, necessitated that we sample a small array of behaviors that were not biased to a particular body part, kinematic parameter, or experimenter-derived factor. We therefore focused our analyses on the GPe neuron activity recorded around brief (< 1 s) spontaneous movements, which typically involved the mouse altering whole-body posture to adjust its position on the wheel. We reasoned that, despite the heterogeneous kinematics of these self-paced voluntary movements, any consistent neuronal responses that emerged would reflect general organizational or coding principles of GPe cell types. In support of this, we observed that many GPe neurons robustly changed their firing during each defined movement period (Figure 5). To quantify and compare changes in firing across different sets of neurons, we first normalized firing rates as *Z* scores; the majority of prototypic neurons (40/43) exhibited significant firing rate changes during movement (with significance defined as more than two SDs from baseline, i.e., a *Z* score of ± 2). Just over half (22/40) of the “movement-responsive” prototypic neurons decreased their firing rate, while the remaining 18 prototypic neurons increased their rate. Despite these differences between prototypic neurons, the response “polarity” of a given neuron (i.e., a decrease or increase in rate) was consistent from movement to movement (Figures 5A–5C). In contrast to the heterogeneous responses of prototypic neurons, all responsive arkypallidal neurons exhibited robust increases in their firing rate during movement (Figure 5D). Irrespective of response polarity, changes in the firing rates of both cell types evolved rapidly (with peak responses occurring within 100–150 ms of movement onset) (Figure 5). The peak decrease in firing rate of prototypic neurons occurred at a significantly longer latency than that of the peak increase in arkypallidal neuron firing (p < 0.01, Mann-Whitney). These differences in the polarities, timing, and relative heterogeneity of responses of prototypic and arkypallidal neurons did not arise from any systematic differences in the movements recorded with each cell type. Indeed, the average durations of movement periods recorded with prototypic or arkypallidal neurons were similar (p > 0.05, t test). However, because almost one-third of prototypic neurons do not express PV (Figure 1F), we considered whether the heterogeneity of responses in prototypic neurons during movement mapped onto this molecular diversity. To address this, we divided prototypic neurons according to their expression of PV (Figure 6). We first compared the firing of PV+ and PV− prototypic neurons when the mice were at rest. PV-expressing neurons fired significantly faster (p < 0.01, t test; Figure 6B) and more regularly (p < 0.05, Mann-Whitney; Figure 6C) than PV− neurons, but both groups of prototypic neurons fired at higher rates and more regularly than arkypallidal neurons. We next tested whether PV+ and PV− prototypic neurons differed in their responses during movement. Both groups of prototypic neurons showed similar proportions of each response type (p > 0.05, Fisher’s exact test; Figure 6E). This analysis suggests that selective expression of PV does not correlate with the heterogeneous responses of prototypic GPe neurons during movement.

### Arkypallidal and Prototypic GPe Neurons Can Reliably Encode Movement with Changes in Firing Rate

**Figure 7:**
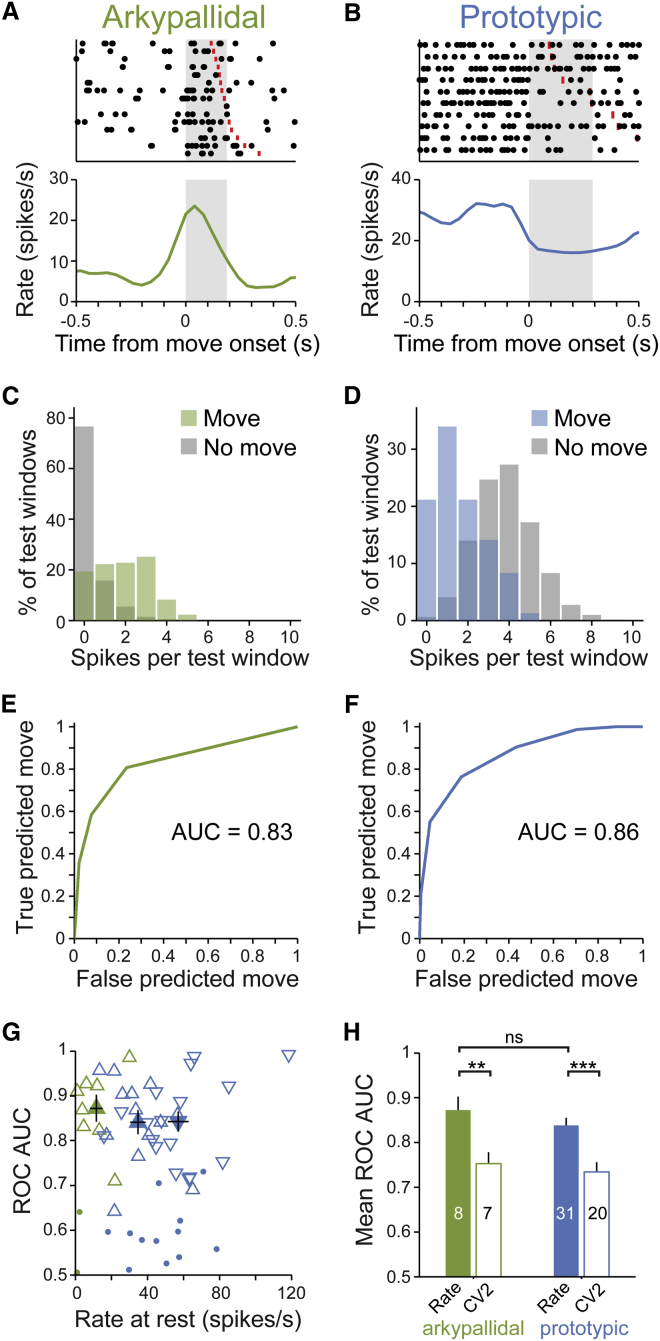
Both Arkypallidal and Prototypic Neurons Reliably Encode Movement The ability of arkypallidal neurons and prototypic neurons to encode movement was assessed using receiver operating characteristic (ROC) analysis. (A and B) Example PETH and raster plot for a single neuron of each cell type. (C and D) Histograms for the same two neurons, showing the number of spikes in each test window classified as movement or non-movement. (E and F) ROC curves for the same two neurons, showing the proportion of windows containing genuine movement and classified as movement from the spike train (a true prediction) versus the fraction of windows without movement and falsely classified as movement (false prediction) for each threshold value. An area under the ROC curve (AUC) of 0.5 corresponds to chance classification, while an AUC of 1 corresponds to perfect discrimination of movement. (G) AUC plotted against firing rate during alert rest for all GPe neurons (arkypallidal in green, prototypic in blue). Filled circles represent individual neurons for which the AUC was not significantly different from shuffled data. Open triangles represent individual neurons that significantly encoded movement (▵ = increased rate; ▿ = decreased rate). Mean values for significantly encoding neurons of each group are indicated by filled triangles. (H) Mean AUCs for the arkypallidal (0.87 ± 0.03 for rate, 0.75 ± 0.03 for CV2) and prototypic neurons (0.84 ± 0.02 for rate, 0.73 ± 0.02 for CV2) able to significantly discriminate movement using firing rate or CV2 as a classifier (numbers of discriminating neurons are indicated within bars; ^∗∗^p < 0.01; ^∗∗∗^p < 0.001; ns, not significantly different). Data are represented as mean ± SEM.

Although arkypallidal and prototypic neurons differed in their average response polarities, both cell types appeared to encode spontaneous movements with changes in their firing rates. To address this quantitatively, we tested whether movements could be decoded (i.e., predicted) from the spike trains of the individual neurons. More specifically, we computed receiver operating characteristic (ROC) curves for each GPe neuron to test whether spike rate could correctly classify the occurrence of a movement (Figures 7A–7F). We determined that most arkypallidal and prototypic neurons were indeed able to encode movement with their firing rate (Figure 7G). To ensure that neurons were not simply performing at chance levels, we compared the area under the ROC curves (AUC) for each neuron to confidence intervals obtained by shuffling the corresponding movement periods; most (8/10) arkypallidal neurons and most (31/43) prototypic neurons predicted movement significantly above chance (Figures 7G and 7H). The average AUCs of arkypallidal and prototypic neurons predicting movement were not significantly different (Figure 7H), suggesting that, despite clear differences in response polarities and heterogeneity, both cell types encode movement in their firing rates to a similar level of accuracy. Among the prototypic neurons encoding movement, those that decreased their firing rate during movement had higher average firing rates at rest than those that increased their rate during movement (57.1 ± 5.7 and 34.8 ± 4.3 spikes/s, respectively, at rest; p < 0.01, t test; Figure 7G).

Classical schemes of basal ganglia function and dysfunction are essentially “rate models” in that they posit that changes in neuron firing rates are of special significance for the encoding and governance of voluntary action (DeLong, 1990; Wichmann and DeLong, 1996). However, studies of human movement disorders (particularly Parkinson’s disease) and their animal models emphasize that the temporal patterning of basal ganglia activity is also key (Bevan et al., 2002; Hammond et al., 2007; Nambu, 2008). With this in mind, we tested whether the movements we studied were better encoded by the rate or pattern (regularity) of firing of arkypallidal and prototypic neurons in the dopamine-intact GPe. We thus repeated the ROC analysis using a measure of regularity (CV2) as the classifier instead of firing rate. We determined that movement encoding was significantly less accurate when using CV2 as compared to rate for both arkypallidal and prototypic GPe neurons (t tests; Figure 7H). Moreover, fewer neurons performed significantly above chance when using firing regularity to classify movement. This analysis indicates that the firing rates of individual neurons in the dopamine-intact GPe are a more informative representation of movement. A final set of ROC analyses showed that, irrespective of whether firing rates or regularities were considered, PV+ and PV− prototypic neurons did not differ in their abilities to reliably encode movement (see Figure S6). In summary, our recordings of identified arkypallidal and prototypic GPe neurons in awake mice suggest that these two cell types are equally well suited to encoding movement, but, importantly, their accompanying readouts (i.e., the relative polarity and uniformity of their changes in firing rate) are fundamentally different.

## Discussion

Here, we define the developmental origins, molecular architecture, and behavioral significance of arkypallidal and prototypic neurons, the two main cell types of the GPe. We show that they are born in different regions of the embryonic subpallium and express different sets of postmitotic transcription factors. These differences are realized in behavior, with arkypallidal and prototypic neurons differentially encoding spontaneous movement in adult mice. We conclude that the distinct and specialized contributions of these two GPe cell types to voluntary actions are fated from early embryonic life.

Previous work in anesthetized Parkinsonian rats has established the concept of a functional dichotomy in the GPe, as embodied by two major GABAergic cell types that are defined on the basis of their distinct molecular, structural, and physiological properties (Mallet et al., 2012). Our data demonstrate that arkypallidal and prototypic neurons are defined by their mutually exclusive expression of FoxP2 and Nkx2-1, respectively (thus outlining a transcriptional “blueprint” for these cell types), establish that arkypallidal neurons constitute one-fifth of all mouse GPe neurons, and show that this functional dichotomy exists in the dopamine-intact brain and extends to other species. Importantly, our results also help place reports of developmental heterogeneity in GPe neurons (Flandin et al., 2010; Nóbrega-Pereira et al., 2010) in the context of functionally defined cell types in the adult GPe. Thus, arkypallidal and prototypic neurons are born in the LGE/CGE and MGE, respectively, and then, during development, migrate and coalesce to form the GPe proper. Transcriptional codes are key for the generation, specification, migration, and synaptic maturation of neurons, as well as for maintaining the identities of postmitotic neurons (Arber, 2012; Deneris and Hobert, 2014; Jessell, 2000; Kepecs and Fishell, 2014; Siegert et al., 2012). Our data reveal one result of these developmental processes and terminal differentiation, as reflected in the functional organization of adult GPe. Because expression of transcription factors influences the intrinsic membrane properties (and hence, firing), structure, and connectivity of neurons (Butt et al., 2005; Kepecs and Fishell, 2014), it follows that such expression could ultimately govern the specialized roles played by different cell types in behavior. Indeed, the silencing or ablation of specific sets of spinal cord neurons has corroborated this (Arber, 2012; Goulding, 2009; Kiehn, 2011), but it has proven challenging to link transcriptional diversity to the encoding of movement by the spike firing of identified neurons in intact circuits. Here, we provide a unifying experimental framework that combines quantitative molecular profiling with genetic fate mapping and high-resolution sampling of the activity of individual, identified neurons in behaving animals. Using the GPe as an exemplar, we show that two neuron types with different transcriptional provenance play divergent roles in encoding movement on a timescale of milliseconds.

Electrophysiological diversity has long been observed in the GPe. Indeed, some of the first single-unit recordings in the GPe of primates distinguished two major groups of neurons based on their rates and patterns of firing (DeLong, 1971). According to one classification scheme adopted for recordings made in awake rats, monkeys, and Parkinsonian patients, the majority of GPe units are “high-frequency discharge” (HFD) neurons that pause, whereas the remaining ∼15% are “low-frequency discharge bursting” (LFD-B) neurons (Benhamou et al., 2012; Elias et al., 2007; Gardiner and Kitai, 1992; Hutchison et al., 1994). Here, we provide the first definition of the firing of molecularly identified GPe neurons in awake, behaving animals. In doing so, we show that the firing rates and patterns of prototypic GPe neurons in the awake mouse at rest are similar to those of HFD neurons reported in other species. In contrast, the firing rates and patterns of arkypallidal neurons are within the ranges reported for LFD-B neurons, raising the possibility that they are the same cell type. However, although the average firing properties of prototypic and arkypallidal neurons are distinct, there is some overlap, and hence, the ability to correlate behavior-related firing with a molecularly defined cell type is especially valuable.

Prototypic GPe neurons exhibit relatively high firing rates when the animal is at rest and, thus, potentially have a wide dynamic range (large negative and positive activity modulations) for encoding behavior. In contrast, arkypallidal neurons fire at relatively low rates during rest, indicating they have less scope for negative activity modulations. In support of this, almost all prototypic neurons exhibited either decreases or increases in firing rates in time with movements. Moreover, and in contrast, arkypallidal neurons consistently encoded movement with robust increases in firing rate. In primates and rats, movement-related decreases and increases in the firing of GPe HFD neurons are frequently observed, although increases in activity tend to dominate; because the responses of HFD neurons are dependent on many different movement parameters, as well as whether the animal is engaged in a trained and/or rewarded task, we cannot rule out that the balance between increases and decreases in GPe neuron firing rate shifts according to the type of movement studied and a host of other factors (DeLong, 1971; Gardiner and Kitai, 1992; Georgopoulos et al., 1983; Jaeger et al., 1995; Mink and Thach, 1991; Mitchell et al., 1987; Turner and Anderson, 1997). Nevertheless, because we performed our recordings of identified GPe neurons in untrained mice that moved without overt external cueing, our data provide new insights into the cell-type-specific encoding of spontaneous or self-paced movements. It is also possible that the preferred response polarities of prototypic neurons are somewhat fixed in adulthood by genetics. If this were the case, then molecular markers could be exploited in the future to distinguish and functionally interrogate the prototypic neurons that decrease firing versus those that increase firing. However, our data indicate that Nkx2-1, Lhx6, PV, and Npas1 cannot be used for such a stratification.

What are the functional impacts of the changes in firing rates of prototypic and arkypallidal neurons during movement, and how do these changes arise? GABAergic prototypic GPe neurons innervate the STN as well as basal ganglia output nuclei and, occasionally, striatum (Bevan et al., 1998; Kita, 2007; Mallet et al., 2012). A decrease in their firing rate during movement would thus fit well with the proposed role of the GPe in the classical “indirect pathway,” i.e., disinhibition of STN and output nuclei (Kravitz et al., 2010; Sano et al., 2013), which should ultimately inhibit unwanted actions or terminate action sequences (Gerfen and Surmeier, 2011). GABAergic striatopallidal neurons, which are active around movement onset (Cui et al., 2013; Isomura et al., 2013), are prime candidates for mediating the decreases in prototypic neuron firing during movement. Indeed, electrophysiological studies in vitro (Chuhma et al., 2011) and computational work (Nevado-Holgado et al., 2014) indicate that prototypic neurons receive comparatively large striatal inputs and are endowed with robust autonomous firing. The functional roles and circuit substrates for the firing rate increases of a minority of prototypic neurons are less clear, but such increases would be one essential part of a center-surround organization of activity (Mink, 1996; Nambu et al., 2002). Our study provides the first detailed insights into how arkypallidal neurons could potentially contribute to information processing during actions. Arkypallidal neurons do not project to the STN (or to the other “downstream” targets of prototypic neurons), but rather, exclusively and densely innervate the striatum (Mallet et al., 2012). In further contrast to prototypic GPe neurons, arkypallidal neurons are expected to display little firing in the absence of synaptic drive, and also to receive comparatively weak striatal inputs (Chuhma et al., 2011; Nevado-Holgado et al., 2014). As such, the firing of arkypallidal neurons is predicted to be a better reflection of their excitatory inputs from STN (Mallet et al., 2012; Nevado-Holgado et al., 2014). Because arkypallidal neurons are positioned to release GABA and/or enkephalin onto many striatal projection neurons and interneurons (Mallet et al., 2012), and because they have low firing rates at rest and robustly increase activity around movement, they are theoretically suited to facilitate ongoing “action selection,” i.e., by inhibiting large striatal regions, they could prevent competing actions and, thus, help expression of a desired action (Redgrave et al., 1999). The properties of arkypallidal neurons also make them suited to expedite the cessation of actions. The STN plays important roles in stopping actions, rapid switching between tasks, and delaying action onset to aid optimal action selection (Aron and Poldrack, 2006; Frank et al., 2007; Hikosaka and Isoda, 2010; Isoda and Hikosaka, 2008; Schmidt et al., 2013). Many of these roles would benefit from contemporaneous inhibition of striatum. Indeed, computational modeling suggests that the direct action of the STN on basal ganglia output nuclei might be insufficient to cancel a “go signal,” and, thus, additional mechanisms are required to suppress such signals at the level of striatum (Schmidt et al., 2013). The distinct molecular signatures of prototypic and arkypallidal neurons offer tractable access points through which the causative contributions of these cell types to behavior can be defined in the future (using, for example, genetics-based approaches). In conclusion, schemes of the organization of basal ganglia circuits should endeavor to account for the functional dichotomy that is embodied by the arkypallidal and prototypic neurons of the GPe. Our results show that these two cell types are distinguished by their firing during rest and movement, establishing the importance of this dichotomy for normal behavior, and further suggest that this “division of labor” stems from an early prenatal stage of brain development.

## Experimental Procedures

### Animals

All experimental procedures on animals were conducted in accordance with the Animals (Scientific Procedures) Act, 1986 (United Kingdom). Unless noted otherwise, 3- to 4-month-old male mice were used for all experiments. See Supplemental Experimental Procedures for further details.

### Tissue Processing for Light Microscopy

Coronal sections (50 μm) containing the GPe (see Figure S1) were cut from each perfusion-fixed adult mouse brain. Expression of molecular markers by GPe neurons was assessed by indirect immunofluorescence (see Supplemental Experimental Procedures).

### Stereological Sampling

A series of tiled, z-stacked images of immunolabeled neurons was acquired (to a depth of 12 μm from tissue surface) across the entire GPe in both hemispheres at three rostro-caudal levels (Figure S1). Unbiased stereological sampling was used to generate all cell counts from six GPe sections per adult mouse. Cell counts were pooled for each mouse, and then expression profiles were constructed using the mean value from three mice (see Supplemental Experimental Procedures).

### In Vivo Electrophysiological Recording, Juxtacellular Labeling, and Data Analysis

For recordings of electrophysiological signals and behavior, mice were head-fixed using a stainless steel post (surgically implanted above the right pallidum) and positioned above a running wheel. Extracellular recordings of the action potentials (“spikes”) fired by individual GPe neurons were made from 23 wild-type C57Bl6/J mice at rest and/or engaged in brief movements. After recording, each neuron was juxtacellularly labeled with Neurobiotin, recovered after perfuse fixation, and revealed with Cy3-conjugated streptavidin (Mallet et al., 2012). All recorded and identified GPe neurons were tested by indirect immunofluorescence for expression of Nkx2-1, FoxP2, and PV (see Supplemental Experimental Procedures).

Data were acquired and initially analyzed using Spike2 software (Cambridge Electronic Design). Regularity of firing was assessed using mean CV2 (Holt et al., 1996), and bursts were detected using a custom MATLAB (MathWorks) routine based on the Poisson surprise method (see Supplemental Experimental Procedures). To examine movement-related firing of GPe neurons, we focused our analyses on recordings containing ≥ 5 brief posture-adjusting movements (defined as those with < 1-s duration and involving limb movement). Movement onset was determined using a combination of the EMG (measured from cervical muscles) and videos of behavior (30 frames/s). To compare movement-related firing across neurons, peri-event time histograms (40-ms bin width) of GPe neuron activity were transformed to *Z* scores using the baseline mean firing and SD, where baseline was −1,000 to −200 ms relative to movement onset (use of this window avoided possible contamination of baseline with changes in activity just prior to onset). Changes in movement-related activity were considered significant when firing rate crossed a threshold of baseline mean ± 2 SD during the defined movement period. ROC curves were computed using firing rate and CV2 to obtain their distributions with and without movement. True positive was defined as genuine movement that was correctly classified as movement, and false positive was defined as a period of no movement that was incorrectly classified as movement (see Supplemental Experimental Procedures). AUC was considered significant if it was higher than the 95th percentile of AUCs computed from shuffled data.

### Statistical Analyses

Before statistical comparison, a Shapiro-Wilk test was used to judge whether data sets were normally distributed (p < 0.05 to reject). For normally distributed data sets, comparisons were made using a Student’s t test, whereas for all other data, Mann-Whitney rank-sum tests were used (significance p < 0.05; SigmaStat, Systat Software). Data are presented as mean ± SEM throughout.

## Author Contributions

P.D.D. and P.J.M. designed research; P.D.D. performed all electrophysiological experiments and related analyses; P.D.D. and I.C.D. developed recording and juxtacellular labeling in awake mice; P.D.D., J.T.L., J.M.D., F.N.G., N.M.D., N.K., S.J.B.B., and P.J.M. contributed to cell counts and fate-mapping experiments; R.B. and P.D.D. performed ROC analyses; P.J.M. supervised the whole project; P.D.D. and P.J.M. wrote the paper with input from all authors.

## Supplemental Information

Document S1. Figures S1–S6 and Supplemental Experimental Procedures

Document S2. Article plus Supplemental Information
