# Supplementary material for: Distinct Developmental Origins Manifest in the Specialized Encoding of Movement by Adult Neurons of the External Globus Pallidus
Source: Neuron. 2015 Apr 22;86(2):501–13. doi: 10.1016/j.neuron.2015.03.007 (PMC4416107; doi:10.1016/j.neuron.2015.03.007)
Supplement: Document S1. Figures S1–S6 and Supplemental Experimental Procedures [file mmc1.pdf]

Neuron, Volume *86*

Supplemental Information

Distinct Developmental Origins Manifest in the Specialized Encoding of Movement by Adult Neurons of the External Globus Pallidus

Paul D. Dodson, Joseph T. Larvin, James M. Duffell, Farid N. Garas, Natalie M. Doig, Nicoletta Kessaris, Ian C. Duguid, Rafal Bogacz, Simon J.B. Butt, and Peter J. Magill

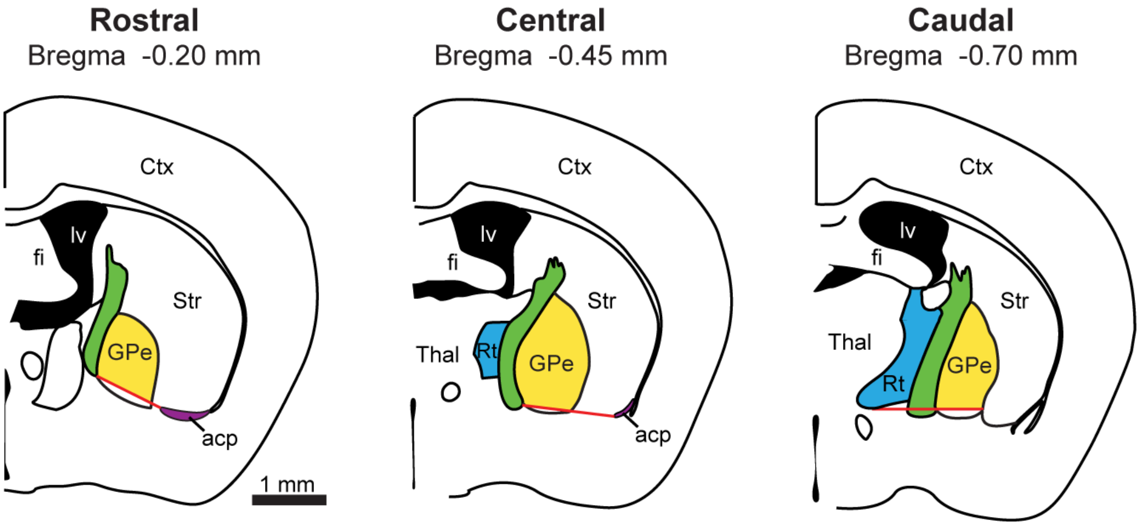

Figure S1 (related to Figures 1–3). Definition of GPe borders used for stereological cell counting. Coronal sections illustrating the rostral, central and caudal levels of the adult mouse external globus pallidus at which the molecular expression profiles of GPe neurons were quantified. For GPe neuron sampling in rostral and central sections, the ventral borders of GPe (red lines) were defined according to the medial edge of the anterior commissure (acp, in purple) and the bottom edge of the internal capsule (green). In caudal sections, the ventral border of GPe was defined according to the reticular nucleus of the thalamus (Rt, in blue). Only those neurons located dorsal to these borders were considered as GPe and counted (yellow). Ctx, cortex; fi, fimbria of the hippocampus; lv, lateral ventricle; Str, dorsal striatum; Thal, anterior thalamus. Schematics adapted from Paxinos and Franklin (2007); a standard stereotaxic reference (approximate distance posterior of Bregma) is given for each rostrocaudal level.

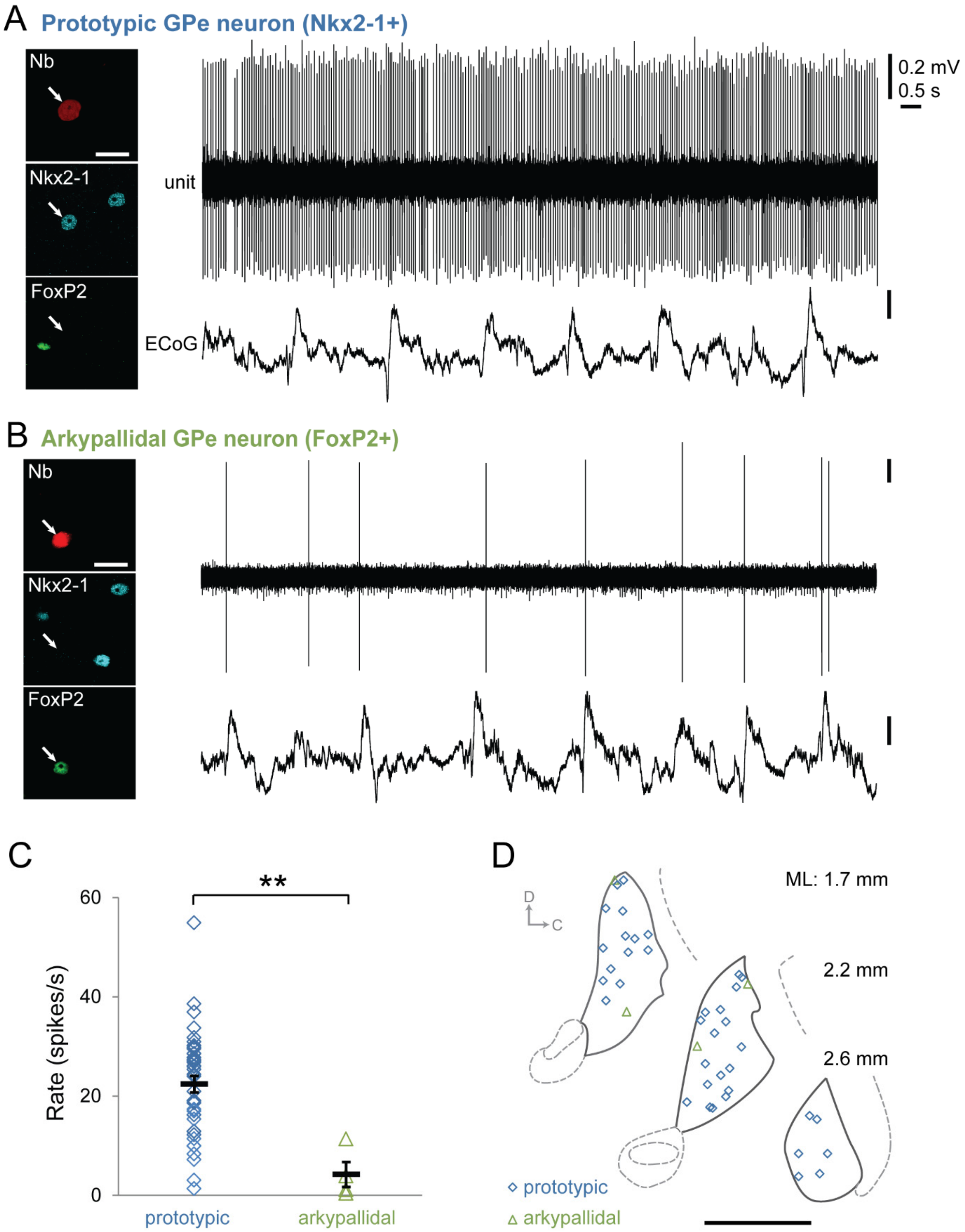
A Prototypic GPe neuron (Nkx2-1+)
Nb
Nkx2-1
FoxP2
unit
ECoG
0.2 mV
0.5 s
B Arkypallidal GPe neuron (FoxP2+)
Nb
Nkx2-1
FoxP2
C
**
60
40
20
0
Rate (spikes/s)
prototypic
arkypallidal
D
D
C
ML: 1.7 mm
2.2 mm
2.6 mm
prototypic
arkypallidal

Figure S2 (related to Figures 1–3). Arkypallidal and prototypic GPe neurons have distinct *in vivo* firing properties in anaesthetized mice.

(A and B) Typical single-unit activity (top) of a prototypic neuron (A) and an arkypallidal neuron (B). All GPe neurons were recorded during robust slow-wave activity, as exemplified by large amplitude, slow (~1 Hz) oscillations in the simultaneously-recorded electrocorticogram (ECoG, bottom). After recording, individual neurons were juxtacellularly labeled with Neurobiotin (Nb); prototypic neurons expressed Nkx2-1 (but not FoxP2) whereas arkypallidal neurons expressed FoxP2 (but not Nkx2-1). Verification of transcription factor expression by the same two example neurons is shown in left panels (scale bars: 20 μm).

C) Mean firing rate of prototypic neurons was significantly higher than that of arkypallidal neurons (22.4 ± 1.65 vs. 4.2 ± 2.5 spikes/s, n = 41 and 4 neurons, respectively; **p < 0.01, t-test). Data are presented as mean ± SEM.

(D) Schematic parasagittal sections (D, dorsal; C, caudal) denoting the locations within the GPe of all recorded and identified neurons. Mediolateral (ML) distance from Bregma is shown right. Scale bar: 1 mm.

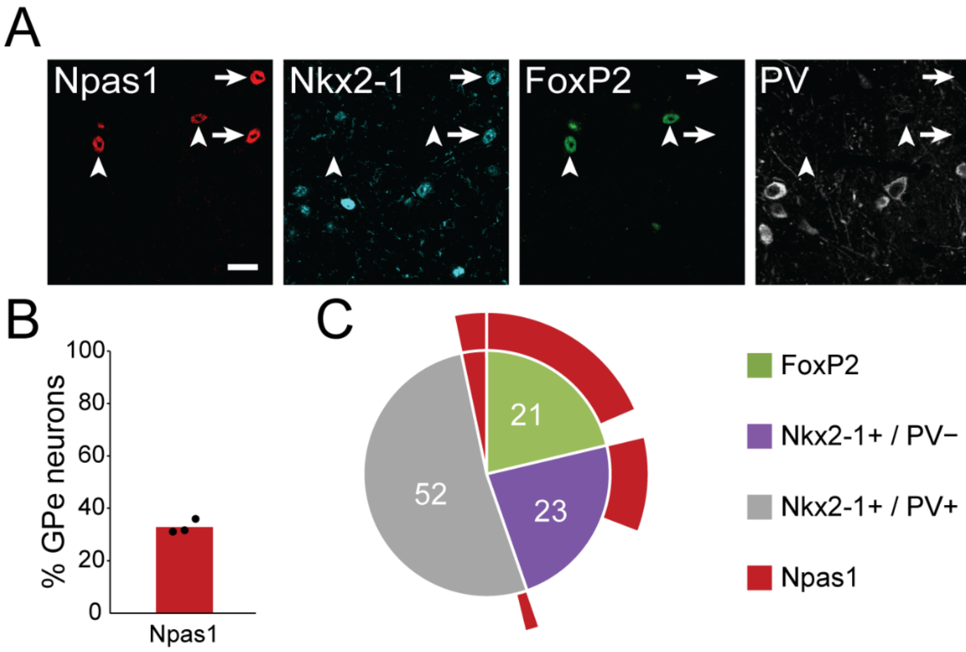

Figure S3 (related to Figures 1 and 2). Npas1 is often expressed by arkypallidal neurons but infrequently expressed by prototypic GPe neurons.

(A) Immunofluorescence labeling of GPe neurons in a wildtype mouse. Most FoxP2+ neurons co-express Npas1 (arrowheads). Some Nkx2-1+/PV− neurons (arrows) also express Npas1.

(B) Proportion of all GPe neurons (*i.e.* all HuCD+ neurons) expressing Npas1. Filled circles represent counts from individual animals.

(C) Proportions of GPe neurons expressing FoxP2, Nkx2-1 (with or without PV), or Npas1 alone. Note that co-expression of FoxP2 and Npas1 is common; the majority (87%) of FoxP2+ arkypallidal neurons co-express Npas1, and the majority of (56%) Npas1+ neurons co-express FoxP2. Also note that Npas1 is co-expressed by a minority (12%) of prototypic neurons (most Nkx2-1+/Npas1+ neurons are PV−). Only populations comprising ≥1% of GPe neurons are included.

Scale bar: 20 µm.

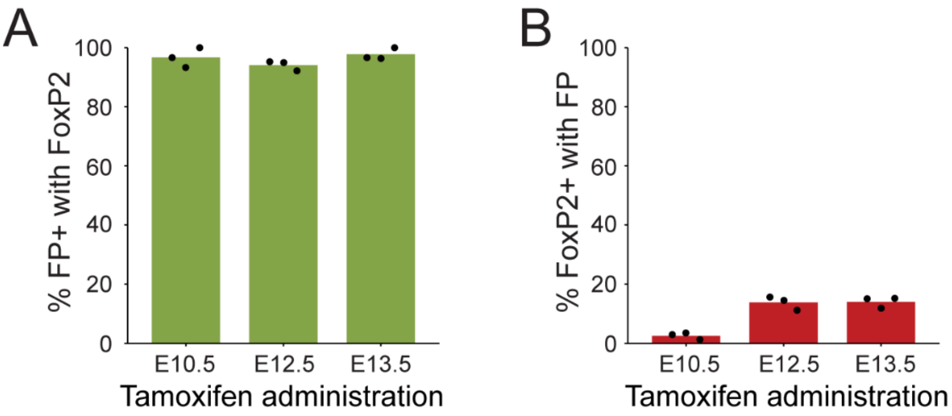

Figure S4 (related to Figure 3). The vast majority of GPe neurons derived from LGE/CGE express FoxP2.

(A) In *Mash1BAC-CreER;RCE* mice, which report neurons derived from LGE/CGE, the vast majority of GPe neurons expressing fluorescent protein (FP+) also expressed FoxP2; this held true across a range of embryonic time points for fate mapping in this mouse line. Tamoxifen was administered to pregnant *Mash1BAC-CreER;RCE* dams at one of three different embryonic time points (E10.5, E12.5 or 13.5), and co-expression of immunoreactivities was quantified in their offspring when they reached adulthood. Filled circles represent counts from individual animals.

(B) The proportion of FoxP2+ neurons co-expressing fluorescent protein in *Mash1BAC-CreER;RCE* mice is dependent upon the embryonic time point of tamoxifen administration. Note that, because of the limited duration and efficiency of tamoxifen-induced Cre activity, and because GPe neurons are generated over several days *in utero* (*i.e.* E10.5 to E13.5), much less than 100% of FoxP2+ neurons are expected to be captured at each time point.

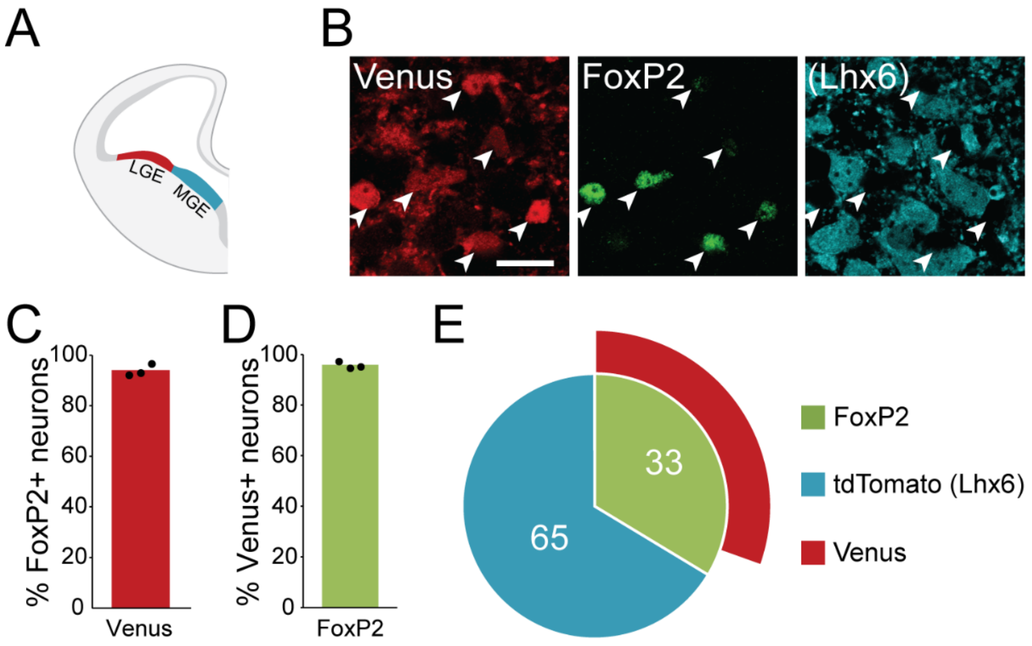

Figure S5 (related to Figure 3). FoxP2+ arkypallidal neurons derive from the LGE/CGE of the embryonic subpallium.

(A) Schematic of embryonic brain illustrating the progenitor domains that are labeled with different fluorescent proteins following a dual fate-mapping strategy using *Lhx6iCre;Ai9;Dlx1-Venus*^{fl} mice. Expression of Venus fluorescent protein (pseudocolored red) is driven from the ATG of the *Dlx1* gene in cells arising from the LGE/CGE and the MGE. The *Dlx1-Venus* transgene is floxed (fl). As such, in cells expressing *Lhx6iCre* (*i.e.* those originating in the MGE progenitor domain), the *Dlx1-Venus* transgene is excised, and the tdTomato fluorescent protein (pseudocolored teal) is instead expressed from the Rosa26 locus. Thus, neurons expressing Venus (but not tdTomato) are derived from the LGE/CGE, whereas tdTomato+ neurons are derived from the MGE. Because expression of the *Dlx1-Venus* transgene is down regulated in GPe neurons during the first postnatal week, cell counts were performed on the first postnatal day (P0.5).

(B) Immunofluorescence labeling of GPe neurons in a P0.5 *Lhx6iCre;Ai9;Dlx1-Venus*^{fl} mouse. The vast majority of Venus+ neurons co-expressed FoxP2, and vice versa (arrowheads); these neurons did not express tdTomato, the proxy marker for cells expressing, or that once expressed, Lhx6.

(C and D) Expression profiles of FoxP2+ neurons and Venus+ neurons. Filled circles represent counts from individual animals.

(E) Proportions of all GPe neurons (*i.e*. all HuCD+ neurons) expressing different markers. Note that the majority (97%) of FoxP2− neurons expressed tdTomato (teal). Only populations comprising ≥1% of GPe neurons are included.

Scale bar: 20 μm.

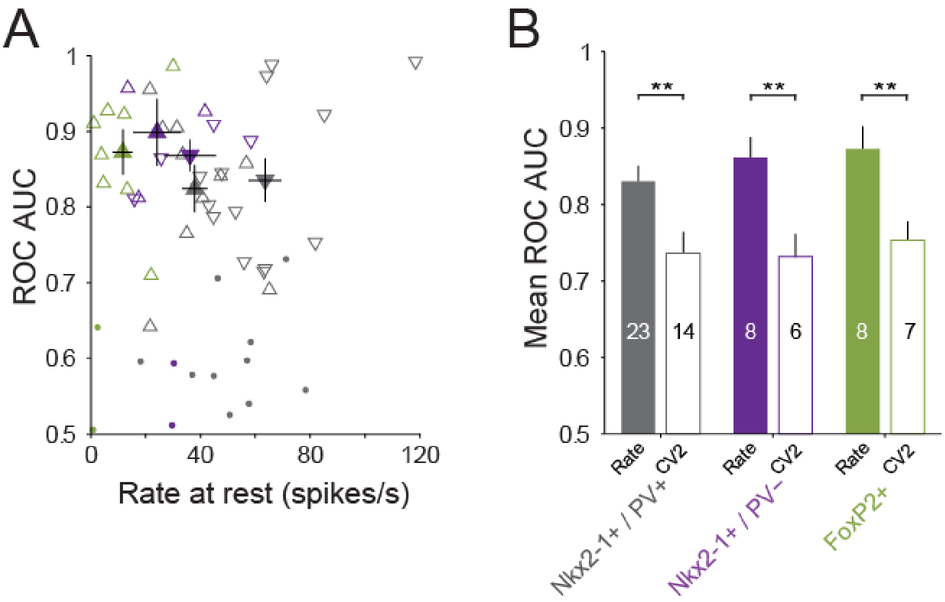

Figure S6 (related to Figure 7). Nkx2-1+/PV+ and Nkx2-1+/PV− prototypic neurons do not differ in their abilities to reliably encode movement.

The ability of Nkx2-1+/PV+ and Nkx2-1+/PV− prototypic neurons to encode movement was assessed using Receiver Operating Characteristic (ROC) analysis.

(A) Area under the ROC curve (AUC) plotted against firing rate during alert rest for all GPe neurons (Nkx2-1+/PV+ prototypic in grey, n = 33; Nkx2-1+/PV− prototypic in purple, n = 10; arkypallidal in green, n = 10). Filled circles represent individual neurons for which the AUC was not significantly different from shuffled data. Open triangles represent individual neurons that significantly encoded movement (Δ = increased rate; ∇ = decreased rate). Mean values for significantly encoding neurons of each group are indicated by filled triangles.

(B) Mean AUCs for Nkx2-1+/PV+ prototypic neurons (0.83 ± 0.02 for rate, 0.74 ± 0.03 for CV2), Nkx2-1+/PV− prototypic neurons (0.86 ± 0.02 for rate, 0.74 ± 0.02 for CV2), and arkypallidal neurons (0.87 ± 0.03 for rate, 0.75 ± 0.03 for CV2) able to significantly discriminate movement using firing rate or CV2 as a classifier (numbers of discriminating neurons are indicated within bars; **$p < 0.01$). Note that, irrespective of whether rates or CV2s were tested, there were no significant differences between the mean AUCs of Nkx2-1+/PV+, Nkx2-1+/PV− and arkypallidal neurons ($p > 0.05$; Kruskal-Wallis One-Way Analyses of Variance on ranks).

Data are represented as mean ± SEM.

SUPPLEMENTAL EXPERIMENTAL PROCEDURES

All experimental procedures on animals were conducted in accordance with the Animals (Scientific Procedures) Act, 1986 (United Kingdom). Unless noted otherwise, 3–4 month-old male mice were used for all experiments.

Animals

Stereological cell counts (Figures 1, S3 and 2) were performed using wildtype C57Bl6/J mice (Charles River) and hemizygous *Lhx6-EGFP* mice (STOCKTg(Lhx6-EGFP)BP221Gsat/Mmmh; Mutant Mouse Regional Resource Centers, USA), respectively. For fate-mapping experiments (Figures 3, S4 and S5) *Nkx2-1iCre;Z/EG* mice (Anastasiades and Butt, 2011) were generated by breeding hemizygous *Nkx2-1iCre* driver mice (Kessaris et al., 2006) with homozygous *Z/EG* reporter mice (Novak et al., 2000), *Lhx6iCre;RCE* mice were generated by breeding hemizygous *Lhx6iCre* driver mice (Fogarty et al., 2007) with homozygous *RCE:LoxP* reporter mice (Miyoshi et al., 2010), *Lhx6iCre;Ai9;Dlx1-Venus^{fl}* mice were generated by breeding hemizygous *Lhx6iCre*;*Ai9* driver/reporter mice (Fogarty et al., 2007; Madisen et al., 2010) with homozygous *Dlx1-Venus^{fl}* reporter mice (Rubin et al., 2010), and *Mash1BAC-CreER;RCE* mice were generated by crossing hemizygous *Mash1BAC-CreER* driver mice (Battiste et al., 2007; Miyoshi et al., 2010) with homozygous *RCE:LoxP* mice. To induce Cre-mediated recombination events after mating with male reporter mice, pregnant *Mash1BAC-CreER* dams were given 4 mg tamoxifen (Sigma, T5648; 20 mg/ml stock solution in corn oil) by gavage at E10.5, E12.5 or E13.5; the latter two stages were chosen to take advantage of most GPe neurons being born at E11-E12 (Nóbrega-Pereira et al., 2010). For staging of embryos, E0.5 was defined as noon of the day that the vaginal plug was detected.

Tissue processing for light microscopy

Mice were deeply anesthetized with pentobarbitone and transcardially perfused with 20 ml of 0.05 M phosphate-buffered saline, pH 7.4 (PBS), followed by 20 ml of fixative (4% paraformaldehyde in 0.1 M PB). Brains were then stored in fixative at 4ºC for ~24 h, before being sectioned (50 µm) in the coronal plane (Leica VT1000S). For studies in adult mice, brain sections containing rostral (−0.2 mm from Bregma), central (−0.45 mm) and caudal (−0.7 mm) aspects of GPe were selected

(Figure S1). For the supplementary study in *Lhx6iCre;Ai9;Dlx1-Venus^{fl}* mice at postnatal day (P) 0.5, two sections containing GPe were selected. After washing in PBS, sections were blocked in 'PBS-AzT' (PBS containing 0.02% sodium azide and TritonX-100 [0.3% or 0.03% for tissue from adult or neonates, respectively]) and 10% normal donkey serum (NDS) before being incubated overnight at room temperature in PBS-AzT containing 1% NDS and one or more of the following primary antibodies: rabbit anti-Nkx2-1 (1:500 dilution; Santa Cruz, sc-13040), mouse anti-Nkx2-1 (1:100; Leica Biosystems, Novacastra NCL-TTF-1), goat anti-FoxP2 (1:500; Santa Cruz, sc-21069; Reimers-Kipping et al., 2011), rabbit anti-Npas1 (1:500; gift of S.L. McKnight; Erbel-Sieler et al., 2004), guinea pig anti-parvalbumin (1:1000; Synaptic Systems, 195004), rat anti-EGFP (1:500; Nacalai Tesque, 04404), rabbit anti-PPE (1:5000; LifeSpan Biosciences, LS-C23084), mouse anti-human neuronal protein HuC/HuD ('HuCD'; 1:200; Life Technologies, A-21271). To optimize immunolabeling with mouse anti-Nkx2-1, rabbit anti-Npas1 and rabbit anti-PPE antibodies, we used a heat pre-treatment (2 h, 2 h, and 6 h, respectively) as a means of antigen retrieval (Mallet et al., 2012). In this study, all EGFP and Venus signals were enhanced using the anti-EGFP antibody detailed above. Expression of tdTomato in mice carrying the *Ai9* reporter allele (after Cre-mediated recombination) was revealed using a rabbit antibody raised against red fluorescent protein (1:1000; gift of T. Kaneko, mRFP1; Hioki et al., 2010). After washing off any unbound primary antibodies in PBS, sections were incubated overnight in appropriate secondary antibodies (all raised in donkey) that that were conjugated to the following fluorophores: AlexaFluor488 (1:500; Life Technologies), AMCA, AlexaFluor649 and Cy3 (1:250, 1:500 and 1:1000 respectively; Jackson ImmunoResearch).

Stereological sampling

Sections were scanned on an epifluorescence microscope (Zeiss Imager-M2) running Stereo Investigator software (MBF biosciences). Appropriate sets of filter cubes were used to image the fluorescence channels: AMCA (excitation 299-392 nm, beamsplitter 395 nm, emission 420-470 nm); AlexaFluor488 (excitation 450-490 nm, beamsplitter 495 nm, emission 500-550 nm); Cy3 (excitation 532-558 nm, beamsplitter 570 nm, emission 570-640 nm); and AlexaFluor649 (excitation 625-655 nm, beamsplitter 660 nm, emission 665-715 nm). Images of each of the channels were taken sequentially and separately to negate possible crosstalk of signal across channels. The borders of rostral, central and caudal GPe in adult mice were defined using a 5× 0.16 NA objective; a ventral border was conservatively assigned to separate the GPe from the functionally-distinct ventral pallidum (red lines in Figure S1). For the purposes of this study, it was

not necessary to determine absolute numbers or densities of GPe neurons expressing the molecular markers that were tested. We thus used a version of design-based stereology, the 'modified optical fractionator' (West, 1999). Stereological sampling was carried out over the entire GPe in both hemispheres, as sectioned at the designated rostral, central and caudal levels in adults or at the two levels in neonates. For each immunofluorescence protocol, a series of completely tessellated, z-stacked images were acquired using a 40× 1.3 NA oil-immersion objective and 1.0 µm steps ('optical sections') at depths of 2 to 12 µm from the upper surface of each section at the level of GPe. To minimize confounds arising from surface irregularities, neuropil within a 2 µm 'guard zone' at the upper surface was not imaged. This sampling strategy thus defined a 10-µm thick 'optical disector' that was used with abutting, unbiased rectangular counting frames (200 × 150 µm; consisting of two exclusion lines and two inclusion lines) to generate all cell counts and marker expression profiles (West, 1999). A neuron was only counted once through the series of optical sections when its nucleus came into sharp focus within the disector; neurons with nuclei already in focus in the top optical section of the disector were ignored. A neuron was classified as not expressing the tested molecular marker only when positive immunoreactivity could be observed in other cells on the same optical section as the tested neuron.

Electrophysiological recording and juxtacellular labeling of GPe neurons in awake mice

Twenty three adult C57BL/6j mice (Charles River) were head-fixed using a custom made stainless-steel post, surgically implanted above the right pallidum. For implantation, mice were anesthetized using 1–2% v/v isoflurane (Isoflo; Schering-Plough), and placed in a stereotaxic frame (Kopf). The analgesic buprenorphine (Vetergesic; 0.03 mg/kg, s.c.) was administered peri-operatively. Two 0.8 mm diameter steel screws were implanted in the skull, juxtaposed to the dura mater; one above the left frontal cortex (AP: 2 mm, ML: −2 mm, in relation to Bregma) and a reference screw above the left cerebellum. A coiled stainless-steel wire (AM systems) was implanted between the layers of cervical muscle to record electromyogram activity (EMG; filtered at 0.3–0.5 kHz, sampled at 20 kHz). The 'L-shaped' head-fixation post was adhered to the skull with cyanoacrylate adhesive (Loctite; Henkel UK). The post was positioned so that the 3 mm diameter hole in its base was above the right GPe (centered on AP: −0.4 mm, ML: +2.2 mm) and a discrete craniotomy was made within. The craniotomy was sealed with 'Kwik-cast' silicone sealant (World Precision Instruments) and the exposed skull and screws were encased in dental

acrylic (Jet Denture Repair; Lang Dental). The mice were then allowed to recover fully from surgery.

For electrophysiological recording, mice were placed on top of a custom 22 cm diameter Ethafoam running wheel and the head-restraint post attached to a stereotaxic frame using a custom holder. Extracellular recordings of action potentials (filtered at 0.3–5 kHz, gain of 1000×; ELX-01MX and DPA-2FS amplifiers from NPI Electronic Instruments; sampled as 20 kHz) fired by individual GPe neurons were made using glass electrodes (tip diameter ~1.3 µm; 10–30 MΩ *in situ*) containing 1.5% w/v Neurobiotin (Vector Labs) in 0.5 M NaCl (Janezic et al., 2013). Electrodes were lowered into the brain with submicron precision using a micromanipulator (IVM-1000; Scientifica). After recording, each neuron included here was juxtacellularly labeled with Neurobiotin. After allowing time for the diffusion of Neurobiotin within each neuron, the mouse was deeply anesthetized with pentobarbitone and transcardially perfused with fixative (see above).

To test the molecular profile of Neurobiotin-labeled GPe neurons, 50 µm parasagittal sections were incubated for 4 hrs at room temperature in PBS-AzT containing Cy3-conjugated streptavidin (1:3000; Life Technologies). Sections containing Neurobiotin-labeled neuronal somata were tested for immunoreactivity to molecular markers (see above); a neuron was classified as not expressing the tested marker only when positive immunoreactivity could be observed in other cells on the same focal plane as the Neurobiotin-labeled neuron.

Electrophysiological recording and juxtacellular labeling of GPe neurons in anesthetized mice

Extracellular recordings of individual GPe neurons were made in 12 anesthetized adult C57BL/6j mice. Anesthesia was induced with 1–2% v/v isoflurane and then maintained with urethane (1.5 g/kg, i.p.; ethyl carbamate, Sigma); supplemental doses of urethane (0.15 g/kg; i.p.) were given as required. Anesthetized mice were head fixed (see above) and laid upon a homeothermic heating mat (Harvard Apparatus). We focused our analysis on single-unit activity recorded in the GPe during robust slow-wave activity (SWA), as verified by simultaneous electrocorticogram (ECoG) recordings from the left frontal cortex (AP: 2 mm, ML: −2 mm). To extract SWA periods in an objective way, ECoG data were Fourier transformed (frequency resolution of 0.2 Hz), and the ratio of power in the SWA band (0.5–2 Hz) to power in the gamma band (30–80 Hz) was calculated. Epochs of contiguous data (each of >10 s in duration) where each ECoG data point

had a power ratio of >13 were then concatenated and used for further analysis (Janezic et al., 2013). After recording, all GPe neurons included here were juxtacellularly labeled with Neurobiotin and then recovered for immunohistochemical testing of molecular marker expression (see above).

Electrophysiological data analysis

Data were acquired and initially analyzed using Spike2 software (Cambridge Electronic Design). Putative single-unit activity was isolated with standard 'spike sorting' procedures, including template matching, principal component analysis, and supervised clustering (Spike2). Isolation of single units was verified by the presence of a distinct refractory period in the interspike interval (ISI) histograms. For analysis of the firing properties of neurons when mice were at rest, periods of movement were removed and the data were then concatenated. Mean firing rate (spikes/s) and mean CV2 were calculated from the total number of spikes. Mean CV2 is a measure of regularity (the lower the CV2 value, the more regular the unit activity) that is related to the coefficient of variation of the ISI but is less sensitive to alterations in firing rate (Holt et al., 1996): $CV2 = \sqrt{2}|(ISI_n - ISI_{n-1})| / (ISI_n + ISI_{n-1})$. Bursts were determined using a custom MATLAB (MathWorks) routine based on the Poisson surprise method (Elias et al., 2008; Legéndy and Salcman, 1985). For bursts, pairs of ISIs that had a mean ISI of less than half the mean ISI of the spike train were initially identified. From these 'seeds', the least probable group of successive spikes (compared to a Poisson spike train with the same mean rate, *r*) was determined. The Poisson surprise value (*S*) is the probability of *n* spikes in an epoch of *T* milliseconds such that $S = -\text{Log}\, P(n)$ where $P(n)$ is Poisson cumulative distribution function defined as follows:

$$\mathrm{P}(n) = e^{-rT} \sum_{i=n}^{n-1} (rT)^i / i!$$

Spikes following the seed were added to the seed one by one to maximize *S*; if addition of a spike failed to increase *S*, nine further attempts of adding subsequent spikes were made. Maximization of *S* was then attempted by omitting a spike from the beginning of the seed, if successful the process was iterated. Finally, further maximization of *S* was attempted by adding spikes preceding the seed (in a similar manner, but in the opposite direction, to the original spike addition process). The threshold for inclusion of the final burst period was set at $P(n)<0.05$ and data were only analyzed for bursts if recording epochs contained more than 60 spikes.

Peri-event time histograms (40 ms bin width) were smoothed with a sliding five-point Hamming window; varying bin size or smoothing parameters had no systematic effect on the results. To quantify how well the spike trains of individual GPe neurons could predict movement we used Receiver Operating Characteristic (ROC) analysis. We considered test epochs starting every 10 ms (the value of this parameter had no systematic effect on the results) and excluded epochs straddling the start or end of a movement period. To ensure that the test epoch was shorter than most movements (to avoid excluding movements from analysis), yet as long as possible (to maximize the amount of information within each epoch), we set the epoch on a neuron-by-neuron basis to be of a duration equal to half of the mean movement duration for the neuron being analyzed. For each epoch, we evaluated the firing rate and the regularity of firing (CV2) to obtain their distributions for epochs with and without movement. ROC curves were then constructed by plotting (for a series of rate or CV2 classification thresholds) the proportion of epochs containing movement and correctly classified as movement versus the fraction of epochs without movement but were falsely classified as movement. An area under the ROC curve (AUC) of 0.5 corresponds to chance classification, while an AUC of 1 corresponds to perfect discrimination. To assess statistical significance of the obtained AUC for a given neuron, a shuffling procedure was used. In each of the 1000 iterations, the shuffled data were created by shifting movement periods to random positions within the recording, and computing AUC for the shuffled data. The AUC for a neuron was considered significant if it was higher than the 95th percentile of the distribution of AUCs computed from shuffled data.

SUPPLEMENTAL REFERENCES

Anastasiades, P.G., and Butt, S.J.B. (2011). Decoding the transcriptional basis for GABAergic interneuron diversity in the mouse neocortex. Eur. J. Neurosci. *34*, 1542–1552.

Battiste, J., Helms, A.W., Kim, E.J., Savage, T.K., Lagace, D.C., Mandyam, C.D., Eisch, A.J., Miyoshi, G., and Johnson, J.E. (2007). Ascl1 defines sequentially generated lineage-restricted neuronal and oligodendrocyte precursor cells in the spinal cord. Development *134*, 285–293.

Elias, S., Ritov, Y., and Bergman, H. (2008). Balance of increases and decreases in firing rate of the spontaneous activity of basal ganglia high-frequency discharge neurons. J. Neurophysiol. *100*, 3086–3104.

Erbel-Sieler, C., Dudley, C., Zhou, Y., Wu, X., Estill, S.J., Han, T., Diaz-Arrastia, R., Brunskill, E.W., Potter, S.S., and McKnight, S.L. (2004). Behavioral and regulatory abnormalities in mice deficient in the NPAS1 and NPAS3 transcription factors. Proc. Natl. Acad. Sci. U. S. A. *101*, 13648–13653.

Fogarty, M., Grist, M., Gelman, D., Marín, O., Pachnis, V., and Kessaris, N. (2007). Spatial genetic patterning of the embryonic neuroepithelium generates GABAergic interneuron diversity in the adult cortex. J. Neurosci. *27*, 10935–10946.

Hioki, H., Nakamura, H., Ma, Y.F., Konno, M., Hayakawa, T., Nakamura, K.C., Fujiyama, F., and Kaneko, T. (2010). Vesicular glutamate transporter 3-expressing nonserotonergic projection neurons constitute a subregion in the rat midbrain raphe nuclei. J. Comp. Neurol. *518*, 668–686.

Janezic, S., Threlfell, S., Dodson, P.D., Dowie, M.J., Taylor, T.N., Potgieter, D., Parkkinen, L., Senior, S.L., Anwar, S., Ryan, B., et al. (2013). Deficits in dopaminergic transmission precede neuron loss and dysfunction in a new Parkinson model. Proc. Natl. Acad. Sci. U. S. A. *110*, E4016–E4025.

Kessaris, N., Fogarty, M., Iannarelli, P., Grist, M., Wegner, M., and Richardson, W.D. (2006). Competing waves of oligodendrocytes in the forebrain and postnatal elimination of an embryonic lineage. Nat. Neurosci. *9*, 173–179.

Legéndy, C.R., and Salcman, M. (1985). Bursts and recurrences of bursts in the spike trains of spontaneously active striate cortex neurons. J. Neurophysiol. *53*, 926–939.

Madisen, L., Zwingman, T.A., Sunkin, S.M., Oh, S.W., Zariwala, H.A., Gu, H., Ng, L.L., Palmiter, R.D., Hawrylycz, M.J., Jones, A.R., et al. (2010). A robust and high-throughput Cre reporting and characterization system for the whole mouse brain. Nat. Neurosci. *13*, 133–140.

Mallet, N., Micklem, B.R., Henny, P., Brown, M.T., Williams, C., Bolam, J.P., Nakamura, K.C., and Magill, P.J. (2012). Dichotomous organization of the external globus pallidus. Neuron *74*, 1075–1086.

Miyoshi, G., Hjerling-Leffler, J., Karayannis, T., Sousa, V.H., Butt, S.J.B., Battiste, J., Johnson, J.E., Machold, R.P., and Fishell, G. (2010). Genetic fate mapping reveals that the caudal ganglionic eminence produces a large and diverse population of superficial cortical interneurons. J. Neurosci. *30*, 1582–1594.

Nóbrega-Pereira, S., Gelman, D., Bartolini, G., Pla, R., Pierani, A., and Marín, O. (2010). Origin and molecular specification of globus pallidus neurons. J. Neurosci. *30*, 2824–2834.

Novak, A., Guo, C., Yang, W., Nagy, A., and Lobe, C. (2000). Z/EG, a double reporter mouse line that expresses enhanced green fluorescent protein upon Cre-mediated excision. Genesis *155*, 147–155.

Paxinos, G., and Franklin, K.B.J. (2007). The mouse brain in stereotaxic coordinates (Academic Press).

Reimers-Kipping, S., Hevers, W., Pääbo, S., and Enard, W. (2011). Humanized Foxp2 specifically affects cortico-basal ganglia circuits. Neuroscience *175*, 75–84.

Rubin, A.N., Alfonsi, F., Humphreys, M.P., Choi, C.K.P., Rocha, S.F., and Kessaris, N. (2010). The germinal zones of the basal ganglia but not the septum generate GABAergic interneurons for the cortex. J. Neurosci. *30*, 12050–12062.

West, M.J. (1999). Stereological methods for estimating the total number of neurons and synapses: issues of precision and bias. Trends Neurosci. *22*, 51–61.
