# Supplementary material for: Distinct Developmental Origins Manifest in the Specialized Encoding of Movement by Adult Neurons of the External Globus Pallidus
Source: Neuron. 2015 Apr 22;86(2):501–13. doi: 10.1016/j.neuron.2015.03.007 (PMC4416107; doi:10.1016/j.neuron.2015.03.007)
Supplement: Document S2. Article plus Supplemental Information [file mmc2.pdf]

Article

Neuron

Distinct Developmental Origins Manifest in the Specialized Encoding of Movement by Adult Neurons of the External Globus Pallidus

Highlights

- Arkypallidal and prototypic GPe cells have distinct origins and transcriptional codes
- Arkypallidal neurons rapidly and robustly increase firing rate at movement onset
- Movement is accurately encoded by single arkypallidal or prototypic neurons
- Two GPe cell types are fated to affect different targets in distinct ways in behavior

Authors

Paul D. Dodson, Joseph T. Larvin, ..., Simon J.B. Butt, Peter J. Magill

Correspondence

paul.dodson@pharm.ox.ac.uk (P.D.D.), peter.magill@pharm.ox.ac.uk (P.J.M.)

In Brief

Cell type specification in the developing brain informs on neuronal function in adult behavior. Dodson et al. demonstrate that distinct embryonic origins and transcriptional codes of two types of GABAergic pallidal projection neuron are reflected in their divergent encoding of movement.

Dodson et al., 2015, Neuron *86*, 501–513
April 22, 2015 ©2015 The Authors
http://dx.doi.org/10.1016/j.neuron.2015.03.007

CellPress

Distinct Developmental Origins Manifest in the Specialized Encoding of Movement by Adult Neurons of the External Globus Pallidus

Paul D. Dodson,[1,2,*] Joseph T. Larvin,[1] James M. Duffell,[1] Farid N. Garas,[1] Natalie M. Doig,[1] Nicoletta Kessaris,[3] Ian C. Duguid,[4] Rafal Bogacz,[1,5] Simon J.B. Butt,[6] and Peter J. Magill[1,2,*]
[1]Medical Research Council Brain Network Dynamics Unit, Department of Pharmacology, University of Oxford, Oxford OX1 3QT, UK
[2]Oxford Parkinson's Disease Centre, University of Oxford, Oxford OX1 3QX, UK
[3]Wolfson Institute for Biomedical Research and Department of Cell and Developmental Biology, University College London, London WC1E 6BT, UK
[4]Centre for Integrative Physiology, School of Biomedical Sciences, University of Edinburgh, Edinburgh EH8 9XD, UK
[5]Nuffield Department of Clinical Neurosciences, University of Oxford, Oxford OX3 9DU, UK
[6]Department of Physiology, Anatomy and Genetics, University of Oxford, Oxford OX1 3QX, UK
*Correspondence: paul.dodson@pharm.ox.ac.uk (P.D.D.), peter.magill@pharm.ox.ac.uk (P.J.M.)
http://dx.doi.org/10.1016/j.neuron.2015.03.007
This is an open access article under the CC BY license (http://creativecommons.org/licenses/by/4.0/).

SUMMARY

Transcriptional codes initiated during brain development are ultimately realized in adulthood as distinct cell types performing specialized roles in behavior. Focusing on the mouse external globus pallidus (GPe), we demonstrate that the potential contributions of two GABAergic GPe cell types to voluntary action are fated from early life to be distinct. Prototypic GPe neurons derive from the medial ganglionic eminence of the embryonic subpallium and express the transcription factor Nkx2-1. These neurons fire at high rates during alert rest, and encode movements through heterogeneous firing rate changes, with many neurons decreasing their activity. In contrast, arkypallidal GPe neurons originate from lateral/caudal ganglionic eminences, express the transcription factor FoxP2, fire at low rates during rest, and encode movements with robust increases in firing. We conclude that developmental diversity positions prototypic and arkypallidal neurons to fulfil distinct roles in behavior via their disparate regulation of GABA release onto different basal ganglia targets.

INTRODUCTION

Neuronal cell type is programmed during brain development according to a tailored, combinatorial transcription factor code, with some code elements also being required to maintain the identities of postmitotic neurons throughout life (Deneris and Hobert, 2014). Past work, and particularly that on laminated structures like the spinal cord, retina, and cortex, has begun to unravel how transcriptional codes dictate the generation, specification, migration, and synaptic maturation of distinct neuronal populations (Arber, 2012; Jessell, 2000; Kepecs and Fishell, 2014; Molyneaux et al., 2007; Siegert et al., 2012). The extent to which these developmental processes map onto the neural coding of adult behavior in "real time" is unclear, but will likely vary in a cell-type-specific manner. The exploitation of transcriptional codes to resolve the contributions of different cell types to behavior holds particular promise in non-laminated brain structures comprising different projection cell types that intermingle and share the same neurotransmitter phenotype, as is the case with the external globus pallidus (GPe).

The GPe is a key component of basal ganglia circuits that govern movement and cognition, and is considered to have uniform cellular constituents in classical schemes of basal ganglia organization (DeLong, 1990; Mink, 1996; Smith et al., 1998; Wichmann and DeLong, 1996). Despite the great utility of such conceptual schemes, the GPe is more realistically viewed as being comprised of different populations of GABAergic neuron (Kita, 2007). This is especially evident in the Parkinsonian GPe wherein two major GABAergic cell types, "prototypic" and "arkypallidal" neurons, exhibit distinct firing under anesthesia (Mallet et al., 2012). Importantly, prototypic and arkypallidal neurons also project to distinct sets of basal ganglia targets; the former cell type innervates the subthalamic nucleus (STN) and basal ganglia output nuclei, whereas the latter only innervates the striatum (Mallet et al., 2012). Cellular heterogeneity in the adult GPe might well stem from early stages of brain development. For example, most (but not all) GPe neurons derive from the medial ganglionic eminence (MGE) of the embryonic subpallium (Flandin et al., 2010; Nóbrega-Pereira et al., 2010). However, diverse ontogeny has yet to be framed in the context of functionally defined cell types in the adult, dopamine-intact GPe.

To link the early-life development of GPe neurons to their dynamic encoding of behavior in adults, we traced the embryonic origins of prototypic and arkypallidal neurons and also recorded the firing of individual, molecularly identified neurons in awake mice. We show that prototypic and arkypallidal neurons are derived from distinct pools of subpallial progenitors and are endowed with cell-type-specific transcriptional codes. This diversity translates to the adult GPe wherein the two cell types exhibit

CrossMark

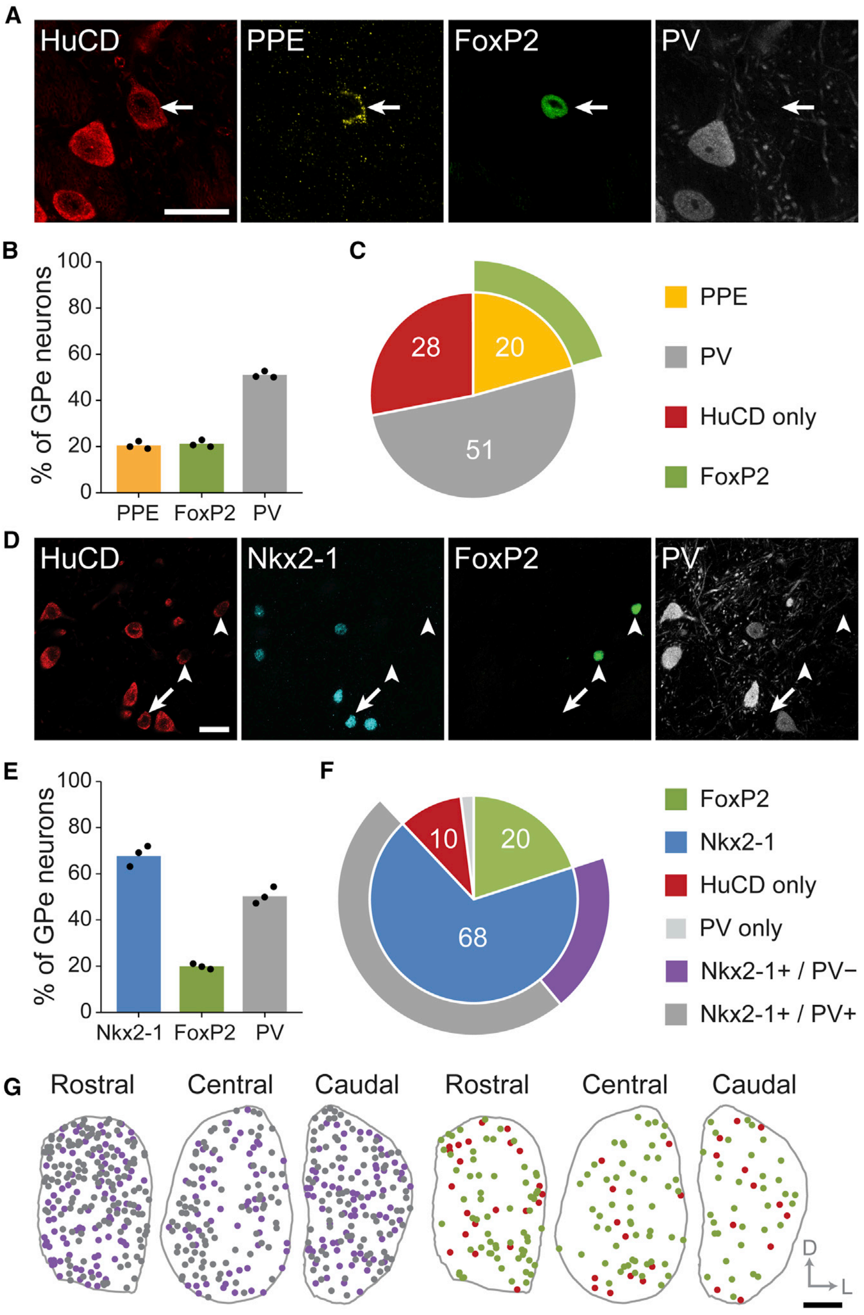

Figure 1. Molecular Heterogeneity of Neurons in the Adult GPe

Immunofluorescence identification of arkypallidal neurons (A)–(C) and prototypic neurons (D)–(F).

(A) The pan-neuronal marker HuCD was used to label all GPe neurons. Preproenkephalin (PPE)-expressing GPe neurons (arrow) also express FoxP2, but not parvalbumin (PV).

(B) Mean expression profiles for PPE, FoxP2, and PV. Filled circles in this and subsequent profiles represent counts from individual animals.

(C) Proportions of GPe neurons (i.e., all HuCD+ neurons) expressing different molecular markers. In this and subsequent pie charts, outer segments represent expression overlap of inner populations with another marker (e.g., all PPE+ neurons also expressed FoxP2, and vice versa), and only populations comprising ≥ 1% of GPe neurons are included. One-fifth of GPe neurons co-express PPE and FoxP2, while another quarter of GPe neurons (“HuCD only”) do not express PPE, FoxP2, or PV.

(D) Nkx2-1+ GPe neurons are numerous; some lack PV (arrows). FoxP2+ neurons (arrowheads) do not express Nkx2-1.

(E) Expression profiles for Nkx2-1, FoxP2, and PV.

(F) Most Nkx2-1+ neurons co-express PV. A minority of HuCD+ neurons express PV, but not any other tested marker (“PV only”).

(G) Representative example (single animal) of locations of neurons expressing different molecular markers at three rostro-caudal levels of GPe. Color code as in (F). For clarity, prototypic neurons (Nkx2-1+/PV+ and Nkx2-1+/PV−, left) are separated from arkypallidal (FoxP2+) and other GPe neurons (right). D, dorsal; L, lateral.

Scale bars in (A) and (D), 20 μm; (G), 200 μm.

distinct firing rates/patterns at rest and during the execution of voluntary movements, suggesting they perform dedicated, complementary roles according to their lineages.

RESULTS

Two Major Types of GPe Neuron Are Delineated by Transcription Factor Expression

Arkypallidal neurons express the neuropeptide precursor preproenkephalin (PPE), whereas prototypic neurons do not; however, many prototypic neurons express the calcium-binding protein parvalbumin (PV) (Mallet et al., 2012). As a first step toward characterizing the development and functions of arkypallidal and prototypic neurons in the dopamine-intact brain, we sought to define more comprehensive sets of molecular markers with which to discriminate the two cell types unambiguously. Using unbiased, stereological cell counting in adult wild-type mice (see Figure S1), we determined that PPE-expressing (PPE+) GPe neurons also expressed the transcription factor forkhead box protein P2 (FoxP2), and vice versa, and that PPE+/FoxP2+ arkypallidal neurons constitute 20% of all GPe neurons (Figures 1A–1C). The majority of GPe neurons in the rodent and human brain express a different transcription factor, NK2 homeobox 1 (Nkx2-1), and most Nkx2-1+ GPe neurons also express PV (Flandin et al., 2010; Magno et al., 2009, 2011; Nóbrega-Pereira et al., 2010). As such, we reasoned that Nkx2-1 expression in the GPe might be selective for prototypic neurons, irrespective of PV expression. We determined that Nkx2-1+ neurons constitute 68% of all GPe neurons, and that two-thirds of Nkx2-1+ neurons co-expressed PV (Figures

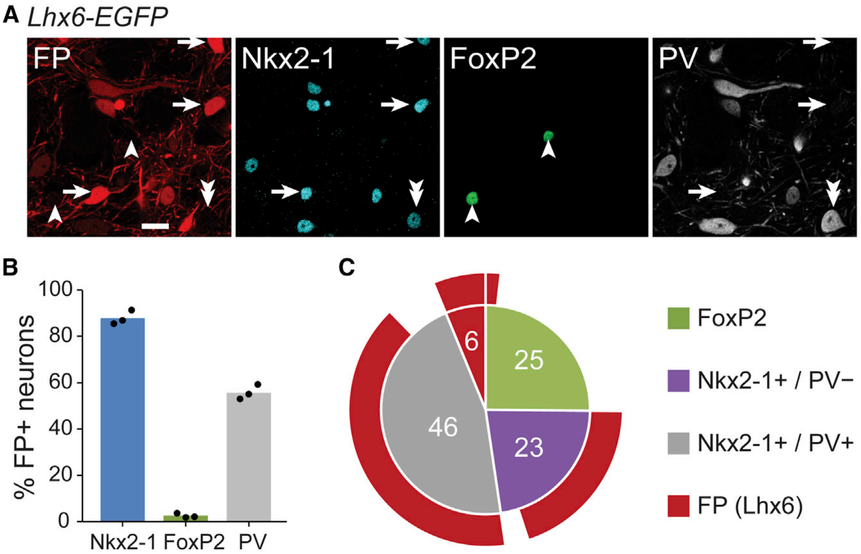

Figure 2. The Majority of Prototypic GPe Neurons Co-Express Lhx6

(A) Immunofluorescence labeling of GPe neurons in an *Lhx6-EGFP* mouse (FP, fluorescent protein; pseudocolored red). Most FP+ neurons co-expressed Nkx2-1 (arrows), but not FoxP2 (arrowheads). Some Nkx2-1+ neurons did not express FP (double arrowhead).

(B) Expression profiles of FP+ neurons.

(C) Proportions of GPe neurons expressing FP alone, Nkx2-1 (with or without PV), or FoxP2. Note that many PV+ and PV− prototypic neurons were also FP+.

Scale bar, 20 μm.

1D–1F). Importantly, however, co-expression of Nkx2-1 and FoxP2 was negligible (Figures 1D–1F). These data suggest that Nkx2-1 is a highly selective marker of prototypic neurons, the most numerous GABAergic cell type in the mouse GPe. Consistent with the mapping of a small number of identified arkypallidal and prototypic neurons in the rat (Mallet et al., 2012), FoxP2+ neurons and Nkx2-1+ neurons were not confined to a particular region of GPe, but were instead intermingled throughout this nucleus (Figure 1G). Previous work in anesthetized, Parkinsonian rats has shown that the spontaneous firing rates of arkypallidal neurons tend to be much lower than those of prototypic GPe neurons during cortical slow-wave activity (Mallet et al., 2012). To test whether the same physiological distinction applies in the dopamine-intact mouse GPe, we extracellularly recorded and juxtacellularly labeled single GPe neurons in anesthetized adult mice (see Figure S2). We determined that, during cortical slow-wave activity, Nkx2-1+ prototypic GPe neurons tended to fire at high rates (~22 spikes/s; Figure S2A). In contrast, FoxP2+ arkypallidal neurons fired at significantly lower rates (~4 spikes/s; $p < 0.01$, t test; Figures S2B and S2C). On the basis of these molecular profiling experiments and electrophysiological recordings, we conclude that arkypallidal and prototypic neurons can be defined by their mutually exclusive expression of FoxP2 and Nkx2-1, respectively.

To further explore the molecular diversity of GPe neurons, we extended our analyses to account for LIM homeobox 6 (Lhx6), which acts downstream of *Nkx2-1* to direct the migration and specification of several telencephalic cell types, including some GPe neurons (Du et al., 2008; Flandin et al., 2010; Liodis et al., 2007; Sussel et al., 1999; Zhao et al., 2008), although its roles in GPe are relatively subtle and can be partly fulfilled by other transcription factors (Flandin et al., 2011; Zhao et al., 2008). We examined EGFP immunoreactivity in *Lhx6-EGFP* mice as a proxy for expression of this transcription factor (Flandin et al., 2010). We determined that the vast majority (88%) of EGFP+ GPe neurons were also Nkx2-1+, and vice versa (Figure 2). The proportions of PV+ or PV− prototypic (Nkx2-1+) neurons that co-expressed EGFP were similar (87% and 88%, respectively). However, immunofluorescence signals for EGFP tended to be highest in PV− neurons (Figure 2A, white arrows), suggesting that Lhx6 expression might be lower in PV+ neurons. These data imply that Lhx6 is an additional marker of prototypic neurons in the adult GPe. In agreement with this, very few (< 3%) EGFP+ neurons also expressed FoxP2 (Figures 2A–2C).

We also analyzed the expression of neuronal PAS domain protein 1 (Npas1), a transcription factor which marks some Nkx2-1+ neurons and a distinct set of Nkx2-1− neurons in GPe (Flandin et al., 2010; Nóbrega-Pereira et al., 2010). We observed that one-third of adult GPe neurons express Npas1 (Figure S3). Co-expression of FoxP2 and Npas1 was common; most (87%) FoxP2+ arkypallidal neurons co-expressed Npas1, and many (56%) Npas1+ neurons co-expressed FoxP2 (Figure S3). A minority (12%) of Nkx2-1+ neurons co-expressed Npas1 (most of these prototypic neurons were PV−; Figure S3). These data show that, although the majority (85%) of Nkx2-1−/Npas1+ GPe cells are arkypallidal neurons, Npas1 expression alone does not neatly discriminate arkypallidal from prototypic neurons.

Arkypallidal and Prototypic GPe Neurons Arise from Distinct Developmental Lineages

The use of transcription factor expression to classify arkypallidal and prototypic neurons has relevance for understanding the developmental origins of these GPe cell types. Indeed, *Nkx2-1* is expressed throughout the proliferative ventricular zone of the MGE in the embryonic subpallium/ventral telencephalon (Butt et al., 2005; Flames et al., 2007; Long et al., 2009; Nóbrega-Pereira et al., 2010; Sussel et al., 1999), suggesting that the MGE is the progenitor domain of origin of most GPe neurons (Flandin et al., 2010; Nóbrega-Pereira et al., 2010; Sussel et al., 1999). However, as much as one quarter of all GPe cells are generated outside the Nkx2-1+ MGE (Nóbrega-Pereira et al., 2010). The origin(s) of this second population of GPe neurons is not well defined, but the lateral ganglionic eminence (LGE) is a reasonable candidate (Nóbrega-Pereira et al., 2010). Given that mature arkypallidal neurons make up almost one quarter of all GPe cells and do not express Nkx2-1, we hypothesized that their developmental lineage is distinct from that of prototypic GPe neurons. To

test this, while also accounting for the possibility that arkypallidal neurons might originate from the MGE, but then downregulate Nkx2-1 expression during their development, we used a recombinase-based genetic fate-mapping approach (Jensen and Dymecki, 2014). We first examined the GPe of adult *Nkx2-1iCre;Z/EG* mice (Anastasiades and Butt, 2011; Kessaris et al., 2006) that are designed to indelibly label (with fluorescent protein) all neurons that expressed *Nkx2-1* at any time during their development. We determined that only a tiny fraction (1.5%) of EGFP+ neurons also expressed FoxP2 (Figures 3A and 3B), consistent with our finding in wild-type mice that adult arkypallidal neurons do not express Nkx2-1. Almost all EGFP+ neurons co-expressed Nkx2-1 (Figure 3A), suggesting that few GPe neurons downregulate Nkx2-1 expression during development. However, about one-third of Nkx2-1+ GPe neurons did not express EGFP (Figures 3A and 3B). Although we cannot rule out the possibility that the *Z/EG* reporter gene was not active in this subset of prototypic neurons, this discrepancy likely arose because these *Nkx2-1iCre;Z/EG* mice do not capture Nkx2-1+ progenitors in the most dorsal, sulcal region of MGE (Fogarty et al., 2007), leaving open the possibility that arkypallidal neurons might still be generated from progenitors in this restricted region. To test whether this was the case, we carried out a second fate-mapping study using *Lhx6iCre;RCE* mice that capture and label with fluorescent protein virtually all MGE-derived neurons (Fogarty et al., 2007; Miyoshi et al., 2010). Consistent with our observations in *Lhx6-EGFP* mice (Figure 2), there was a high overlap of EGFP and Nkx2-1 expression in GPe neurons in the *Lhx6iCre;RCE* mice (91% of EGFP+ neurons were Nkx2-1+, and 96% of Nkx2-1+ neurons were EGFP+; Figures 3C and 3D). Thus, prototypic GPe neurons likely derive from the MGE. In agreement with this, and as could be expected (Figure 2), two-thirds of the EGFP+ GPe neurons in *Lhx6iCre;RCE* mice also expressed PV. Importantly though, the proportion of EGFP+ neurons co-expressing the arkypallidal neuron marker FoxP2 was negligible (< 1%) (Figures 3C and 3D). In summary, these two fate-mapping experiments concurrently indicate that arkypallidal neurons are not derived from the MGE (nor from any other cells expressing *Nkx2-1* or *Lhx6*), and thus, that their developmental origin is distinct from that of prototypic neurons.

Within the embryonic subpallium, FoxP2 expression is prominent in the mantle and subventricular zones of the LGE, less abundant in the caudal ganglionic eminence (CGE), and relatively scant in MGE (Ferland et al., 2003; Long et al.,

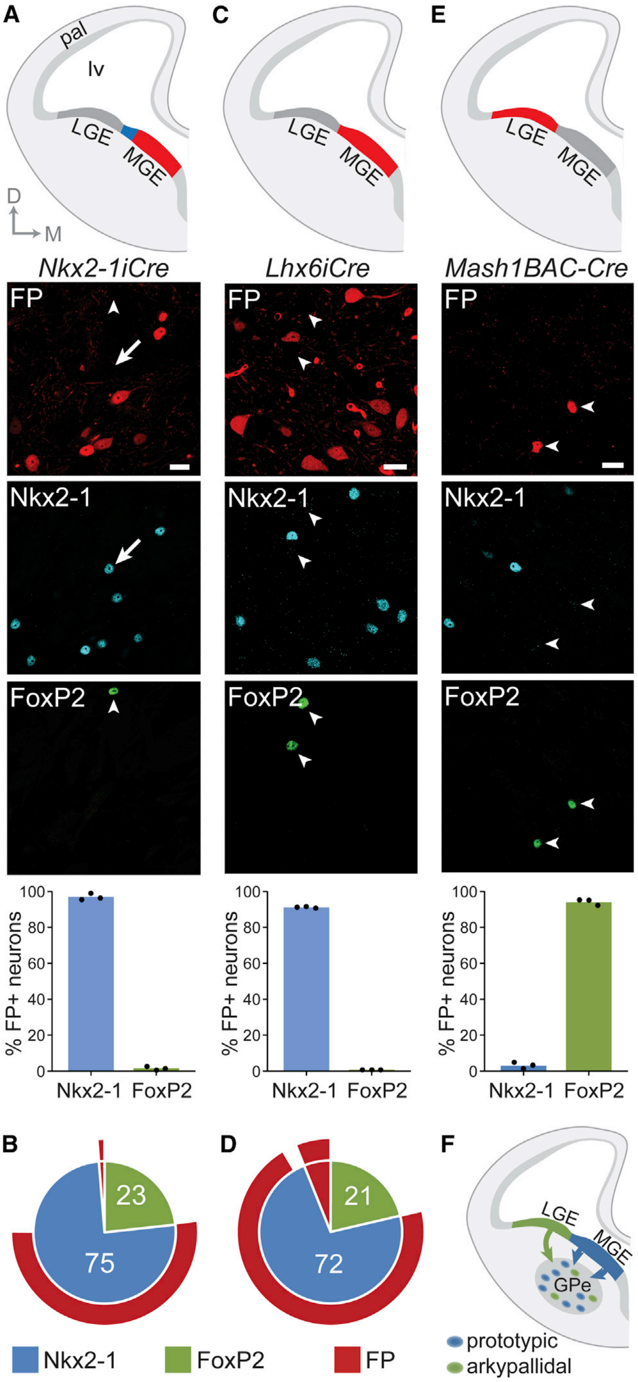

Figure 3. Arkypallidal and Prototypic GPe Neurons Have Distinct Embryonic Lineages

(A, C, and E) Immunofluorescence labeling and marker expression profiles in three lines of mice used for fate mapping of GPe neurons (FP, fluorescent protein; pseudocolored red). These mice report neurons derived from different embryonic progenitor domains; the regions of the medial ganglionic eminence (MGE) or lateral/caudal ganglionic eminences (LGE/CGE) reported by each mouse line are highlighted in red in the top schematics of embryonic brain. D, dorsal; M, medial; pal, pallium; lv, lateral ventricle.

(A and B) In *Nkx2-1iCre;Z/EG* mice, which report most MGE-derived neurons, 97% FP+ neurons co-express Nkx2-1. However, only 68% of Nkx2-1+ neurons were also FP+ (B). Thus, a substantial proportion of Nkx2-1+ neurons are not captured by this mouse line (also see arrow in A), likely those emanating from the dorsal-most region of MGE (blue region in top schematic). FP+ neurons in these mice did not co-express FoxP2 (arrowhead).

(C and D) In *Lhx6iCre;RCE* mice, which report nearly all MGE-derived neurons, the vast majority of FP+ neurons expressed Nkx2-1, and vice versa. FP+ neurons in these mice did not co-express FoxP2 (arrowheads).

(E) In *Mash1BAC-CreER;RCE* mice, which report neurons derived from LGE/CGE, the vast majority of FP+ neurons co-expressed FoxP2 (arrowheads), but not Nkx2-1.

(F) Schematic summary of fate-mapping experiments. Arkypallidal (FoxP2+) neurons derive from the LGE/CGE of the embryonic subpallium, whereas prototypic (Nkx2-1+) neurons derive from the MGE.

Scale bars, 20 μm.

2009; Takahashi et al., 2003). It is thus plausible that arkypallidal neurons are derived from the LGE and/or CGE. To test this hypothesis, we performed a third fate-mapping experiment using *Mash1BAC-CreER;RCE* mice (Miyoshi et al., 2010). In this particular *Mash1BAC-CreER* driver mouse line, a tamoxifen-inducible form of Cre recombinase is expressed under the control of the *cis*-regulatory elements of the transcription factor *Mash1* (*Ascl1*), but, by chance, is almost exclusively restricted to Mash1-expressing cells in the LGE and CGE (Miyoshi et al., 2010). Following embryonic day (E)12.5 tamoxifen administration, we observed that only a small proportion (3%) of EGFP+ neurons in the adult GPe expressed Nkx2-1 (Figure 3E), thus confirming that this genetics-based strategy does not label neurons derived from the MGE at the peak point of neurogenesis from this eminence (Miyoshi et al., 2010). In contrast, the vast majority (94%) of EGFP+ neurons co-expressed FoxP2 (Figure 3E). This key observation also held true following tamoxifen administration at E10.5 or E13.5 (Figure S4A). The proportion of FoxP2+ neurons co-expressing EGFP after tamoxifen administration at E12.5 or E13.5 was greater than that after administration at E10.5 (Figure S4B), consistent with most GPe neurons being generated after E10.5 (Nóbrega-Pereira et al., 2010). To further test the hypothesis that arkypallidal neurons are derived from LGE/CGE, we performed a fourth fate-mapping experiment using neonatal *Lhx6iCre;Ai9;Dlx1-Venus*^{fl} mice (Rubin et al., 2010) that can simultaneously report on MGE-derived neurons (through Cre-mediated activation of tdTomato fluorescent protein) and LGE/CGE-derived neurons (by continued expression of a "floxed" Venus fluorescent protein). We observed that < 1% of FoxP2+ GPe neurons co-expressed tdTomato, and vice versa (Figure S5), which supports our analyses in other mouse lines showing that arkypallidal neurons are not derived from the MGE (Figure 3). We also observed that almost all (96%) Venus+ GPe neurons co-expressed FoxP2, and that most (89%) FoxP2+ GPe neurons co-expressed Venus (Figure S5). Taking the results of our four complementary fate-mapping experiments together, we conclude that prototypic GPe neurons are derived from the MGE, whereas arkypallidal neurons are derived from the LGE/CGE (Figure 3F).

Arkypallidal and Prototypic GPe Neurons Have Distinct Firing Properties in Awake Mice at Rest

Having established that arkypallidal and prototypic GPe neurons have distinct embryonic origins and express different sets of transcription factors, we sought to define whether and to what extent such diversity translates to the functional roles played by these two cell types in the adult brain. To achieve this, we extracellularly recorded the unperturbed spike firing of individual GPe neurons in awake, head-fixed wild-type mice. After recording, each GPe neuron included in this study was juxtacellularly labeled with Neurobiotin (Mallet et al., 2012), and then tested post hoc for FoxP2 and Nkx2-1 immunoreactivity; FoxP2+/Nkx2-1− GPe neurons were designated as arkypallidal neurons, whereas Nkx2-1+/FoxP2− neurons were identified as prototypic neurons. The mice were habituated to head fixation, but were not trained in any task. They were supported by a foam wheel during recordings; they typically rested on the wheel, but occasionally ran or made brief movements and postural adjustments (see below). In comparing the firing properties of identified arkypallidal and prototypic neurons, we first isolated electrophysiological data epochs recorded when the mice were at rest (Figure 4). During this behavioral state of alert immobility, identified prototypic neurons fired at high rates (~50 spikes/s), and in a tonic manner with occasional brief bursts and pauses (Figures 4A and 4C). This activity profile is similar to that of the majority of GPe neurons (of unknown cell type) recorded in dopamine-intact awake rats, cats, and primates (Anderson and Turner, 1991; Benhamou et al., 2012; Boraud et al., 1998; DeLong, 1971; Elias et al., 2007; Jaeger et al., 1995; Sachdev et al., 1989). In stark contrast, identified arkypallidal neurons fired sporadically and at relatively low rates (~10 spikes/s; Figures 4B and 4C). Compared to prototypic neurons, arkypallidal neurons had a significantly lower firing rate ($p < 0.001$, Mann-Whitney; Figure 4C), and fired less regularly (quantified with CV2; $p < 0.001$, Mann-Whitney; Figure 4D) with more bursts ($p < 0.001$, t test; Figure 4E). Because we recorded prototypic and arkypallidal neurons throughout the GPe (Figure 4F), these differences were not likely a consequence of biased sampling in a specialized neural subcircuit. These data suggest that, during alert rest, the collective activities (and thus, outputs) of arkypallidal and prototypic GPe neurons are profoundly different.

Firing of Arkypallidal and Prototypic GPe Neurons Is Different during Spontaneous Movement

We next tested whether the differences between arkypallidal and prototypic GPe neurons extended to their representation of spontaneous voluntary movements. The movement-related firing of GPe neurons has been most extensively studied in primates, and has been shown to be dependent upon several factors including the body region involved, the kinematic features of the movement, the potential outcome of moving (e.g., related to "reward prediction"), and whether a movement is passive, active, or externally cued (Anderson and Turner, 1991; Arkadir et al., 2004; DeLong et al., 1985; Georgopoulos et al., 1983; Hamada et al., 1990; Mitchell et al., 1987; Turner and Anderson, 2005). This complexity in neural dynamics, coupled with the challenge of post hoc identification of the recorded cell types, necessitated that we sample a small array of behaviors that were not biased to a particular body part, kinematic parameter, or experimenter-derived factor. We therefore focused our analyses on the GPe neuron activity recorded around brief (< 1 s) spontaneous movements, which typically involved the mouse altering whole-body posture to adjust its position on the wheel. We reasoned that, despite the heterogeneous kinematics of these self-paced voluntary movements, any consistent neuronal responses that emerged would reflect general organizational or coding principles of GPe cell types. In support of this, we observed that many GPe neurons robustly changed their firing during each defined movement period (Figure 5). To quantify and compare changes in firing across different sets of neurons, we first normalized firing rates as Z scores; the majority of prototypic neurons (40/43) exhibited significant firing rate changes during movement (with significance defined as more than two SDs from baseline, i.e., a Z score of ± 2). Just over half (22/40) of the "movement-responsive" prototypic neurons decreased their firing rate, while the remaining 18 prototypic neurons

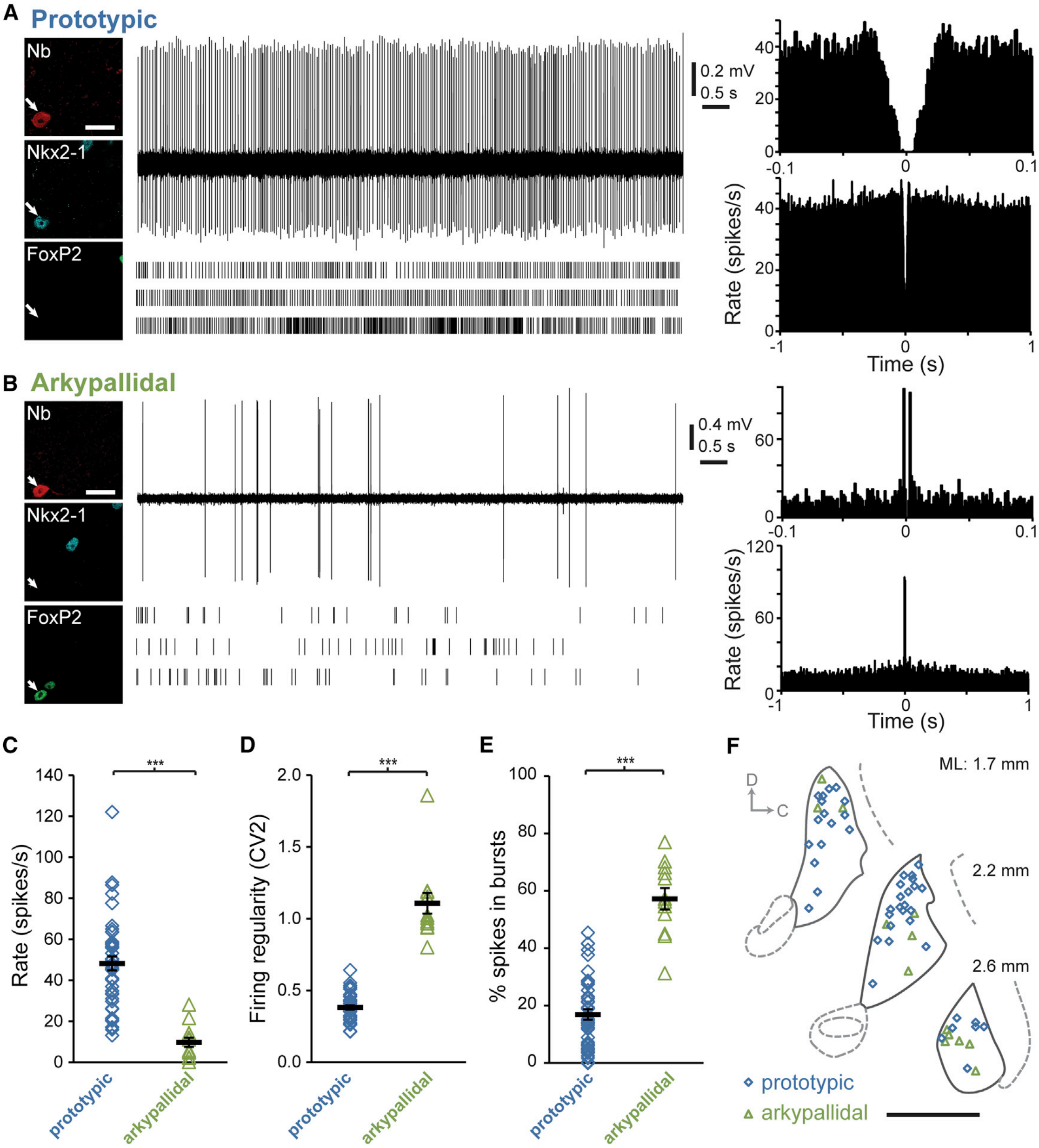

Figure 4. Firing Properties of Identified GPe Neurons in Awake Mice at Rest

(A and B) Typical single-unit activity (top), extended raster plots (bottom), autocorrelograms (right), and expression profiles (far left; scale bars, 20 μm) of a prototypic neuron (A) and an arkypallidal neuron (B). Individual neurons were juxtacellularly labeled with Neurobiotin (Nb) after recording; prototypic neurons expressed Nkx2-1 (but not FoxP2), whereas arkypallidal neurons expressed FoxP2 (but not Nkx2-1).

(C) Mean firing rate of prototypic neurons was significantly higher than that of arkypallidal neurons (48.3 ± 3.4 versus 9.8 ± 2.3 spikes/s; n = 44 and 13 neurons, respectively).

(D) Firing of prototypic neurons was more regular than that of arkypallidal neurons (CV2 of 0.38 ± 0.02 versus 1.11 ± 0.07).

(E) Prototypic neurons fired fewer spikes within bursts as compared to arkypallidal neurons (16.9% ± 1.8% versus 57.3% ± 3.7% of spikes; n = 44 and 12 neurons, respectively).

(F) Schematic parasagittal sections (D, dorsal; C, caudal) denoting the locations within the GPe of all recorded and identified neurons. Mediolateral (ML) distance from Bregma is shown on right. Scale bar, 1 mm.

Data are represented as mean ± SEM; ***$p < 0.001$.

increased their rate. Despite these differences between prototypic neurons, the response "polarity" of a given neuron (i.e., a decrease or increase in rate) was consistent from movement to movement (Figures 5A–5C). In contrast to the heterogeneous responses of prototypic neurons, all responsive arkypallidal neurons exhibited robust increases in their firing rate during

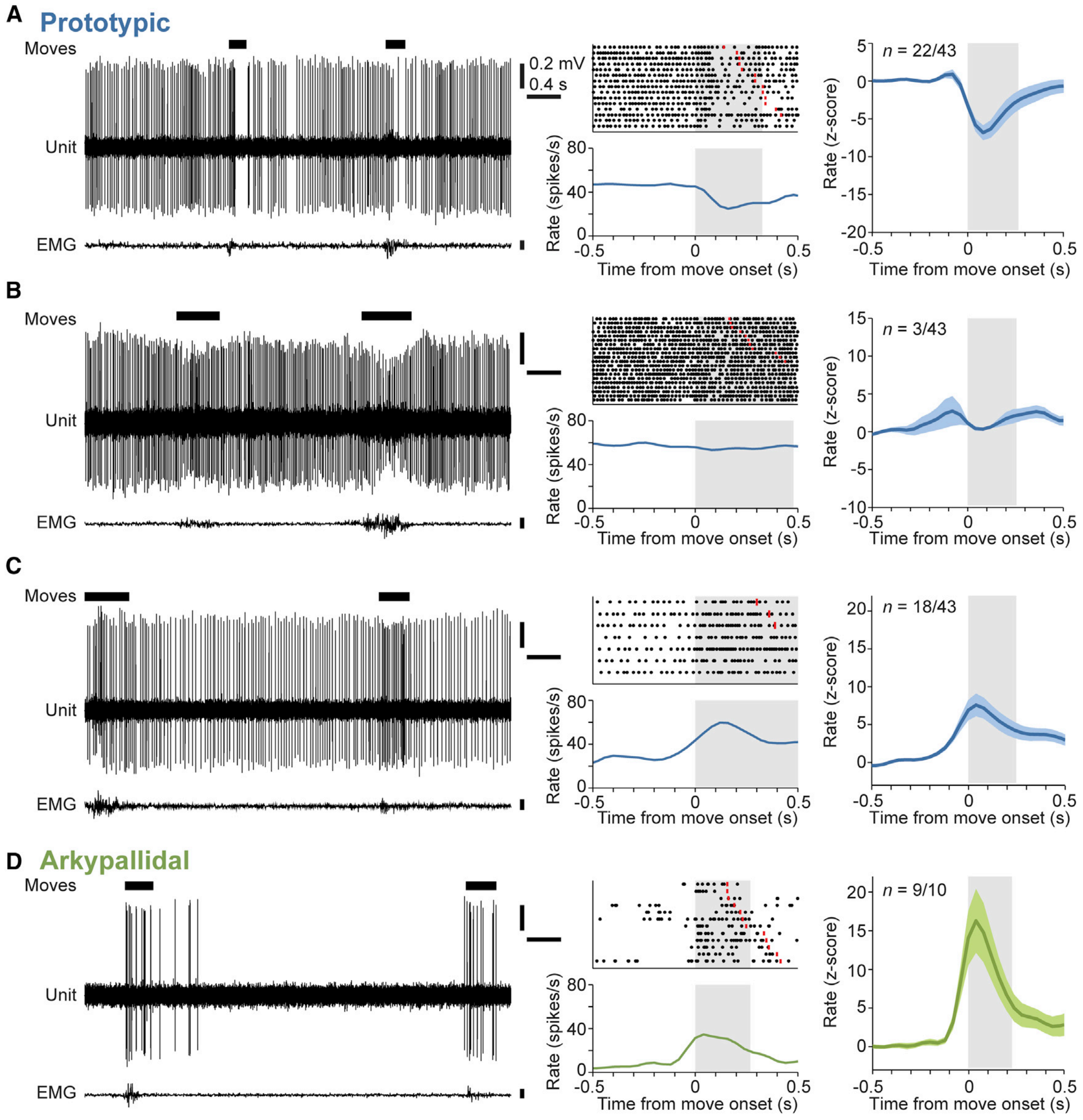

Figure 5. Activity Changes of Arkypallidal and Prototypic GPe Neurons Are Different during Spontaneous Movement

(A–D) Example single-unit activity (left), electromyograms (EMG, lower traces), and individual peri-event time histograms (PETH) and raster plots for three different prototypic (Nkx2-1+/PV+) GPe neurons (A)–(C) and an arkypallidal (FoxP2+) neuron (D) during brief spontaneous movements (denoted by black bars). Prototypic neurons (n = 43) could be subdivided into three groups based on the polarity of their firing rate changes during movement (mean, normalized PETH ± SEM shown for each group, right).

(A) During movement, 22 of 43 prototypic neurons significantly decreased their firing rate.

(B) During movement, three prototypic neurons showed no significant change.

(C) During movement, 18 prototypic neurons significantly increased their rate.

(D) In contrast, arkypallidal neurons (n = 10) uniformly increased their firing rates during movement (nine showed significant increases).

Mean movement duration is denoted by gray shading. In raster plots, the end of individual movement epochs is indicated by red lines.

movement (Figure 5D). Irrespective of response polarity, changes in the firing rates of both cell types evolved rapidly (with peak responses occurring within 100–150 ms of movement onset) (Figure 5). The peak decrease in firing rate of prototypic neurons occurred at a significantly longer latency than that of the peak increase in arkypallidal neuron firing ($p < 0.01$, Mann-Whitney). These differences in the polarities, timing, and relative heterogeneity of responses of prototypic and arkypallidal neurons did not arise from any systematic differences in the movements recorded with each cell type. Indeed, the average durations of movement periods recorded with prototypic or arkypallidal neurons were similar ($p > 0.05$, t test). However, because almost one-third of prototypic neurons do not express PV (Figure 1F), we considered whether the heterogeneity of responses

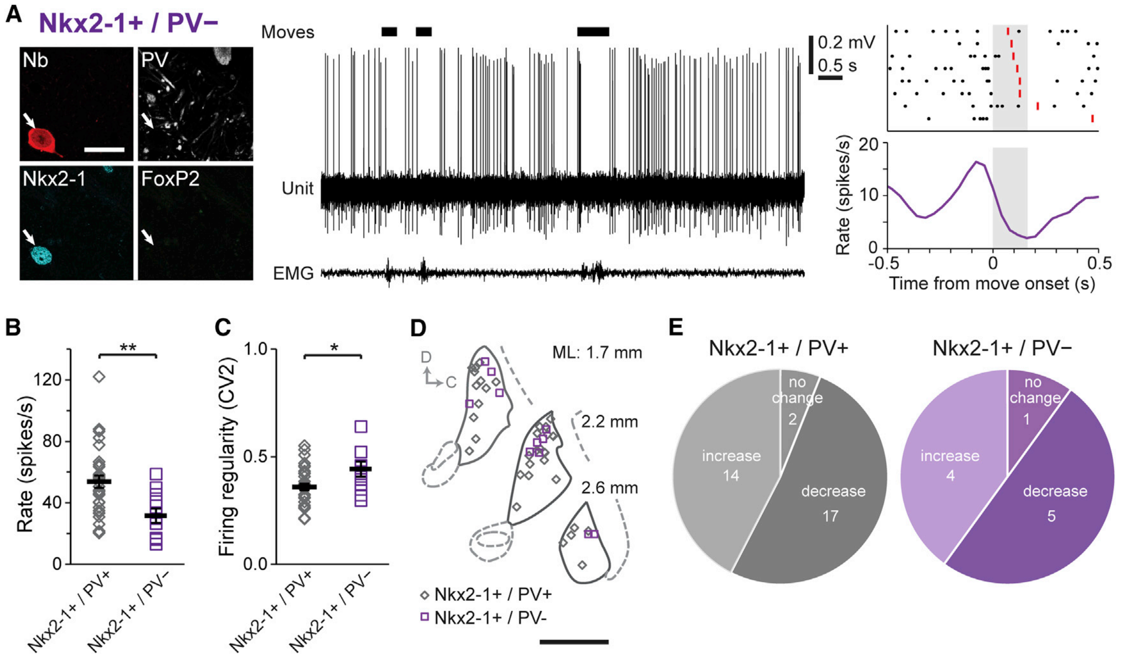

Figure 6. Differential Expression of Parvalbumin by Prototypic Neurons Does Not Account for Their Heterogeneous Firing during Movement

(A) Single-unit activity (with corresponding EMG and movement epochs) and PETH (with corresponding raster plot) of a prototypic (Nkx2-1+) neuron that did not express parvalbumin (PV).

(B and C) Nkx2-1+/PV+ prototypic neurons fired significantly faster than Nkx2-1+/PV− neurons (53.83 ± 3.9 versus 31.7 ± 4.7 spikes/s, respectively), and more regularly than Nkx2-1+/PV− neurons (CV2 of 0.36 ± 0.02 versus 0.44 ± 0.03).

(D) Schematic parasagittal sections (D, dorsal; C, caudal) denoting the locations within the GPe of all recorded PV+ and PV− prototypic neurons (n = 33 and 11 neurons, respectively).

(E) Proportions of response type (firing decrease, increase, or no significant change) during movement were similar for Nkx2-1+/PV+ neurons (left) and Nkx2-1+/PV− neurons (right).

Data are represented as mean ± SEM. **p < 0.01, *p < 0.05.

in prototypic neurons during movement mapped onto this molecular diversity. To address this, we divided prototypic neurons according to their expression of PV (Figure 6). We first compared the firing of PV+ and PV− prototypic neurons when the mice were at rest. PV-expressing neurons fired significantly faster ($p < 0.01$, t test; Figure 6B) and more regularly ($p < 0.05$, Mann-Whitney; Figure 6C) than PV− neurons, but both groups of prototypic neurons fired at higher rates and more regularly than arkypallidal neurons. We next tested whether PV+ and PV− prototypic neurons differed in their responses during movement. Both groups of prototypic neurons showed similar proportions of each response type ($p > 0.05$, Fisher's exact test; Figure 6E). This analysis suggests that selective expression of PV does not correlate with the heterogeneous responses of prototypic GPe neurons during movement.

Arkypallidal and Prototypic GPe Neurons Can Reliably Encode Movement with Changes in Firing Rate

Although arkypallidal and prototypic neurons differed in their average response polarities, both cell types appeared to encode spontaneous movements with changes in their firing rates. To address this quantitatively, we tested whether movements could be decoded (i.e., predicted) from the spike trains of the individual neurons. More specifically, we computed receiver operating characteristic (ROC) curves for each GPe neuron to test whether spike rate could correctly classify the occurrence of a movement (Figures 7A–7F). We determined that most arkypallidal and prototypic neurons were indeed able to encode movement with their firing rate (Figure 7G). To ensure that neurons were not simply performing at chance levels, we compared the area under the ROC curves (AUC) for each neuron to confidence intervals obtained by shuffling the corresponding movement periods; most (8/10) arkypallidal neurons and most (31/43) prototypic neurons predicted movement significantly above chance (Figures 7G and 7H). The average AUCs of arkypallidal and prototypic neurons predicting movement were not significantly different (Figure 7H), suggesting that, despite clear differences in response polarities and heterogeneity, both cell types encode movement in their firing rates to a similar level of accuracy. Among the prototypic neurons encoding movement, those that decreased their firing rate during movement had higher average firing rates at rest than those that increased their rate during movement (57.1 ± 5.7 and 34.8 ± 4.3 spikes/s, respectively, at rest; $p < 0.01$, t test; Figure 7G).

Classical schemes of basal ganglia function and dysfunction are essentially "rate models" in that they posit that changes in neuron firing rates are of special significance for the encoding and governance of voluntary action (DeLong, 1990; Wichmann

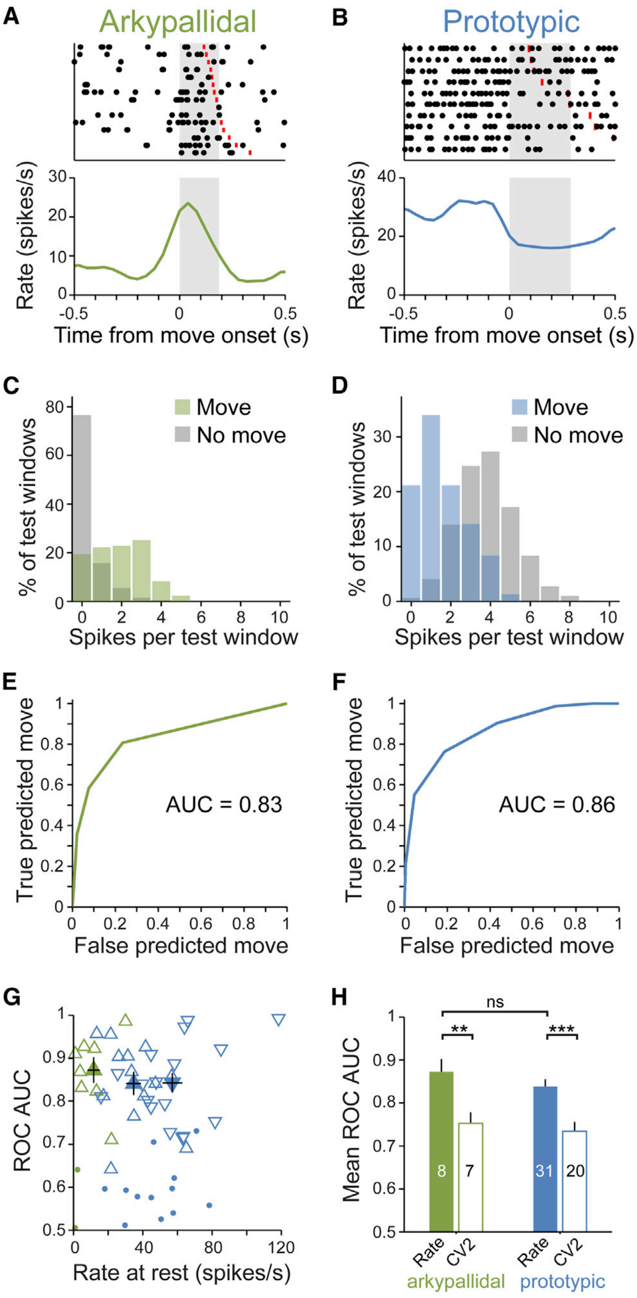

Figure 7. Both Arkypallidal and Prototypic Neurons Reliably Encode Movement

The ability of arkypallidal neurons and prototypic neurons to encode movement was assessed using receiver operating characteristic (ROC) analysis.

(A and B) Example PETH and raster plot for a single neuron of each cell type.

(C and D) Histograms for the same two neurons, showing the number of spikes in each test window classified as movement or non-movement.

(E and F) ROC curves for the same two neurons, showing the proportion of windows containing genuine movement and classified as movement from the spike train (a true prediction) versus the fraction of windows without movement and falsely classified as movement (false prediction) for each threshold value. An area under the ROC curve (AUC) of 0.5 corresponds to chance classification, while an AUC of 1 corresponds to perfect discrimination of movement.

(G) AUC plotted against firing rate during alert rest for all GPe neurons (arkypallidal in green, prototypic in blue). Filled circles represent individual neurons for which the AUC was not significantly different from shuffled data. Open triangles represent individual neurons that significantly encoded movement (△ = increased rate; ▽ = decreased rate). Mean values for significantly encoding neurons of each group are indicated by filled triangles.

(H) Mean AUCs for the arkypallidal (0.87 ± 0.03 for rate, 0.75 ± 0.03 for CV2) and prototypic neurons (0.84 ± 0.02 for rate, 0.73 ± 0.02 for CV2) able to significantly discriminate movement using firing rate or CV2 as a classifier (numbers of discriminating neurons are indicated within bars; $^{**}p < 0.01$; $^{***}p < 0.001$; ns, not significantly different).

Data are represented as mean ± SEM.

and DeLong, 1996). However, studies of human movement disorders (particularly Parkinson's disease) and their animal models emphasize that the temporal patterning of basal ganglia activity is also key (Bevan et al., 2002; Hammond et al., 2007; Nambu, 2008). With this in mind, we tested whether the movements we studied were better encoded by the rate or pattern (regularity) of firing of arkypallidal and prototypic neurons in the dopamine-intact GPe. We thus repeated the ROC analysis using a measure of regularity (CV2) as the classifier instead of firing rate. We determined that movement encoding was significantly less accurate when using CV2 as compared to rate for both arkypallidal and prototypic GPe neurons (t tests; Figure 7H). Moreover, fewer neurons performed significantly above chance when using firing regularity to classify movement. This analysis indicates that the firing rates of individual neurons in the dopamine-intact GPe are a more informative representation of movement. A final set of ROC analyses showed that, irrespective of whether firing rates or regularities were considered, PV+ and PV− prototypic neurons did not differ in their abilities to reliably encode movement (see Figure S6). In summary, our recordings of identified arkypallidal and prototypic GPe neurons in awake mice suggest that these two cell types are equally well suited to encoding movement, but, importantly, their accompanying readouts (i.e., the relative polarity and uniformity of their changes in firing rate) are fundamentally different.

DISCUSSION

Here, we define the developmental origins, molecular architecture, and behavioral significance of arkypallidal and prototypic neurons, the two main cell types of the GPe. We show that they are born in different regions of the embryonic subpallium and express different sets of postmitotic transcription factors. These differences are realized in behavior, with arkypallidal and prototypic neurons differentially encoding spontaneous movement in adult mice. We conclude that the distinct and specialized contributions of these two GPe cell types to voluntary actions are fated from early embryonic life.

Previous work in anesthetized Parkinsonian rats has established the concept of a functional dichotomy in the GPe, as embodied by two major GABAergic cell types that are defined on the basis of their distinct molecular, structural, and physiological properties (Mallet et al., 2012). Our data demonstrate that arkypallidal and prototypic neurons are defined by their mutually exclusive expression of FoxP2 and Nkx2-1, respectively (thus outlining a transcriptional "blueprint" for these cell

types), establish that arkypallidal neurons constitute one-fifth of all mouse GPe neurons, and show that this functional dichotomy exists in the dopamine-intact brain and extends to other species. Importantly, our results also help place reports of developmental heterogeneity in GPe neurons (Flandin et al., 2010; Nóbrega-Pereira et al., 2010) in the context of functionally defined cell types in the adult GPe. Thus, arkypallidal and prototypic neurons are born in the LGE/CGE and MGE, respectively, and then, during development, migrate and coalesce to form the GPe proper. Transcriptional codes are key for the generation, specification, migration, and synaptic maturation of neurons, as well as for maintaining the identities of postmitotic neurons (Arber, 2012; Deneris and Hobert, 2014; Jessell, 2000; Kepecs and Fishell, 2014; Siegert et al., 2012). Our data reveal one result of these developmental processes and terminal differentiation, as reflected in the functional organization of adult GPe. Because expression of transcription factors influences the intrinsic membrane properties (and hence, firing), structure, and connectivity of neurons (Butt et al., 2005; Kepecs and Fishell, 2014), it follows that such expression could ultimately govern the specialized roles played by different cell types in behavior. Indeed, the silencing or ablation of specific sets of spinal cord neurons has corroborated this (Arber, 2012; Goulding, 2009; Kiehn, 2011), but it has proven challenging to link transcriptional diversity to the encoding of movement by the spike firing of identified neurons in intact circuits. Here, we provide a unifying experimental framework that combines quantitative molecular profiling with genetic fate mapping and high-resolution sampling of the activity of individual, identified neurons in behaving animals. Using the GPe as an exemplar, we show that two neuron types with different transcriptional provenance play divergent roles in encoding movement on a timescale of milliseconds.

Electrophysiological diversity has long been observed in the GPe. Indeed, some of the first single-unit recordings in the GPe of primates distinguished two major groups of neurons based on their rates and patterns of firing (DeLong, 1971). According to one classification scheme adopted for recordings made in awake rats, monkeys, and Parkinsonian patients, the majority of GPe units are "high-frequency discharge" (HFD) neurons that pause, whereas the remaining ~15% are "low-frequency discharge bursting" (LFD-B) neurons (Benhamou et al., 2012; Elias et al., 2007; Gardiner and Kitai, 1992; Hutchison et al., 1994). Here, we provide the first definition of the firing of molecularly identified GPe neurons in awake, behaving animals. In doing so, we show that the firing rates and patterns of prototypic GPe neurons in the awake mouse at rest are similar to those of HFD neurons reported in other species. In contrast, the firing rates and patterns of arkypallidal neurons are within the ranges reported for LFD-B neurons, raising the possibility that they are the same cell type. However, although the average firing properties of prototypic and arkypallidal neurons are distinct, there is some overlap, and hence, the ability to correlate behavior-related firing with a molecularly defined cell type is especially valuable.

Prototypic GPe neurons exhibit relatively high firing rates when the animal is at rest and, thus, potentially have a wide dynamic range (large negative and positive activity modulations) for encoding behavior. In contrast, arkypallidal neurons fire at relatively low rates during rest, indicating they have less scope for negative activity modulations. In support of this, almost all prototypic neurons exhibited either decreases or increases in firing rates in time with movements. Moreover, and in contrast, arkypallidal neurons consistently encoded movement with robust increases in firing rate. In primates and rats, movement-related decreases and increases in the firing of GPe HFD neurons are frequently observed, although increases in activity tend to dominate; because the responses of HFD neurons are dependent on many different movement parameters, as well as whether the animal is engaged in a trained and/or rewarded task, we cannot rule out that the balance between increases and decreases in GPe neuron firing rate shifts according to the type of movement studied and a host of other factors (DeLong, 1971; Gardiner and Kitai, 1992; Georgopoulos et al., 1983; Jaeger et al., 1995; Mink and Thach, 1991; Mitchell et al., 1987; Turner and Anderson, 1997). Nevertheless, because we performed our recordings of identified GPe neurons in untrained mice that moved without overt external cueing, our data provide new insights into the cell-type-specific encoding of spontaneous or self-paced movements. It is also possible that the preferred response polarities of prototypic neurons are somewhat fixed in adulthood by genetics. If this were the case, then molecular markers could be exploited in the future to distinguish and functionally interrogate the prototypic neurons that decrease firing versus those that increase firing. However, our data indicate that Nkx2-1, Lhx6, PV, and Npas1 cannot be used for such a stratification.

What are the functional impacts of the changes in firing rates of prototypic and arkypallidal neurons during movement, and how do these changes arise? GABAergic prototypic GPe neurons innervate the STN as well as basal ganglia output nuclei and, occasionally, striatum (Bevan et al., 1998; Kita, 2007; Mallet et al., 2012). A decrease in their firing rate during movement would thus fit well with the proposed role of the GPe in the classical "indirect pathway," i.e., disinhibition of STN and output nuclei (Kravitz et al., 2010; Sano et al., 2013), which should ultimately inhibit unwanted actions or terminate action sequences (Gerfen and Surmeier, 2011). GABAergic striatopallidal neurons, which are active around movement onset (Cui et al., 2013; Isomura et al., 2013), are prime candidates for mediating the decreases in prototypic neuron firing during movement. Indeed, electrophysiological studies in vitro (Chuhma et al., 2011) and computational work (Nevado-Holgado et al., 2014) indicate that prototypic neurons receive comparatively large striatal inputs and are endowed with robust autonomous firing. The functional roles and circuit substrates for the firing rate increases of a minority of prototypic neurons are less clear, but such increases would be one essential part of a center-surround organization of activity (Mink, 1996; Nambu et al., 2002). Our study provides the first detailed insights into how arkypallidal neurons could potentially contribute to information processing during actions. Arkypallidal neurons do not project to the STN (or to the other "downstream" targets of prototypic neurons), but rather, exclusively and densely innervate the striatum (Mallet et al., 2012). In further contrast to prototypic GPe neurons, arkypallidal neurons are expected to display little firing in the absence of synaptic drive, and also to receive comparatively weak striatal inputs (Chuhma et al., 2011; Nevado-Holgado et al., 2014). As such, the firing of arkypallidal

neurons is predicted to be a better reflection of their excitatory inputs from STN (Mallet et al., 2012; Nevado-Holgado et al., 2014). Because arkypallidal neurons are positioned to release GABA and/or enkephalin onto many striatal projection neurons and interneurons (Mallet et al., 2012), and because they have low firing rates at rest and robustly increase activity around movement, they are theoretically suited to facilitate ongoing "action selection," i.e., by inhibiting large striatal regions, they could prevent competing actions and, thus, help expression of a desired action (Redgrave et al., 1999). The properties of arkypallidal neurons also make them suited to expedite the cessation of actions. The STN plays important roles in stopping actions, rapid switching between tasks, and delaying action onset to aid optimal action selection (Aron and Poldrack, 2006; Frank et al., 2007; Hikosaka and Isoda, 2010; Isoda and Hikosaka, 2008; Schmidt et al., 2013). Many of these roles would benefit from contemporaneous inhibition of striatum. Indeed, computational modeling suggests that the direct action of the STN on basal ganglia output nuclei might be insufficient to cancel a "go signal," and, thus, additional mechanisms are required to suppress such signals at the level of striatum (Schmidt et al., 2013). The distinct molecular signatures of prototypic and arkypallidal neurons offer tractable access points through which the causative contributions of these cell types to behavior can be defined in the future (using, for example, genetics-based approaches). In conclusion, schemes of the organization of basal ganglia circuits should endeavor to account for the functional dichotomy that is embodied by the arkypallidal and prototypic neurons of the GPe. Our results show that these two cell types are distinguished by their firing during rest and movement, establishing the importance of this dichotomy for normal behavior, and further suggest that this "division of labor" stems from an early prenatal stage of brain development.

EXPERIMENTAL PROCEDURES

Animals

All experimental procedures on animals were conducted in accordance with the Animals (Scientific Procedures) Act, 1986 (United Kingdom). Unless noted otherwise, 3- to 4-month-old male mice were used for all experiments. See Supplemental Experimental Procedures for further details.

Tissue Processing for Light Microscopy

Coronal sections (50 μm) containing the GPe (see Figure S1) were cut from each perfusion-fixed adult mouse brain. Expression of molecular markers by GPe neurons was assessed by indirect immunofluorescence (see Supplemental Experimental Procedures).

Stereological Sampling

A series of tiled, z-stacked images of immunolabeled neurons was acquired (to a depth of 12 μm from tissue surface) across the entire GPe in both hemispheres at three rostro-caudal levels (Figure S1). Unbiased stereological sampling was used to generate all cell counts from six GPe sections per adult mouse. Cell counts were pooled for each mouse, and then expression profiles were constructed using the mean value from three mice (see Supplemental Experimental Procedures).

In Vivo Electrophysiological Recording, Juxtacellular Labeling, and Data Analysis

For recordings of electrophysiological signals and behavior, mice were head-fixed using a stainless steel post (surgically implanted above the right pallidum) and positioned above a running wheel. Extracellular recordings of the action potentials ("spikes") fired by individual GPe neurons were made from 23 wild-type C57Bl6/J mice at rest and/or engaged in brief movements. After recording, each neuron was juxtacellularly labeled with Neurobiotin, recovered after perfuse fixation, and revealed with Cy3-conjugated streptavidin (Mallet et al., 2012). All recorded and identified GPe neurons were tested by indirect immunofluorescence for expression of Nkx2-1, FoxP2, and PV (see Supplemental Experimental Procedures).

Data were acquired and initially analyzed using Spike2 software (Cambridge Electronic Design). Regularity of firing was assessed using mean CV2 (Holt et al., 1996), and bursts were detected using a custom MATLAB (MathWorks) routine based on the Poisson surprise method (see Supplemental Experimental Procedures). To examine movement-related firing of GPe neurons, we focused our analyses on recordings containing ≥ 5 brief posture-adjusting movements (defined as those with < 1-s duration and involving limb movement). Movement onset was determined using a combination of the EMG (measured from cervical muscles) and videos of behavior (30 frames/s). To compare movement-related firing across neurons, peri-event time histograms (40-ms bin width) of GPe neuron activity were transformed to Z scores using the baseline mean firing and SD, where baseline was $-1{,}000$ to -200 ms relative to movement onset (use of this window avoided possible contamination of baseline with changes in activity just prior to onset). Changes in movement-related activity were considered significant when firing rate crossed a threshold of baseline mean ± 2 SD during the defined movement period. ROC curves were computed using firing rate and CV2 to obtain their distributions with and without movement. True positive was defined as genuine movement that was correctly classified as movement, and false positive was defined as a period of no movement that was incorrectly classified as movement (see Supplemental Experimental Procedures). AUC was considered significant if it was higher than the 95th percentile of AUCs computed from shuffled data.

Statistical Analyses

Before statistical comparison, a Shapiro-Wilk test was used to judge whether data sets were normally distributed ($p < 0.05$ to reject). For normally distributed data sets, comparisons were made using a Student's t test, whereas for all other data, Mann-Whitney rank-sum tests were used (significance $p < 0.05$; SigmaStat, Systat Software). Data are presented as mean ± SEM throughout.

SUPPLEMENTAL INFORMATION

Supplemental Information includes six figures and Supplemental Experimental Procedures and can be found with this article online at http://dx.doi.org/10.1016/j.neuron.2015.03.007.

AUTHOR CONTRIBUTIONS

P.D.D. and P.J.M. designed research; P.D.D. performed all electrophysiological experiments and related analyses; P.D.D. and I.C.D. developed recording and juxtacellular labeling in awake mice; P.D.D., J.T.L., J.M.D., F.N.G., N.M.D., N.K., S.J.B.B., and P.J.M. contributed to cell counts and fate-mapping experiments; R.B. and P.D.D. performed ROC analyses; P.J.M. supervised the whole project; P.D.D. and P.J.M. wrote the paper with input from all authors.

ACKNOWLEDGMENTS

This work was supported by the Medical Research Council UK (MRC; awards UU138197109 and MC_UU_12020/5), the Monument Trust Discovery Award from Parkinson's UK (grant J-0901), and an Investigator Award from the Wellcome Trust (101821 to P.J.M.). F.N.G. was supported by the MRC and a University of Oxford Clarendon Fund Scholarship, N.K. by a European Research Council Starting Grant under the European Community's Seventh Framework Program (grant agreement no. 207807), I.C.D. by the Wellcome Trust (086602), R.B. by the MRC, and S.J.B.B. by the Human Frontier Science Program (CDA0023/2008-C) and MRC (MR/K004387/1). We are grateful to G. Fishell, J.E. Johnson, and V. Pachnis for their gifts of mice for fate mapping, and to

S. McKnight and T. Kaneko for their gifts of antibodies. We thank N. Mallet, D. Dupret, J. Schiemann, L. Magno, R. Lopes, and G. Neves for their input during early stages, and K.C. Nakamura and A. Sharott for their scientific insights and help with MATLAB. We also thank E. Norman, L. Conyers, C. Johnston, J. Janson, D. Jelfs, P. Flaxman, and M.G. Oliveira for expert technical assistance.

Received: July 12, 2014
Revised: January 23, 2015
Accepted: February 14, 2015
Published: April 2, 2015

REFERENCES

Anastasiades, P.G., and Butt, S.J.B. (2011). Decoding the transcriptional basis for GABAergic interneuron diversity in the mouse neocortex. Eur. J. Neurosci. *34*, 1542–1552.

Anderson, M.E., and Turner, R.S. (1991). A quantitative analysis of pallidal discharge during targeted reaching movement in the monkey. Exp. Brain Res. *86*, 623–632.

Arber, S. (2012). Motor circuits in action: specification, connectivity, and function. Neuron *74*, 975–989.

Arkadir, D., Morris, G., Vaadia, E., and Bergman, H. (2004). Independent coding of movement direction and reward prediction by single pallidal neurons. J. Neurosci. *24*, 10047–10056.

Aron, A.R., and Poldrack, R.A. (2006). Cortical and subcortical contributions to stop signal response inhibition: role of the subthalamic nucleus. J. Neurosci. *26*, 2424–2433.

Benhamou, L., Bronfeld, M., Bar-Gad, I., and Cohen, D. (2012). Globus pallidus external segment neuron classification in freely moving rats: a comparison to primates. PLoS ONE *7*, e45421.

Bevan, M.D., Booth, P.A., Eaton, S.A., and Bolam, J.P. (1998). Selective innervation of neostriatal interneurons by a subclass of neuron in the globus pallidus of the rat. J. Neurosci. *18*, 9438–9452.

Bevan, M.D., Magill, P.J., Terman, D., Bolam, J.P., and Wilson, C.J. (2002). Move to the rhythm: oscillations in the subthalamic nucleus-external globus pallidus network. Trends Neurosci. *25*, 525–531.

Boraud, T., Bezard, E., Guehl, D., Bioulac, B., and Gross, C. (1998). Effects of L-DOPA on neuronal activity of the globus pallidus externalis (GPe) and globus pallidus internalis (GPi) in the MPTP-treated monkey. Brain Res. *787*, 157–160.

Butt, S.J.B., Fuccillo, M., Nery, S., Noctor, S., Kriegstein, A., Corbin, J.G., and Fishell, G. (2005). The temporal and spatial origins of cortical interneurons predict their physiological subtype. Neuron *48*, 591–604.

Chuhma, N., Tanaka, K.F., Hen, R., and Rayport, S. (2011). Functional connectome of the striatal medium spiny neuron. J. Neurosci. *31*, 1183–1192.

Cui, G., Jun, S.B., Jin, X., Pham, M.D., Vogel, S.S., Lovinger, D.M., and Costa, R.M. (2013). Concurrent activation of striatal direct and indirect pathways during action initiation. Nature *494*, 238–242.

DeLong, M.R. (1971). Activity of pallidal neurons during movement. J. Neurophysiol. *34*, 414–427.

DeLong, M.R. (1990). Primate models of movement disorders of basal ganglia origin. Trends Neurosci. *13*, 281–285.

DeLong, M.R., Crutcher, M.D., and Georgopoulos, A.P. (1985). Primate globus pallidus and subthalamic nucleus: functional organization. J. Neurophysiol. *53*, 530–543.

Deneris, E.S., and Hobert, O. (2014). Maintenance of postmitotic neuronal cell identity. Nat. Neurosci. *17*, 899–907.

Du, T., Xu, Q., Ocbina, P.J., and Anderson, S.A. (2008). NKX2.1 specifies cortical interneuron fate by activating Lhx6. Development *135*, 1559–1567.

Elias, S., Joshua, M., Goldberg, J.A., Heimer, G., Arkadir, D., Morris, G., and Bergman, H. (2007). Statistical properties of pauses of the high-frequency discharge neurons in the external segment of the globus pallidus. J. Neurosci. *27*, 2525–2538.

Ferland, R.J., Cherry, T.J., Preware, P.O., Morrisey, E.E., and Walsh, C.A. (2003). Characterization of Foxp2 and Foxp1 mRNA and protein in the developing and mature brain. J. Comp. Neurol. *460*, 266–279.

Flames, N., Pla, R., Gelman, D.M., Rubenstein, J.L.R., Puelles, L., and Marín, O. (2007). Delineation of multiple subpallial progenitor domains by the combinatorial expression of transcriptional codes. J. Neurosci. *27*, 9682–9695.

Flandin, P., Kimura, S., and Rubenstein, J.L.R. (2010). The progenitor zone of the ventral medial ganglionic eminence requires Nkx2-1 to generate most of the globus pallidus but few neocortical interneurons. J. Neurosci. *30*, 2812–2823.

Flandin, P., Zhao, Y., Vogt, D., Jeong, J., Long, J., Potter, G., Westphal, H., and Rubenstein, J.L.R. (2011). Lhx6 and Lhx8 coordinately induce neuronal expression of Shh that controls the generation of interneuron progenitors. Neuron *70*, 939–950.

Fogarty, M., Grist, M., Gelman, D., Marín, O., Pachnis, V., and Kessaris, N. (2007). Spatial genetic patterning of the embryonic neuroepithelium generates GABAergic interneuron diversity in the adult cortex. J. Neurosci. *27*, 10935–10946.

Frank, M.J., Samanta, J., Moustafa, A.A., and Sherman, S.J. (2007). Hold your horses: impulsivity, deep brain stimulation, and medication in parkinsonism. Science *318*, 1309–1312.

Gardiner, T.W., and Kitai, S.T. (1992). Single-unit activity in the globus pallidus and neostriatum of the rat during performance of a trained head movement. Exp. Brain Res. *88*, 517–530.

Georgopoulos, A.P., DeLong, M.R., and Crutcher, M.D. (1983). Relations between parameters of step-tracking movements and single cell discharge in the globus pallidus and subthalamic nucleus of the behaving monkey. J. Neurosci. *3*, 1586–1598.

Gerfen, C.R., and Surmeier, D.J. (2011). Modulation of striatal projection systems by dopamine. Annu. Rev. Neurosci. *34*, 441–466.

Goulding, M. (2009). Circuits controlling vertebrate locomotion: moving in a new direction. Nat. Rev. Neurosci. *10*, 507–518.

Hamada, I., DeLong, M.R., and Mano, N. (1990). Activity of identified wrist-related pallidal neurons during step and ramp wrist movements in the monkey. J. Neurophysiol. *64*, 1892–1906.

Hammond, C., Bergman, H., and Brown, P. (2007). Pathological synchronization in Parkinson's disease: networks, models and treatments. Trends Neurosci. *30*, 357–364.

Hikosaka, O., and Isoda, M. (2010). Switching from automatic to controlled behavior: cortico-basal ganglia mechanisms. Trends Cogn. Sci. *14*, 154–161.

Holt, G.R., Softky, W.R., Koch, C., and Douglas, R.J. (1996). Comparison of discharge variability in vitro and in vivo in cat visual cortex neurons. J. Neurophysiol. *75*, 1806–1814.

Hutchison, W.D., Lozano, A.M., Davis, K.D., Saint-Cyr, J.A., Lang, A.E., and Dostrovsky, J.O. (1994). Differential neuronal activity in segments of globus pallidus in Parkinson's disease patients. Neuroreport *5*, 1533–1537.

Isoda, M., and Hikosaka, O. (2008). Role for subthalamic nucleus neurons in switching from automatic to controlled eye movement. J. Neurosci. *28*, 7209–7218.

Isomura, Y., Takekawa, T., Harukuni, R., Handa, T., Aizawa, H., Takada, M., and Fukai, T. (2013). Reward-modulated motor information in identified striatum neurons. J. Neurosci. *33*, 10209–10220.

Jaeger, D., Gilman, S., and Aldridge, J.W. (1995). Neuronal activity in the striatum and pallidum of primates related to the execution of externally cued reaching movements. Brain Res. *694*, 111–127.

Jensen, P., and Dymecki, S.M. (2014). Essentials of recombinase-based genetic fate mapping in mice. Methods Mol. Biol. *1092*, 437–454.

Jessell, T.M. (2000). Neuronal specification in the spinal cord: inductive signals and transcriptional codes. Nat. Rev. Genet. *1*, 20–29.

Kepecs, A., and Fishell, G. (2014). Interneuron cell types are fit to function. Nature *505*, 318–326.

Kessaris, N., Fogarty, M., Iannarelli, P., Grist, M., Wegner, M., and Richardson, W.D. (2006). Competing waves of oligodendrocytes in the forebrain and postnatal elimination of an embryonic lineage. Nat. Neurosci. *9*, 173–179.

Kiehn, O. (2011). Development and functional organization of spinal locomotor circuits. Curr. Opin. Neurobiol. *21*, 100–109.

Kita, H. (2007). Globus pallidus external segment. Prog. Brain Res. *160*, 111–133.

Kravitz, A.V., Freeze, B.S., Parker, P.R.L., Kay, K., Thwin, M.T., Deisseroth, K., and Kreitzer, A.C. (2010). Regulation of parkinsonian motor behaviours by optogenetic control of basal ganglia circuitry. Nature *466*, 622–626.

Liodis, P., Denaxa, M., Grigoriou, M., Akufo-Addo, C., Yanagawa, Y., and Pachnis, V. (2007). Lhx6 activity is required for the normal migration and specification of cortical interneuron subtypes. J. Neurosci. *27*, 3078–3089.

Long, J.E., Cobos, I., Potter, G.B., and Rubenstein, J.L.R. (2009). Dlx1&2 and Mash1 transcription factors control MGE and CGE patterning and differentiation through parallel and overlapping pathways. Cereb. Cortex *19* (1), i96–i106.

Magno, L., Catanzariti, V., Nitsch, R., Krude, H., and Naumann, T. (2009). Ongoing expression of Nkx2.1 in the postnatal mouse forebrain: potential for understanding NKX2.1 haploinsufficiency in humans? Brain Res. *1304*, 164–186.

Magno, L., Kretz, O., Bert, B., Ersözlü, S., Vogt, J., Fink, H., Kimura, S., Vogt, A., Monyer, H., Nitsch, R., and Naumann, T. (2011). The integrity of cholinergic basal forebrain neurons depends on expression of Nkx2-1. Eur. J. Neurosci. *34*, 1767–1782.

Mallet, N., Micklem, B.R., Henny, P., Brown, M.T., Williams, C., Bolam, J.P., Nakamura, K.C., and Magill, P.J. (2012). Dichotomous organization of the external globus pallidus. Neuron *74*, 1075–1086.

Mink, J.W. (1996). The basal ganglia: focused selection and inhibition of competing motor programs. Prog. Neurobiol. *50*, 381–425.

Mink, J.W., and Thach, W.T. (1991). Basal ganglia motor control. I. Nonexclusive relation of pallidal discharge to five movement modes. J. Neurophysiol. *65*, 273–300.

Mitchell, S.J., Richardson, R.T., Baker, F.H., and DeLong, M.R. (1987). The primate globus pallidus: neuronal activity related to direction of movement. Exp. Brain Res. *68*, 491–505.

Miyoshi, G., Hjerling-Leffler, J., Karayannis, T., Sousa, V.H., Butt, S.J.B., Battiste, J., Johnson, J.E., Machold, R.P., and Fishell, G. (2010). Genetic fate mapping reveals that the caudal ganglionic eminence produces a large and diverse population of superficial cortical interneurons. J. Neurosci. *30*, 1582–1594.

Molyneaux, B.J., Arlotta, P., Menezes, J.R.L., and Macklis, J.D. (2007). Neuronal subtype specification in the cerebral cortex. Nat. Rev. Neurosci. *8*, 427–437.

Nambu, A. (2008). Seven problems on the basal ganglia. Curr. Opin. Neurobiol. *18*, 595–604.

Nambu, A., Tokuno, H., and Takada, M. (2002). Functional significance of the cortico-subthalamo-pallidal 'hyperdirect' pathway. Neurosci. Res. *43*, 111–117.

Nevado-Holgado, A.J., Mallet, N., Magill, P.J., and Bogacz, R. (2014). Effective connectivity of the subthalamic nucleus-globus pallidus network during Parkinsonian oscillations. J. Physiol. *592*, 1429–1455.

Nóbrega-Pereira, S., Gelman, D., Bartolini, G., Pla, R., Pierani, A., and Marín, O. (2010). Origin and molecular specification of globus pallidus neurons. J. Neurosci. *30*, 2824–2834.

Redgrave, P., Prescott, T.J., and Gurney, K. (1999). The basal ganglia: a vertebrate solution to the selection problem? Neuroscience *89*, 1009–1023.

Rubin, A.N., Alfonsi, F., Humphreys, M.P., Choi, C.K.P., Rocha, S.F., and Kessaris, N. (2010). The germinal zones of the basal ganglia but not the septum generate GABAergic interneurons for the cortex. J. Neurosci. *30*, 12050–12062.

Sachdev, R.N., Gilman, S., and Aldridge, J.W. (1989). Effects of excitotoxic striatal lesions on single unit activity in globus pallidus and entopeduncular nucleus of the cat. Brain Res. *501*, 295–306.

Sano, H., Chiken, S., Hikida, T., Kobayashi, K., and Nambu, A. (2013). Signals through the striatopallidal indirect pathway stop movements by phasic excitation in the substantia nigra. J. Neurosci. *33*, 7583–7594.

Schmidt, R., Leventhal, D.K., Mallet, N., Chen, F., and Berke, J.D. (2013). Canceling actions involves a race between basal ganglia pathways. Nat. Neurosci. *16*, 1118–1124.

Siegert, S., Cabuy, E., Scherf, B.G., Kohler, H., Panda, S., Le, Y.-Z., Fehling, H.J., Gaidatzis, D., Stadler, M.B., and Roska, B. (2012). Transcriptional code and disease map for adult retinal cell types. Nat. Neurosci. *15*, 487–495.

Smith, Y., Bevan, M.D., Shink, E., and Bolam, J.P. (1998). Microcircuitry of the direct and indirect pathways of the basal ganglia. Neuroscience *86*, 353–387.

Sussel, L., Marin, O., Kimura, S., and Rubenstein, J.L. (1999). Loss of Nkx2.1 homeobox gene function results in a ventral to dorsal molecular respecification within the basal telencephalon: evidence for a transformation of the pallidum into the striatum. Development *126*, 3359–3370.

Takahashi, K., Liu, F.-C., Hirokawa, K., and Takahashi, H. (2003). Expression of Foxp2, a gene involved in speech and language, in the developing and adult striatum. J. Neurosci. Res. *73*, 61–72.

Turner, R.S., and Anderson, M.E. (1997). Pallidal discharge related to the kinematics of reaching movements in two dimensions. J. Neurophysiol. *77*, 1051–1074.

Turner, R.S., and Anderson, M.E. (2005). Context-dependent modulation of movement-related discharge in the primate globus pallidus. J. Neurosci. *25*, 2965–2976.

Wichmann, T., and DeLong, M.R. (1996). Functional and pathophysiological models of the basal ganglia. Curr. Opin. Neurobiol. *6*, 751–758.

Zhao, Y., Flandin, P., Long, J.E., Cuesta, M.D., Westphal, H., and Rubenstein, J.L.R. (2008). Distinct molecular pathways for development of telencephalic interneuron subtypes revealed through analysis of Lhx6 mutants. J. Comp. Neurol. *510*, 79–99.

Neuron, Volume *86*

Supplemental Information

Distinct Developmental Origins Manifest in the Specialized Encoding of Movement by Adult Neurons of the External Globus Pallidus

Paul D. Dodson, Joseph T. Larvin, James M. Duffell, Farid N. Garas, Natalie M. Doig,
Nicoletta Kessaris, Ian C. Duguid, Rafal Bogacz, Simon J.B. Butt, and Peter J. Magill

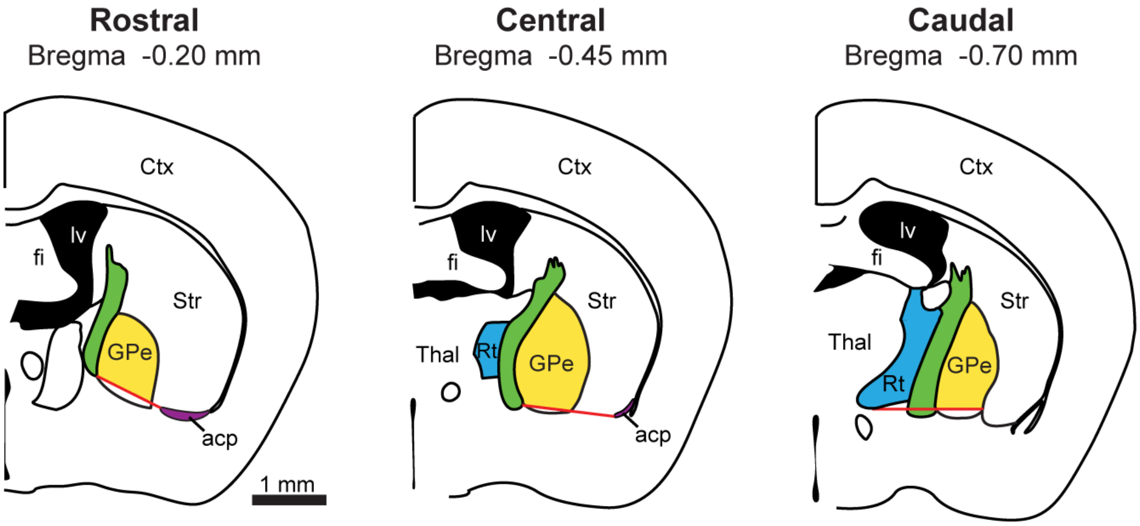

Figure S1 (related to Figures 1–3). Definition of GPe borders used for stereological cell counting. Coronal sections illustrating the rostral, central and caudal levels of the adult mouse external globus pallidus at which the molecular expression profiles of GPe neurons were quantified. For GPe neuron sampling in rostral and central sections, the ventral borders of GPe (red lines) were defined according to the medial edge of the anterior commissure (acp, in purple) and the bottom edge of the internal capsule (green). In caudal sections, the ventral border of GPe was defined according to the reticular nucleus of the thalamus (Rt, in blue). Only those neurons located dorsal to these borders were considered as GPe and counted (yellow). Ctx, cortex; fi, fimbria of the hippocampus; lv, lateral ventricle; Str, dorsal striatum; Thal, anterior thalamus. Schematics adapted from Paxinos and Franklin (2007); a standard stereotaxic reference (approximate distance posterior of Bregma) is given for each rostrocaudal level.

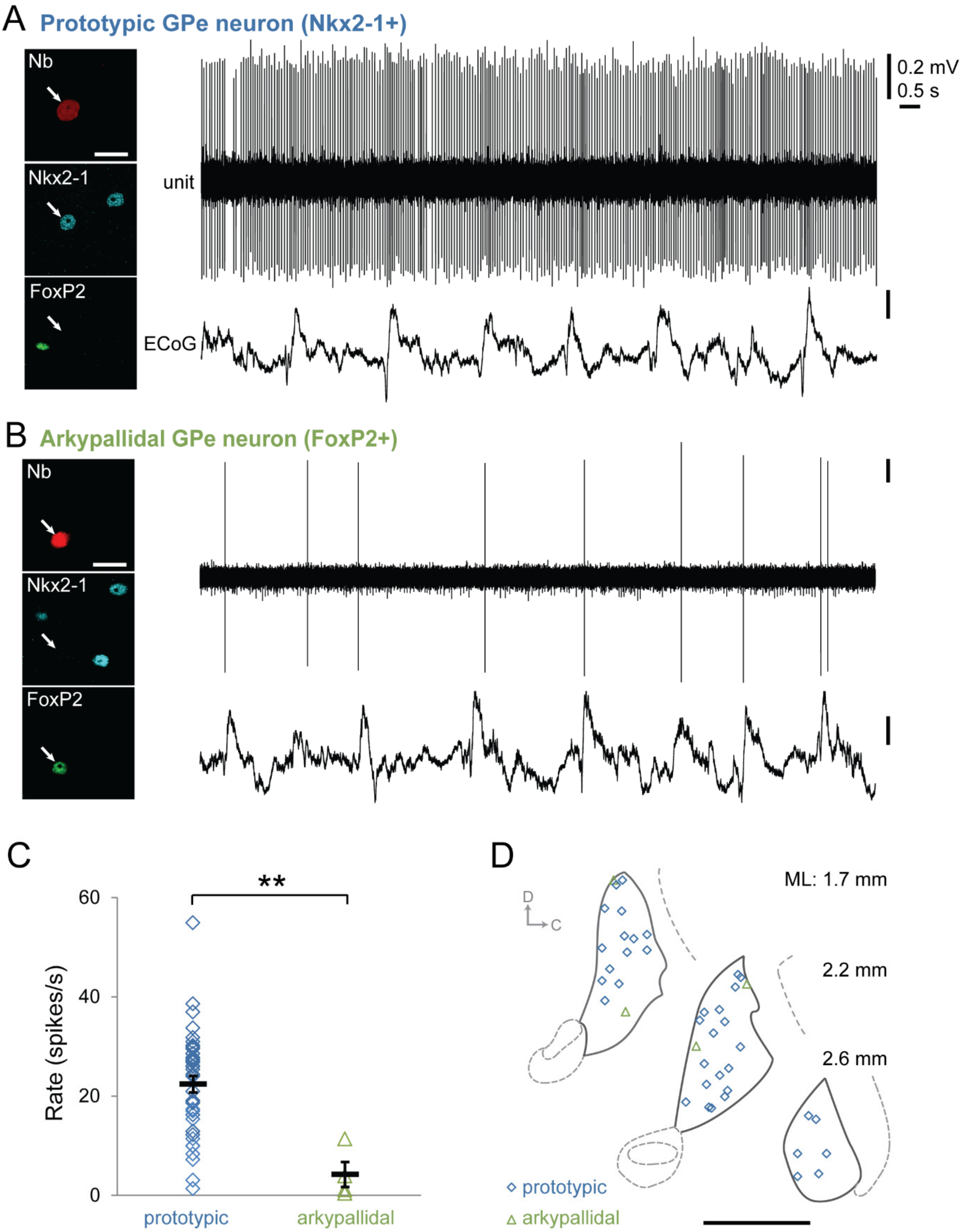
A
Prototypic GPe neuron (Nkx2-1+)
Nb
Nkx2-1
FoxP2
unit
ECoG
0.2 mV
0.5 s
B
Arkypallidal GPe neuron (FoxP2+)
Nb
Nkx2-1
FoxP2
C
**
60
40
20
0
Rate (spikes/s)
prototypic
arkypallidal
D
D
C
ML: 1.7 mm
2.2 mm
2.6 mm
prototypic
arkypallidal

Figure S2 (related to Figures 1–3). Arkypallidal and prototypic GPe neurons have distinct *in vivo* firing properties in anaesthetized mice.

(A and B) Typical single-unit activity (top) of a prototypic neuron (A) and an arkypallidal neuron (B). All GPe neurons were recorded during robust slow-wave activity, as exemplified by large amplitude, slow (~1 Hz) oscillations in the simultaneously-recorded electrocorticogram (ECoG, bottom). After recording, individual neurons were juxtacellularly labeled with Neurobiotin (Nb); prototypic neurons expressed Nkx2-1 (but not FoxP2) whereas arkypallidal neurons expressed FoxP2 (but not Nkx2-1). Verification of transcription factor expression by the same two example neurons is shown in left panels (scale bars: 20 μm).

C) Mean firing rate of prototypic neurons was significantly higher than that of arkypallidal neurons (22.4 ± 1.65 vs. 4.2 ± 2.5 spikes/s, n = 41 and 4 neurons, respectively; **p < 0.01, t-test). Data are presented as mean ± SEM.

(D) Schematic parasagittal sections (D, dorsal; C, caudal) denoting the locations within the GPe of all recorded and identified neurons. Mediolateral (ML) distance from Bregma is shown right. Scale bar: 1 mm.

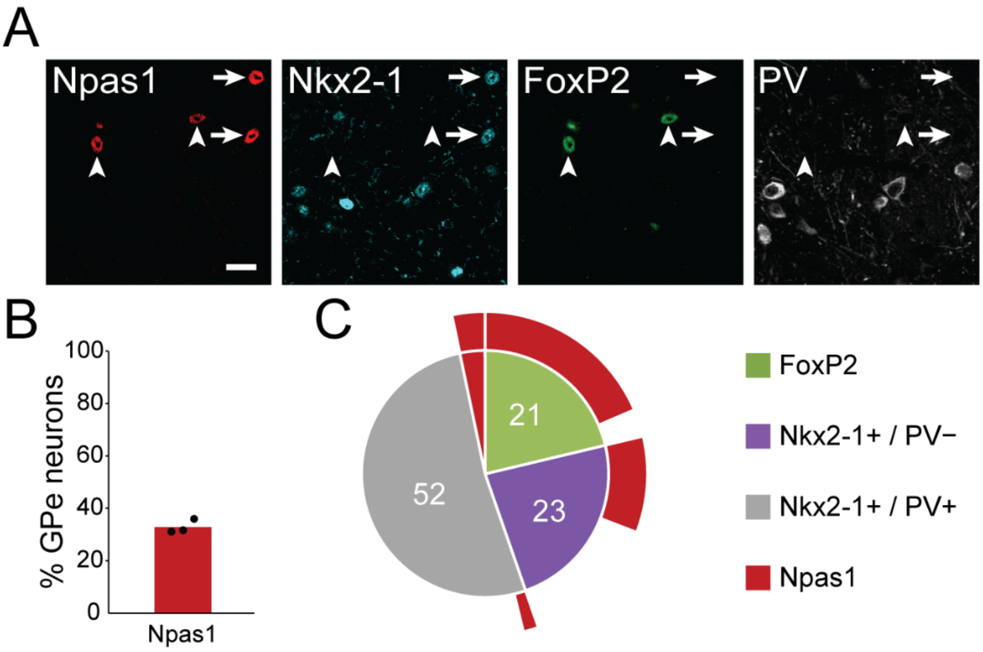

Figure S3 (related to Figures 1 and 2). Npas1 is often expressed by arkypallidal neurons but infrequently expressed by prototypic GPe neurons.

(A) Immunofluorescence labeling of GPe neurons in a wildtype mouse. Most FoxP2+ neurons co-express Npas1 (arrowheads). Some Nkx2-1+/PV− neurons (arrows) also express Npas1.

(B) Proportion of all GPe neurons (*i.e.* all HuCD+ neurons) expressing Npas1. Filled circles represent counts from individual animals.

(C) Proportions of GPe neurons expressing FoxP2, Nkx2-1 (with or without PV), or Npas1 alone. Note that co-expression of FoxP2 and Npas1 is common; the majority (87%) of FoxP2+ arkypallidal neurons co-express Npas1, and the majority of (56%) Npas1+ neurons co-express FoxP2. Also note that Npas1 is co-expressed by a minority (12%) of prototypic neurons (most Nkx2-1+/Npas1+ neurons are PV−). Only populations comprising ≥1% of GPe neurons are included.

Scale bar: 20 μm.

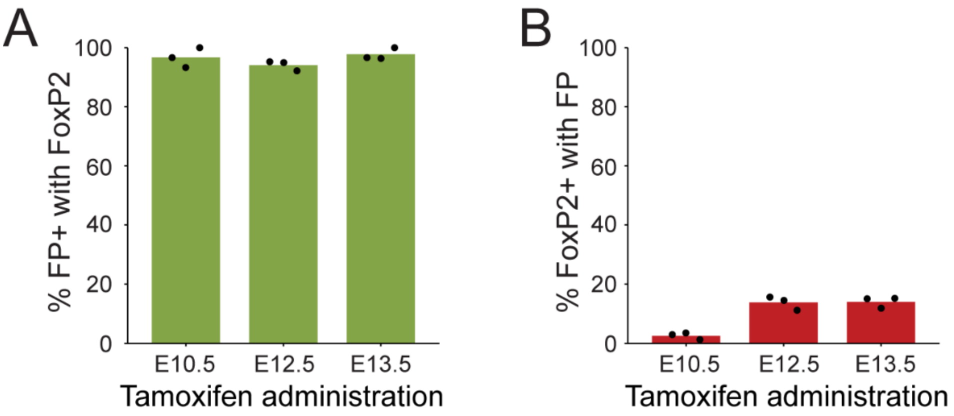

Figure S4 (related to Figure 3). The vast majority of GPe neurons derived from LGE/CGE express FoxP2.

(A) In *Mash1BAC-CreER;RCE* mice, which report neurons derived from LGE/CGE, the vast majority of GPe neurons expressing fluorescent protein (FP+) also expressed FoxP2; this held true across a range of embryonic time points for fate mapping in this mouse line. Tamoxifen was administered to pregnant *Mash1BAC-CreER;RCE* dams at one of three different embryonic time points (E10.5, E12.5 or 13.5), and co-expression of immunoreactivities was quantified in their offspring when they reached adulthood. Filled circles represent counts from individual animals.

(B) The proportion of FoxP2+ neurons co-expressing fluorescent protein in *Mash1BAC-CreER;RCE* mice is dependent upon the embryonic time point of tamoxifen administration. Note that, because of the limited duration and efficiency of tamoxifen-induced Cre activity, and because GPe neurons are generated over several days *in utero* (*i.e.* E10.5 to E13.5), much less than 100% of FoxP2+ neurons are expected to be captured at each time point.

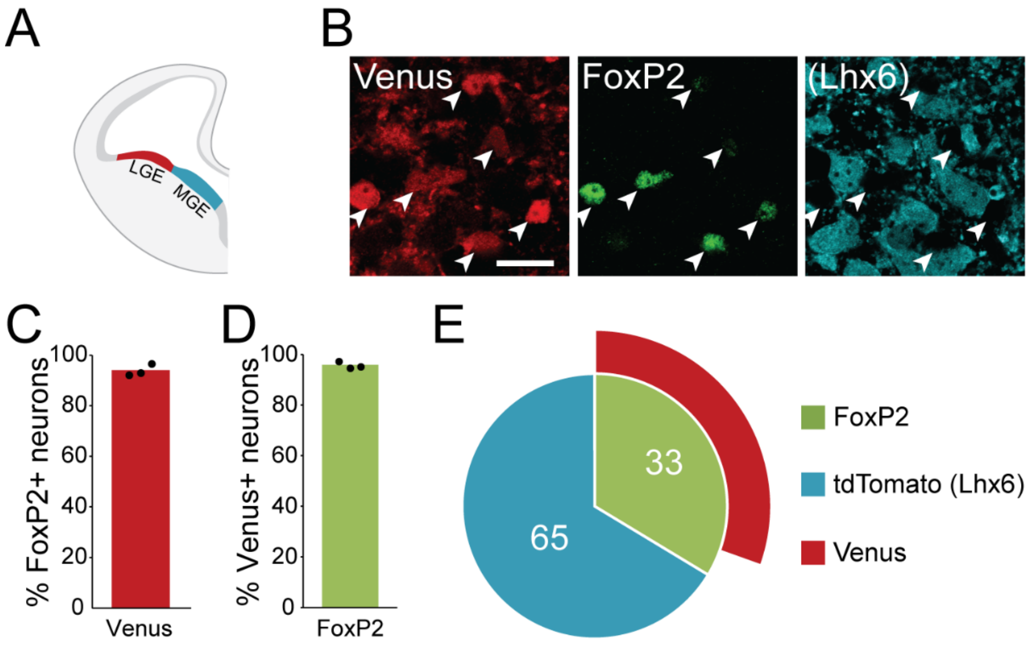

Figure S5 (related to Figure 3). FoxP2+ arkypallidal neurons derive from the LGE/CGE of the embryonic subpallium.

(A) Schematic of embryonic brain illustrating the progenitor domains that are labeled with different fluorescent proteins following a dual fate-mapping strategy using *Lhx6iCre;Ai9;Dlx1-Venus*^{fl} mice. Expression of Venus fluorescent protein (pseudocolored red) is driven from the ATG of the *Dlx1* gene in cells arising from the LGE/CGE and the MGE. The *Dlx1-Venus* transgene is floxed (fl). As such, in cells expressing *Lhx6iCre* (*i.e.* those originating in the MGE progenitor domain), the *Dlx1-Venus* transgene is excised, and the tdTomato fluorescent protein (pseudocolored teal) is instead expressed from the Rosa26 locus. Thus, neurons expressing Venus (but not tdTomato) are derived from the LGE/CGE, whereas tdTomato+ neurons are derived from the MGE. Because expression of the *Dlx1-Venus* transgene is down regulated in GPe neurons during the first postnatal week, cell counts were performed on the first postnatal day (P0.5).

(B) Immunofluorescence labeling of GPe neurons in a P0.5 *Lhx6iCre;Ai9;Dlx1-Venus*^{fl} mouse. The vast majority of Venus+ neurons co-expressed FoxP2, and vice versa (arrowheads); these neurons did not express tdTomato, the proxy marker for cells expressing, or that once expressed, Lhx6.

(C and D) Expression profiles of FoxP2+ neurons and Venus+ neurons. Filled circles represent counts from individual animals.

(E) Proportions of all GPe neurons (*i.e*. all HuCD+ neurons) expressing different markers. Note that the majority (97%) of FoxP2− neurons expressed tdTomato (teal). Only populations comprising ≥1% of GPe neurons are included.

Scale bar: 20 μm.

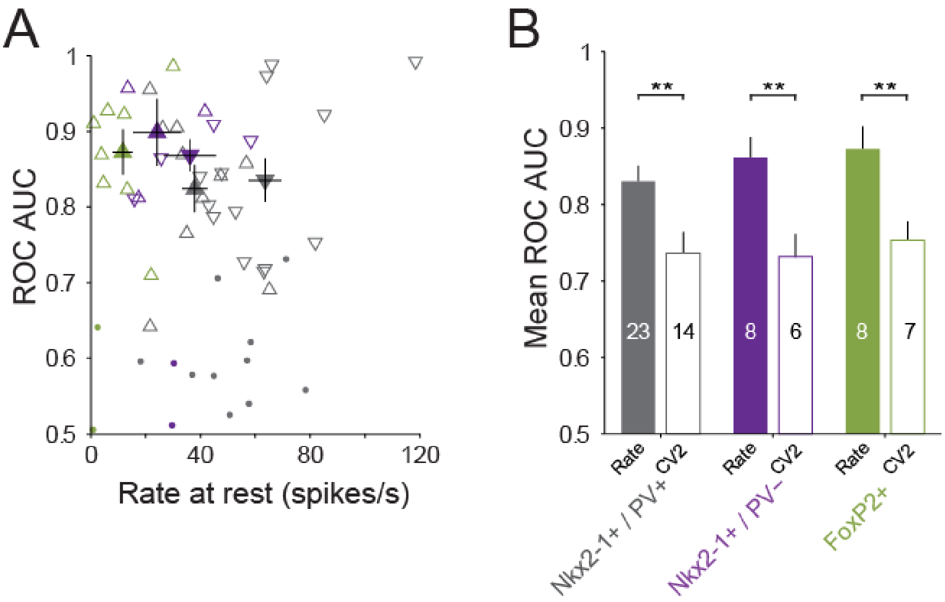

Figure S6 (related to Figure 7). Nkx2-1+/PV+ and Nkx2-1+/PV− prototypic neurons do not differ in their abilities to reliably encode movement.

The ability of Nkx2-1+/PV+ and Nkx2-1+/PV− prototypic neurons to encode movement was assessed using Receiver Operating Characteristic (ROC) analysis.

(A) Area under the ROC curve (AUC) plotted against firing rate during alert rest for all GPe neurons (Nkx2-1+/PV+ prototypic in grey, n = 33; Nkx2-1+/PV− prototypic in purple, n = 10; arkypallidal in green, n = 10). Filled circles represent individual neurons for which the AUC was not significantly different from shuffled data. Open triangles represent individual neurons that significantly encoded movement (Δ = increased rate; ∇ = decreased rate). Mean values for significantly encoding neurons of each group are indicated by filled triangles.

(B) Mean AUCs for Nkx2-1+/PV+ prototypic neurons (0.83 ± 0.02 for rate, 0.74 ± 0.03 for CV2), Nkx2-1+/PV− prototypic neurons (0.86 ± 0.02 for rate, 0.74 ± 0.02 for CV2), and arkypallidal neurons (0.87 ± 0.03 for rate, 0.75 ± 0.03 for CV2) able to significantly discriminate movement using firing rate or CV2 as a classifier (numbers of discriminating neurons are indicated within bars; **$p < 0.01$). Note that, irrespective of whether rates or CV2s were tested, there were no significant differences between the mean AUCs of Nkx2-1+/PV+, Nkx2-1+/PV− and arkypallidal neurons ($p > 0.05$; Kruskal-Wallis One-Way Analyses of Variance on ranks).

Data are represented as mean ± SEM.

SUPPLEMENTAL EXPERIMENTAL PROCEDURES

All experimental procedures on animals were conducted in accordance with the Animals (Scientific Procedures) Act, 1986 (United Kingdom). Unless noted otherwise, 3–4 month-old male mice were used for all experiments.

Animals

Stereological cell counts (Figures 1, S3 and 2) were performed using wildtype C57Bl6/J mice (Charles River) and hemizygous *Lhx6-EGFP* mice (STOCKTg(Lhx6-EGFP)BP221Gsat/Mmmh; Mutant Mouse Regional Resource Centers, USA), respectively. For fate-mapping experiments (Figures 3, S4 and S5) *Nkx2-1iCre;Z/EG* mice (Anastasiades and Butt, 2011) were generated by breeding hemizygous *Nkx2-1iCre* driver mice (Kessaris et al., 2006) with homozygous *Z/EG* reporter mice (Novak et al., 2000), *Lhx6iCre;RCE* mice were generated by breeding hemizygous *Lhx6iCre* driver mice (Fogarty et al., 2007) with homozygous *RCE:LoxP* reporter mice (Miyoshi et al., 2010), *Lhx6iCre;Ai9;Dlx1-Venus*^{fl} mice were generated by breeding hemizygous *Lhx6iCre*;*Ai9* driver/reporter mice (Fogarty et al., 2007; Madisen et al., 2010) with homozygous *Dlx1-Venus*^{fl} reporter mice (Rubin et al., 2010), and *Mash1BAC-CreER;RCE* mice were generated by crossing hemizygous *Mash1BAC-CreER* driver mice (Battiste et al., 2007; Miyoshi et al., 2010) with homozygous *RCE:LoxP* mice. To induce Cre-mediated recombination events after mating with male reporter mice, pregnant *Mash1BAC-CreER* dams were given 4 mg tamoxifen (Sigma, T5648; 20 mg/ml stock solution in corn oil) by gavage at E10.5, E12.5 or E13.5; the latter two stages were chosen to take advantage of most GPe neurons being born at E11-E12 (Nóbrega-Pereira et al., 2010). For staging of embryos, E0.5 was defined as noon of the day that the vaginal plug was detected.

Tissue processing for light microscopy

Mice were deeply anesthetized with pentobarbitone and transcardially perfused with 20 ml of 0.05 M phosphate-buffered saline, pH 7.4 (PBS), followed by 20 ml of fixative (4% paraformaldehyde in 0.1 M PB). Brains were then stored in fixative at 4ºC for ~24 h, before being sectioned (50 µm) in the coronal plane (Leica VT1000S). For studies in adult mice, brain sections containing rostral (−0.2 mm from Bregma), central (−0.45 mm) and caudal (−0.7 mm) aspects of GPe were selected

(Figure S1). For the supplementary study in *Lhx6iCre;Ai9;Dlx1-Venus*^{fl} mice at postnatal day (P) 0.5, two sections containing GPe were selected. After washing in PBS, sections were blocked in 'PBS-AzT' (PBS containing 0.02% sodium azide and TritonX-100 [0.3% or 0.03% for tissue from adult or neonates, respectively]) and 10% normal donkey serum (NDS) before being incubated overnight at room temperature in PBS-AzT containing 1% NDS and one or more of the following primary antibodies: rabbit anti-Nkx2-1 (1:500 dilution; Santa Cruz, sc-13040), mouse anti-Nkx2-1 (1:100; Leica Biosystems, Novacastra NCL-TTF-1), goat anti-FoxP2 (1:500; Santa Cruz, sc-21069; Reimers-Kipping et al., 2011), rabbit anti-Npas1 (1:500; gift of S.L. McKnight; Erbel-Sieler et al., 2004), guinea pig anti-parvalbumin (1:1000; Synaptic Systems, 195004), rat anti-EGFP (1:500; Nacalai Tesque, 04404), rabbit anti-PPE (1:5000; LifeSpan Biosciences, LS-C23084), mouse anti-human neuronal protein HuC/HuD ('HuCD'; 1:200; Life Technologies, A-21271). To optimize immunolabeling with mouse anti-Nkx2-1, rabbit anti-Npas1 and rabbit anti-PPE antibodies, we used a heat pre-treatment (2 h, 2 h, and 6 h, respectively) as a means of antigen retrieval (Mallet et al., 2012). In this study, all EGFP and Venus signals were enhanced using the anti-EGFP antibody detailed above. Expression of tdTomato in mice carrying the *Ai9* reporter allele (after Cre-mediated recombination) was revealed using a rabbit antibody raised against red fluorescent protein (1:1000; gift of T. Kaneko, mRFP1; Hioki et al., 2010). After washing off any unbound primary antibodies in PBS, sections were incubated overnight in appropriate secondary antibodies (all raised in donkey) that that were conjugated to the following fluorophores: AlexaFluor488 (1:500; Life Technologies), AMCA, AlexaFluor649 and Cy3 (1:250, 1:500 and 1:1000 respectively; Jackson ImmunoResearch).

Stereological sampling

Sections were scanned on an epifluorescence microscope (Zeiss Imager-M2) running Stereo Investigator software (MBF biosciences). Appropriate sets of filter cubes were used to image the fluorescence channels: AMCA (excitation 299-392 nm, beamsplitter 395 nm, emission 420-470 nm); AlexaFluor488 (excitation 450-490 nm, beamsplitter 495 nm, emission 500-550 nm); Cy3 (excitation 532-558 nm, beamsplitter 570 nm, emission 570-640 nm); and AlexaFluor649 (excitation 625-655 nm, beamsplitter 660 nm, emission 665-715 nm). Images of each of the channels were taken sequentially and separately to negate possible crosstalk of signal across channels. The borders of rostral, central and caudal GPe in adult mice were defined using a 5× 0.16 NA objective; a ventral border was conservatively assigned to separate the GPe from the functionally-distinct ventral pallidum (red lines in Figure S1). For the purposes of this study, it was

not necessary to determine absolute numbers or densities of GPe neurons expressing the molecular markers that were tested. We thus used a version of design-based stereology, the 'modified optical fractionator' (West, 1999). Stereological sampling was carried out over the entire GPe in both hemispheres, as sectioned at the designated rostral, central and caudal levels in adults or at the two levels in neonates. For each immunofluorescence protocol, a series of completely tessellated, z-stacked images were acquired using a 40× 1.3 NA oil-immersion objective and 1.0 μm steps ('optical sections') at depths of 2 to 12 μm from the upper surface of each section at the level of GPe. To minimize confounds arising from surface irregularities, neuropil within a 2 μm 'guard zone' at the upper surface was not imaged. This sampling strategy thus defined a 10-μm thick 'optical disector' that was used with abutting, unbiased rectangular counting frames (200 × 150 μm; consisting of two exclusion lines and two inclusion lines) to generate all cell counts and marker expression profiles (West, 1999). A neuron was only counted once through the series of optical sections when its nucleus came into sharp focus within the disector; neurons with nuclei already in focus in the top optical section of the disector were ignored. A neuron was classified as not expressing the tested molecular marker only when positive immunoreactivity could be observed in other cells on the same optical section as the tested neuron.

Electrophysiological recording and juxtacellular labeling of GPe neurons in awake mice

Twenty three adult C57BL/6j mice (Charles River) were head-fixed using a custom made stainless-steel post, surgically implanted above the right pallidum. For implantation, mice were anesthetized using 1–2% v/v isoflurane (Isoflo; Schering-Plough), and placed in a stereotaxic frame (Kopf). The analgesic buprenorphine (Vetergesic; 0.03 mg/kg, s.c.) was administered peri-operatively. Two 0.8 mm diameter steel screws were implanted in the skull, juxtaposed to the dura mater; one above the left frontal cortex (AP: 2 mm, ML: −2 mm, in relation to Bregma) and a reference screw above the left cerebellum. A coiled stainless-steel wire (AM systems) was implanted between the layers of cervical muscle to record electromyogram activity (EMG; filtered at 0.3–0.5 kHz, sampled at 20 kHz). The 'L-shaped' head-fixation post was adhered to the skull with cyanoacrylate adhesive (Loctite; Henkel UK). The post was positioned so that the 3 mm diameter hole in its base was above the right GPe (centered on AP: −0.4 mm, ML: +2.2 mm) and a discrete craniotomy was made within. The craniotomy was sealed with 'Kwik-cast' silicone sealant (World Precision Instruments) and the exposed skull and screws were encased in dental

acrylic (Jet Denture Repair; Lang Dental). The mice were then allowed to recover fully from surgery.

For electrophysiological recording, mice were placed on top of a custom 22 cm diameter Ethafoam running wheel and the head-restraint post attached to a stereotaxic frame using a custom holder. Extracellular recordings of action potentials (filtered at 0.3–5 kHz, gain of 1000×; ELX-01MX and DPA-2FS amplifiers from NPI Electronic Instruments; sampled as 20 kHz) fired by individual GPe neurons were made using glass electrodes (tip diameter ~1.3 µm; 10–30 MΩ *in situ*) containing 1.5% w/v Neurobiotin (Vector Labs) in 0.5 M NaCl (Janezic et al., 2013). Electrodes were lowered into the brain with submicron precision using a micromanipulator (IVM-1000; Scientifica). After recording, each neuron included here was juxtacellularly labeled with Neurobiotin. After allowing time for the diffusion of Neurobiotin within each neuron, the mouse was deeply anesthetized with pentobarbitone and transcardially perfused with fixative (see above).

To test the molecular profile of Neurobiotin-labeled GPe neurons, 50 µm parasagittal sections were incubated for 4 hrs at room temperature in PBS-AzT containing Cy3-conjugated streptavidin (1:3000; Life Technologies). Sections containing Neurobiotin-labeled neuronal somata were tested for immunoreactivity to molecular markers (see above); a neuron was classified as not expressing the tested marker only when positive immunoreactivity could be observed in other cells on the same focal plane as the Neurobiotin-labeled neuron.

Electrophysiological recording and juxtacellular labeling of GPe neurons in anesthetized mice

Extracellular recordings of individual GPe neurons were made in 12 anesthetized adult C57BL/6j mice. Anesthesia was induced with 1–2% v/v isoflurane and then maintained with urethane (1.5 g/kg, i.p.; ethyl carbamate, Sigma); supplemental doses of urethane (0.15 g/kg; i.p.) were given as required. Anesthetized mice were head fixed (see above) and laid upon a homeothermic heating mat (Harvard Apparatus). We focused our analysis on single-unit activity recorded in the GPe during robust slow-wave activity (SWA), as verified by simultaneous electrocorticogram (ECoG) recordings from the left frontal cortex (AP: 2 mm, ML: −2 mm). To extract SWA periods in an objective way, ECoG data were Fourier transformed (frequency resolution of 0.2 Hz), and the ratio of power in the SWA band (0.5–2 Hz) to power in the gamma band (30–80 Hz) was calculated. Epochs of contiguous data (each of >10 s in duration) where each ECoG data point

had a power ratio of >13 were then concatenated and used for further analysis (Janezic et al., 2013). After recording, all GPe neurons included here were juxtacellularly labeled with Neurobiotin and then recovered for immunohistochemical testing of molecular marker expression (see above).

Electrophysiological data analysis

Data were acquired and initially analyzed using Spike2 software (Cambridge Electronic Design). Putative single-unit activity was isolated with standard 'spike sorting' procedures, including template matching, principal component analysis, and supervised clustering (Spike2). Isolation of single units was verified by the presence of a distinct refractory period in the interspike interval (ISI) histograms. For analysis of the firing properties of neurons when mice were at rest, periods of movement were removed and the data were then concatenated. Mean firing rate (spikes/s) and mean CV2 were calculated from the total number of spikes. Mean CV2 is a measure of regularity (the lower the CV2 value, the more regular the unit activity) that is related to the coefficient of variation of the ISI but is less sensitive to alterations in firing rate (Holt et al., 1996): $CV2 = \sqrt{2}|(ISI_n - ISI_{n-1})| / (ISI_n + ISI_{n-1})$. Bursts were determined using a custom MATLAB (MathWorks) routine based on the Poisson surprise method (Elias et al., 2008; Legéndy and Salcman, 1985). For bursts, pairs of ISIs that had a mean ISI of less than half the mean ISI of the spike train were initially identified. From these 'seeds', the least probable group of successive spikes (compared to a Poisson spike train with the same mean rate, r) was determined. The Poisson surprise value (S) is the probability of n spikes in an epoch of T milliseconds such that $S = -\text{Log}\, P(n)$ where $P(n)$ is Poisson cumulative distribution function defined as follows:

$$\mathrm{P}(n) = e^{-rT} \sum_{i=n}^{n-1} (rT)^i / i!$$

Spikes following the seed were added to the seed one by one to maximize S; if addition of a spike failed to increase S, nine further attempts of adding subsequent spikes were made. Maximization of S was then attempted by omitting a spike from the beginning of the seed, if successful the process was iterated. Finally, further maximization of S was attempted by adding spikes preceding the seed (in a similar manner, but in the opposite direction, to the original spike addition process). The threshold for inclusion of the final burst period was set at $P(n)<0.05$ and data were only analyzed for bursts if recording epochs contained more than 60 spikes.

Peri-event time histograms (40 ms bin width) were smoothed with a sliding five-point Hamming window; varying bin size or smoothing parameters had no systematic effect on the results. To quantify how well the spike trains of individual GPe neurons could predict movement we used Receiver Operating Characteristic (ROC) analysis. We considered test epochs starting every 10 ms (the value of this parameter had no systematic effect on the results) and excluded epochs straddling the start or end of a movement period. To ensure that the test epoch was shorter than most movements (to avoid excluding movements from analysis), yet as long as possible (to maximize the amount of information within each epoch), we set the epoch on a neuron-by-neuron basis to be of a duration equal to half of the mean movement duration for the neuron being analyzed. For each epoch, we evaluated the firing rate and the regularity of firing (CV2) to obtain their distributions for epochs with and without movement. ROC curves were then constructed by plotting (for a series of rate or CV2 classification thresholds) the proportion of epochs containing movement and correctly classified as movement versus the fraction of epochs without movement but were falsely classified as movement. An area under the ROC curve (AUC) of 0.5 corresponds to chance classification, while an AUC of 1 corresponds to perfect discrimination. To assess statistical significance of the obtained AUC for a given neuron, a shuffling procedure was used. In each of the 1000 iterations, the shuffled data were created by shifting movement periods to random positions within the recording, and computing AUC for the shuffled data. The AUC for a neuron was considered significant if it was higher than the 95th percentile of the distribution of AUCs computed from shuffled data.

SUPPLEMENTAL REFERENCES

Anastasiades, P.G., and Butt, S.J.B. (2011). Decoding the transcriptional basis for GABAergic interneuron diversity in the mouse neocortex. Eur. J. Neurosci. *34*, 1542–1552.

Battiste, J., Helms, A.W., Kim, E.J., Savage, T.K., Lagace, D.C., Mandyam, C.D., Eisch, A.J., Miyoshi, G., and Johnson, J.E. (2007). Ascl1 defines sequentially generated lineage-restricted neuronal and oligodendrocyte precursor cells in the spinal cord. Development *134*, 285–293.

Elias, S., Ritov, Y., and Bergman, H. (2008). Balance of increases and decreases in firing rate of the spontaneous activity of basal ganglia high-frequency discharge neurons. J. Neurophysiol. *100*, 3086–3104.

Erbel-Sieler, C., Dudley, C., Zhou, Y., Wu, X., Estill, S.J., Han, T., Diaz-Arrastia, R., Brunskill, E.W., Potter, S.S., and McKnight, S.L. (2004). Behavioral and regulatory abnormalities in mice deficient in the NPAS1 and NPAS3 transcription factors. Proc. Natl. Acad. Sci. U. S. A. *101*, 13648–13653.

Fogarty, M., Grist, M., Gelman, D., Marín, O., Pachnis, V., and Kessaris, N. (2007). Spatial genetic patterning of the embryonic neuroepithelium generates GABAergic interneuron diversity in the adult cortex. J. Neurosci. *27*, 10935–10946.

Hioki, H., Nakamura, H., Ma, Y.F., Konno, M., Hayakawa, T., Nakamura, K.C., Fujiyama, F., and Kaneko, T. (2010). Vesicular glutamate transporter 3-expressing nonserotonergic projection neurons constitute a subregion in the rat midbrain raphe nuclei. J. Comp. Neurol. *518*, 668–686.

Janezic, S., Threlfell, S., Dodson, P.D., Dowie, M.J., Taylor, T.N., Potgieter, D., Parkkinen, L., Senior, S.L., Anwar, S., Ryan, B., et al. (2013). Deficits in dopaminergic transmission precede neuron loss and dysfunction in a new Parkinson model. Proc. Natl. Acad. Sci. U. S. A. *110*, E4016–E4025.

Kessaris, N., Fogarty, M., Iannarelli, P., Grist, M., Wegner, M., and Richardson, W.D. (2006). Competing waves of oligodendrocytes in the forebrain and postnatal elimination of an embryonic lineage. Nat. Neurosci. *9*, 173–179.

Legéndy, C.R., and Salcman, M. (1985). Bursts and recurrences of bursts in the spike trains of spontaneously active striate cortex neurons. J. Neurophysiol. *53*, 926–939.

Madisen, L., Zwingman, T.A., Sunkin, S.M., Oh, S.W., Zariwala, H.A., Gu, H., Ng, L.L., Palmiter, R.D., Hawrylycz, M.J., Jones, A.R., et al. (2010). A robust and high-throughput Cre reporting and characterization system for the whole mouse brain. Nat. Neurosci. *13*, 133–140.

Mallet, N., Micklem, B.R., Henny, P., Brown, M.T., Williams, C., Bolam, J.P., Nakamura, K.C., and Magill, P.J. (2012). Dichotomous organization of the external globus pallidus. Neuron *74*, 1075–1086.

Miyoshi, G., Hjerling-Leffler, J., Karayannis, T., Sousa, V.H., Butt, S.J.B., Battiste, J., Johnson, J.E., Machold, R.P., and Fishell, G. (2010). Genetic fate mapping reveals that the caudal ganglionic eminence produces a large and diverse population of superficial cortical interneurons. J. Neurosci. *30*, 1582–1594.

Nóbrega-Pereira, S., Gelman, D., Bartolini, G., Pla, R., Pierani, A., and Marín, O. (2010). Origin and molecular specification of globus pallidus neurons. J. Neurosci. *30*, 2824–2834.

Novak, A., Guo, C., Yang, W., Nagy, A., and Lobe, C. (2000). Z/EG, a double reporter mouse line that expresses enhanced green fluorescent protein upon Cre-mediated excision. Genesis *155*, 147–155.

Paxinos, G., and Franklin, K.B.J. (2007). The mouse brain in stereotaxic coordinates (Academic Press).

Reimers-Kipping, S., Hevers, W., Pääbo, S., and Enard, W. (2011). Humanized Foxp2 specifically affects cortico-basal ganglia circuits. Neuroscience *175*, 75–84.

Rubin, A.N., Alfonsi, F., Humphreys, M.P., Choi, C.K.P., Rocha, S.F., and Kessaris, N. (2010). The germinal zones of the basal ganglia but not the septum generate GABAergic interneurons for the cortex. J. Neurosci. *30*, 12050–12062.

West, M.J. (1999). Stereological methods for estimating the total number of neurons and synapses: issues of precision and bias. Trends Neurosci. *22*, 51–61.
